# Supplementary material for: Developing measures for WHO recommendations on antenatal care for a positive pregnancy experience: a conceptual framework and scoping review
Source: BMJ Open. 2019 Apr 24;9(4):e024130. doi: 10.1136/bmjopen-2018-024130 (PMC6502222; doi:10.1136/bmjopen-2018-024130)
Supplement: Supplementary file 1 [file bmjopen-2018-024130supp001.pdf]

Table A1: Current measures for ANC recommendations

Domain	WHO ANC Recommendation	Measure	Definition(s)	Numerator(s)	Denominator(s)
Content of Care	A.2.1 - Daily oral iron and folic acid supplementation with 30 mg to 60 mg of elemental iron and 400 g (0.4 mg) of folic acid is recommended for pregnant women to prevent maternal anaemia, puerperal sepsis, low birth weight, and preterm birth	Iron and folic acid supplements for pregnant women (%)	Percentage of pregnant women who received iron-folic acid supplementation for 90+ days[1 2]<br><br>Percent of women with a birth in the last two years who received *or bought* iron/folic acid supplements *for at least six months* during their last pregnancy in amounts that were in accordance with recommended protocols[3]<br><br>Proportion of pregnant women given iron and folic acid supplements[4]<br><br>Percentage of respondents who received any iron/folate during pregnancy[5]	Number of pregnant women who received the recommended number of iron/folic acid tablets during last pregnancy[1]<br><br>Number of pregnant women given 90+ IFA tablets[2]<br><br>Number of pregnant women who received *or purchased* the recommended number of iron/folic acid tablets during last pregnancy[3]<br><br>Number of pregnant women given iron and folic acid supplements[4]<br><br>Number of respondents who received any iron/folate during pregnancy[5]	Total number of pregnant women with a birth in last two years[1 3]<br><br>Number of pregnant women in health registers or monthly summary forms[2]<br><br>All eligible pregnancies (a woman having one documented ANC visit (unless the ANC visit is only for termination of pregnancy), OR any data documenting a pregnancy outcome or infant at any point of care)[4]<br><br>Total number of women surveyed who had delivered a live or stillbirth child during the year before the interview date[5]
Content of Care	A.2.1 - Daily oral iron and folic acid supplementation with 30 mg to 60 mg of elemental iron and 400 g (0.4 mg) of folic acid is recommended for pregnant women to prevent maternal anaemia, puerperal sepsis, low birth weight, and preterm birth	Preventive treatment with folic acid	Preventive treatment with folic acid: ratio of number of patients that completed folic acid prophylaxis to total number of pregnant women during the year[6]	Number of patients that completed folic acid prophylaxis	Total number of pregnant women during the year
Content of Care	A.2.1 - Daily oral iron and folic acid supplementation with 30 mg to 60 mg of elemental iron and 400 g (0.4 mg) of folic acid is recommended for pregnant women to prevent maternal anaemia, puerperal sepsis, low birth weight, and preterm birth	IFA supplementation	Mother consumed at least 100 iron-folic acid tablets during pregnancy[7 8]	Number of mothers who consumed at least 100 iron-folic acid tablets during pregnancy	Total number of study mothers (survey respondents who had a live birth in the reference period for each survey)
Content of Care	A.2.1 - Daily oral iron and folic acid supplementation with 30 mg to 60 mg of elemental iron and 400 g (0.4 mg) of folic acid is recommended for pregnant women to prevent maternal anaemia, puerperal sepsis, low birth weight, and preterm birth	ANC visits where the client received a 3-month supply of iron supplements and a 3-month supply of folate supplements	Number of ANC visits at facility per month where the client received a 3-month supply of iron supplements; Number of ANC visits at facility per month where the client received a 3-month supply of folate supplements[9]	Number of ANC visits per month where the client received a 3-month supply of iron supplements; Number of ANC visits at facility per month where the client received a 3-month supply of folate supplements	Total number of ANC visits at facility per month (= new visits + revisits)

Domain	WHO ANC Recommendation	Measure	Definition(s)	Numerator(s)	Denominator(s)
Content of Care	A.2.1 - Daily oral iron and folic acid supplementation with 30 mg to 60 mg of elemental iron and 400 g (0.4 mg) of folic acid is recommended for pregnant women to prevent maternal anaemia, puerperal sepsis, low birth weight, and preterm birth	Percentage of health facilities routinely providing iron and folic acid	Percentage of health facilities routinely providing iron and folic acid during ANC[10]	Number of health facilities where iron and folic acid supplementation is routinely provided during ANC	Total number of facilities surveyed
Content of Care	A.2.1 - Daily oral iron and folic acid supplementation with 30 mg to 60 mg of elemental iron and 400 g (0.4 mg) of folic acid is recommended for pregnant women to prevent maternal anaemia, puerperal sepsis, low birth weight, and preterm birth	Folic acid and iron supplementation in pregnancy	1. Women reporting any folic acid supplementation during their pregnancy; and 2. Women reporting any iron supplementation during their pregnancy[11]	1. Number of survey respondents reporting any folic acid supplementation during their pregnancy; 2. Number of survey respondents reporting any iron supplementation during their pregnancy<br><br>-OR- 1. Number of antenatal records reporting any folic acid supplementation during pregnancy; 2. Number of antenatal records reporting any iron supplementation during pregnancy	Total number of survey respondents<br><br>-OR- total number of antenatal records reviewed
Content of Care	A.2.2 - Intermittent oral iron and folic acid supplementation with 120 mg of elemental iron and 2800 g (2.8 mg) of folic acid once weekly is recommended for pregnant women to improve maternal and neonatal outcomes if daily iron is not acceptable due to side-effects, and in populations with an anaemia prevalence among pregnant women of less than 20%	Iron and folic acid supplements for pregnant women (%)	Percentage of pregnant women who received iron-folic acid supplementation for 90+ days[1]<br><br>Percent of women with a birth in the last two years who received *or bought* iron/folic acid supplements *for at least six months* during their last pregnancy in amounts that were in accordance with recommended protocols[3]	Number of pregnant women who received the recommended number of iron/folic acid tablets during last pregnancy[1]<br><br>Number of pregnant women who received *or purchased* the recommended number of iron/folic acid tablets during last pregnancy[3]	Total number of pregnant women with a birth in last two years
Health Systems	B.1.3 - Clinical enquiry about the possibility of intimate partner violence (IPV) should be strongly considered at antenatal care visits when assessing conditions that may be caused or complicated by IPV in order to improve clinical diagnosis and subsequent care, where there is the capacity to provide a supportive response (including referral where appropriate) and where the WHO minimum requirements are met	Percent of health units with at least one service provider trained to care for and refer SGBV survivors	Percent of health facilities in the geographic region of study (e.g., country, region, community) with at least one provider who has been trained within the past three years in the identification, care and support of sexual and gender-based violence (SGBV) survivors[3 12]	Number of health facilities reporting that they have both documented and adopted a protocol for the clinical management of SGBV survivors	Total number of health facilities surveyed

Domain	WHO ANC Recommendation	Measure	Definition(s)	Numerator(s)	Denominator(s)
Health Systems	B.1.3 - Clinical enquiry about the possibility of intimate partner violence (IPV) should be strongly considered at antenatal care visits when assessing conditions that may be caused or complicated by IPV in order to improve clinical diagnosis and subsequent care, where there is the capacity to provide a supportive response (including referral where appropriate) and where the WHO minimum requirements are met	Proportion of women who were asked about physical and sexual violence during a visit to a health unit	Proportion of women who presented to the clinic for any reason who were asked about physical or sexual violence, during a specific period of time (e.g., during the past 12 months)[12 13]	Number of women who were asked, during the course of their service provision at the unit, about any violence that had ever occurred, either physical or sexual, in the geographic area of study (nation, province, state, community).<br><br>If measured with a medical record review, all women's charts that note they were asked if they experienced any physical and sexual violence by a provider would be entered into the numerator.<br><br>If measured in a survey of women based on exit interviews from the health unit, all women leaving the clinic would be asked if a provider asked them if they had ever experienced any physical or sexual violence. All women answering yes would be entered into the numerator.	If the indicator is measured through a record review, this is the number of women's records that were reviewed at the health unit.<br><br>If the indicator is being measured through an exit interview, this is the total number of women interviewed.
Content of Care	B.1.5 - Health-care providers should ask all pregnant women about their tobacco use (past and present) and exposure to second-hand smoke as early as possible in the pregnancy and at every antenatal care visit	Proportion of pregnant women whose smoking status was checked at the first antenatal visit	Proportion of pregnant women whose smoking status was checked at the first antenatal visit[4]	Number of pregnant women whose smoking status was checked at the first ANC visit	Total number of eligible pregnancies (a woman having one documented ANC visit (unless the ANC visit is only for termination of pregnancy), OR any data documenting a pregnancy outcome or infant at any point of care)
Content of Care	B.1.5 - Health-care providers should ask all pregnant women about their tobacco use (past and present) and exposure to second-hand smoke as early as possible in the pregnancy and at every antenatal care visit	Cigarette smoking screened for in pregnancy	Percentage of women screened for smoking in pregnancy[14]	Number of medical records that indicate screening for smoking in pregnancy occurred	Total number of medical records reviewed

Domain	WHO ANC Recommendation	Measure	Definition(s)	Numerator(s)	Denominator(s)
Content of Care	B.1.7 - In high-prevalence settings, provider-initiated testing and counselling (PITC) for HIV should be considered a routine component of the package of care for pregnant women in all antenatal care settings. In low-prevalence settings, PITC can be considered for pregnant women in antenatal care settings as a key component of the effort to eliminate mother-to-child transmission of HIV, and to integrate HIV testing with syphilis, viral or other key tests, as relevant to the setting, and to strengthen the underlying maternal and child health systems	Pregnant women counselled and tested for HIV and know their results (%)	Percentage of pregnant women attending antenatal care (ANC), labour & delivery (L&D), and postpartum care services (PPC), plus women with known HIV infection attending ANC for a new pregnancy, who received testing and counselling for HIV in the last 12 months and who know their HIV test results[3]	The numerator can be summed from the categories below:<br>- Number of pregnant women who received an HIV test and result during ANC<br>- Number of pregnant women attending labour and delivery (L&D) with unknown HIV status who were tested in the L&D and received results<br>- Women with unknown HIV status attending postpartum services within 72 hours of delivery who were tested and received results<br>- Pregnant women with known HIV infection attending ANC for a new pregnancy.	The denominator is generated through a population estimate of the number of pregnant women giving birth in the last 12 months, which can be obtained from the Central Statistics Office estimates of births or the UN Population Division estimates. In countries with low-level and concentrated epidemics where policies to identify the HIV status of all pregnant women do not exist, the denominator should be adapted to the target population of pregnant women whose HIV status should be assessed.
Content of Care	B.1.7 - In high-prevalence settings, provider-initiated testing and counselling (PITC) for HIV should be considered a routine component of the package of care for pregnant women in all antenatal care settings. In low-prevalence settings, PITC can be considered for pregnant women in antenatal care settings as a key component of the effort to eliminate mother-to-child transmission of HIV, and to integrate HIV testing with syphilis, viral or other key tests, as relevant to the setting, and to strengthen the underlying maternal and child health systems	Number of pregnant women tested for HIV	Number of pregnant women attending ANC who are tested for HIV[15]<br><br>Number of ANC registrants receiving HIV testing and counselling[16]<br><br>Proportion of antenatal patients tested[17]<br><br>Proportion of pregnant women undergoing HIV testing and counselling during ANC[18]<br><br>Number (%) of newly registered antenatal cases tested for HIV[19]<br><br>Proportion of pregnant women who received testing and counselling at the first antenatal visit and received their results[4]<br><br>Proportion of pregnant women tested for HIV (of ANC1 and of total expected pregnant women)[20]<br><br>Proportion of pregnant women tested for HIV[2]	Total number of pregnant women attending ANC who are tested[15]<br><br>Number of ANC registrants receiving HIV testing and counselling[16]<br><br>Number of pregnant women with a new HTC test result documented in ANC clinic[18]<br><br>Number of newly registered antenatal cases tested for HIV[19]<br><br>Number of pregnant women who received testing and counselling at the first antenatal visit and received their results[4]<br><br>Number of pregnant women tested for HIV[2 20]	Total # of pregnant women attending ANC[15]<br><br>Total number of ANC registrants[16]<br><br>Total number of pregnant women presenting to ANC clinic without a prior documented positive HIV test result[18]<br><br>All new antenatal cases registered for the first time at any of the Integrated Counselling and Testing Centres that provided PPTCT services during any time of the antenatal period and also those cases who were registered for the first time at the time of admission during labour[19]<br><br>Total number of eligible pregnancies (a woman having one documented ANC visit (unless the ANC visit is only for termination of pregnancy), OR any data documenting a pregnancy outcome or infant at any point of care)[4]<br><br>Number of expected pregnant women[20]<br><br>Number of pregnant women in health registers or monthly summary forms[2]

Domain	WHO ANC Recommendation	Measure	Definition(s)	Numerator(s)	Denominator(s)
Content of Care	B.1.7 - In high-prevalence settings, provider-initiated testing and counselling (PITC) for HIV should be considered a routine component of the package of care for pregnant women in all antenatal care settings. In low-prevalence settings, PITC can be considered for pregnant women in antenatal care settings as a key component of the effort to eliminate mother-to-child transmission of HIV, and to integrate HIV testing with syphilis, viral or other key tests, as relevant to the setting, and to strengthen the underlying maternal and child health systems	Mothers of children 0-23 months who received HIV testing and counselling services during pregnancy (%)	% of mother of children 0-23 months who received HIV testing and counselling services during pregnancy[21]	# of mothers counselled and tested for HIV	Total # of mothers interviewed
Content of Care	B.1.7 - In high-prevalence settings, provider-initiated testing and counselling (PITC) for HIV should be considered a routine component of the package of care for pregnant women in all antenatal care settings. In low-prevalence settings, PITC can be considered for pregnant women in antenatal care settings as a key component of the effort to eliminate mother-to-child transmission of HIV, and to integrate HIV testing with syphilis, viral or other key tests, as relevant to the setting, and to strengthen the underlying maternal and child health systems	Percentage of health facilities routinely providing voluntary counselling and testing for HIV/AIDS	Percentage of health facilities routinely providing ANC clients with voluntary counselling and testing for HIV/AIDS[10]	Number of health facilities routinely providing ANC clients with voluntary counselling and testing for HIV/AIDS	Total number of facilities surveyed
Content of Care	B.1.7 - In high-prevalence settings, provider-initiated testing and counselling (PITC) for HIV should be considered a routine component of the package of care for pregnant women in all antenatal care settings. In low-prevalence settings, PITC can be considered for pregnant women in antenatal care settings as a key component of the effort to eliminate mother-to-child transmission of HIV, and to integrate HIV testing with syphilis, viral or other key tests, as relevant to the setting, and to strengthen the underlying maternal and child health systems	HIV testing in pregnant women	Percentage of pregnant women with known HIV status[22]	Number of pregnant women attending antenatal clinics (ANC) and/or had a facility-based delivery and were tested for HIV during pregnancy, or already knew they were HIV positive	Population-based denominator: Number of pregnant women who delivered within the past 12 months<br><br>Programme-based denominator: Number of pregnant women who attended an ANC or had a facility-based delivery in the past 12 months

Domain	WHO ANC Recommendation	Measure	Definition(s)	Numerator(s)	Denominator(s)
Content of Care	B.1.7 - In high-prevalence settings, provider-initiated testing and counselling (PITC) for HIV should be considered a routine component of the package of care for pregnant women in all antenatal care settings. In low-prevalence settings, PITC can be considered for pregnant women in antenatal care settings as a key component of the effort to eliminate mother-to-child transmission of HIV, and to integrate HIV testing with syphilis, viral or other key tests, as relevant to the setting, and to strengthen the underlying maternal and child health systems	Antenatal care: tested for syphilis (%)	Percent of women attending antenatal care services who were tested for syphilis[3]<br><br>Proportion of pregnant women tested for syphilis[2 20]<br><br>Proportion attending ANC at least once in a health facility where syphilis tests are available who report having received a test result for syphilis[23]	Number of women in antenatal care (ANC) who were screened for syphilis during pregnancy[3 24]<br><br>Number of women attending ANC services within the past 12 months who were tested for syphilis[25]<br><br>Number of pregnant women who were screened for syphilis at the first antenatal visit[4]<br><br>Number of pregnant women tested for syphilis[2 20]<br><br>Number of women with a live birth in the 12 months prior to the household survey who attended ANC at least once in a health facility where syphilis tests are available who report having received a test result for syphilis[23]	Number of pregnant women attending ANC clinics[3 24]<br><br>Number of women attending ANC services within the past 12 months[25]<br><br>Total number of eligible pregnancies (a woman having one documented ANC visit (unless the ANC visit is only for termination of pregnancy), OR any data documenting a pregnancy outcome or infant at any point of care)[4]<br><br>Number of expected pregnant women[20]<br><br>Number of pregnant women in health registers or monthly summary forms[2]<br><br>Number of women with a live birth in the 12 months prior to the household survey[23]
Content of Care	B.1.7 - In high-prevalence settings, provider-initiated testing and counselling (PITC) for HIV should be considered a routine component of the package of care for pregnant women in all antenatal care settings. In low-prevalence settings, PITC can be considered for pregnant women in antenatal care settings as a key component of the effort to eliminate mother-to-child transmission of HIV, and to integrate HIV testing with syphilis, viral or other key tests, as relevant to the setting, and to strengthen the underlying maternal and child health systems	Measures to ensure all pregnant women are screened and treated for syphilis	Existence of formal policy documents and national treatment protocols addressing maternal syphilis screening and treatment[26]	Number of countries with national-level policies addressing maternal syphilis screening and treatment	Total number of countries whose policies were reviewed

Domain	WHO ANC Recommendation	Measure	Definition(s)	Numerator(s)	Denominator(s)
Content of Care	B.2.1 - Daily fetal movement counting, such as with "count-to-ten" kick charts, is only recommended in the context of rigorous research	Antenatal care: fetal movement enquiry (%)	Number of pregnant women ≥28 weeks attending ANC who reported that antenatal care providers routinely enquired about fetal movement at each prenatal visit[27]<br><br>Proportion of survey respondents (gestational age >28 weeks) who reported that their lead maternity carer (LMC) regularly enquired about their baby's movements[28]	Number of pregnant women ≥28 weeks attending ANC who reported that antenatal care providers routinely enquired about fetal movement at each prenatal visit[27]<br><br>Number of pregnant women attending ANC (gestational age ≥28 weeks) who reported that their lead maternity carer regularly enquired about their baby's movements[28]	Total number of pregnant women ≥28 weeks attending ANC in the reporting period (who were surveyed)
Content of Care	C.1 - A seven-day antibiotic regimen is recommended for all pregnant women with asymptomatic bacteriuria (ASB) to prevent persistent bacteriuria, preterm birth and low birth weight	Number of episodes where immediate treatment was given per episode of positive urinalysis	Number of episodes where immediate treatment was given per episode of positive urinalysis[29]	Number of episodes of positive (abnormal) urinalysis where women received immediate treatment with antibiotics	Number of episodes of positive (abnormal) urinalysis in all charts reviewed
Content of Care	C.4 - In endemic areas, preventive anthelminthic treatment is recommended for pregnant women after the first trimester as part of worm infection reduction programmes	Percent of pregnant women who receive anthelminthic treatment during pregnancy	In areas of moderate to high endemicity of helminths (parasitic worms), the percent of pregnant women who receive presumptive anthelminthic treatment during their pregnancy. According to the 1998 IVACG/WHO/UNICEF "Guidelines for the Use of Iron Supplements to Prevent and Treat Iron Deficiency Anemia," treatment should be done once in the second and third trimester. In areas of low endemicity: the percent of pregnant women who received prescribed treatment during their pregnancy.[3]<br><br>Percentage of respondents who received deworming with albendazole during pregnancy[5]<br><br>Proportion of pregnant women provided deworming[2]	# of pregnant women who receive presumptive antihelminthic treatment[3]<br><br>Number of respondents who received deworming with albendazole during pregnancy[5]<br><br>Number of pregnant women provided deworming[2]	Total # of pregnant women[3]<br><br>Total number of women surveyed who had delivered a live or stillbirth child during the year before the interview date[5]<br><br>Number of pregnant women in health registers or monthly summary forms[2]
Content of Care	C.5 - Tetanus toxoid vaccination is recommended for all pregnant women, depending on previous tetanus vaccination exposure, to prevent neonatal mortality from tetanus	Percentage of newborns protected at birth from tetanus (takes into account mother's doses prior to pregnancy)	Percentage of newborns protected against tetanus[30]	Number of mothers with a live birth in the year prior to the survey who received two doses of tetanus toxoid vaccine within the appropriate interval prior to the infant's birth	Total number of women ages 15–49 with a live birth in the year prior to the survey
Content of Care	C.5 - Tetanus toxoid vaccination is recommended for all pregnant women, depending on previous tetanus vaccination exposure, to prevent neonatal mortality from tetanus	Percentage of health facilities offering tetanus toxoid vaccination services	Percentage of health facilities where tetanus toxoid vaccination services are available each day that ANC services are provided[10]	Number of health facilities where tetanus toxoid vaccination services are available each day that ANC services are provided	Total number of facilities surveyed

Domain	WHO ANC Recommendation	Measure	Definition(s)	Numerator(s)	Denominator(s)
Content of Care	C.5 - Tetanus toxoid vaccination is recommended for all pregnant women, depending on previous tetanus vaccination exposure, to prevent neonatal mortality from tetanus	Percent of women who received at least two doses of tetanus-toxoid vaccine in their last pregnancy	Proportion of pregnant women receiving at least two doses of tetanus-toxoid vaccine (TT2+)[2 3 7 8]<br><br>Percentage of respondents who received tetanus toxoid 2 doses during pregnancy or lifetime 5 doses[5]	Total TT2 + TT3 + TT4 + TT5 x 100 (Where TT2, TT3, TT4, TT5 refer to the 2nd, 3rd, 4th, or 5th dose of tetanus-toxoid vaccine administered)<br><br>Number of pregnant women given TT2 immunization[2]<br><br>Number of respondents who received tetanus toxoid 2 doses during pregnancy or lifetime 5 doses[5]<br><br>Number of respondents who received two or more TT injections[8]	Total # of live births<br><br>Number of pregnant women in health registers or monthly summary forms[2]<br><br>Total number of women surveyed who had delivered a live or stillbirth child during the year before the interview date[5]<br><br>Total number of ever married women surveyed in the age group 15-49 years[8]
Content of Care	C.5 - Tetanus toxoid vaccination is recommended for all pregnant women, depending on previous tetanus vaccination exposure, to prevent neonatal mortality from tetanus	Number of third trimester ANC visits protected against tetanus (at least TT2) per month	Number of third trimester (≥28 weeks) ANC visits protected against tetanus (at least TT2) per month[9]	Number of third trimester ANC visits protected against tetanus (at least TT2) per month	Total number of third trimester (>28 weeks) ANC visits at facility per month
Content of Care	C.5 - Tetanus toxoid vaccination is recommended for all pregnant women, depending on previous tetanus vaccination exposure, to prevent neonatal mortality from tetanus	Rate of tetanus vaccination	Rate of tetanus vaccination: ratio of number of pregnant women with complete vaccinations (verified at delivery) to total number of pregnant women during the year[6]	Number of pregnant women with complete vaccinations (verified at delivery)	Total number of pregnant women during the year
Content of Care	C.5 - Tetanus toxoid vaccination is recommended for all pregnant women, depending on previous tetanus vaccination exposure, to prevent neonatal mortality from tetanus	Mothers who received at least two tetanus toxoid vaccinations before the birth of their youngest child (%)	Percentage of mothers with children age 0-23 months who received at least two tetanus toxoid vaccinations before the birth of their youngest child[21]<br><br>Mothers of children 0-11 months who received ≥2 antenatal tetanus toxoid vaccinations[31]	# of Mothers who receiving at least 2 TT injections (Q19 + Q21=>2)[21]<br><br>Mothers of children 0-11 months who received ≥2 antenatal tetanus toxoid vaccinations[31]	Total # mothers interviewed[21]<br><br>Total number of mothers of children 0-11 months surveyed[31]

Domain	WHO ANC Recommendation	Measure	Definition(s)	Numerator(s)	Denominator(s)
Content of Care	C.6 - In malaria-endemic areas in Africa, intermittent preventive treatment with sulfadoxine-pyrimethamine (IPTp-SP) is recommended for all pregnant women. Dosing should start in the second trimester, and doses should be given at least one month apart, with the objective of ensuring that at least three doses are received	Intermittent preventive therapy for malaria during pregnancy (IPTp) (%)	Percentage of women who received three or more doses of intermittent preventive treatment during antenatal care visits during their last pregnancy	Number of women receiving three or more doses of recommended treatment[24]<br><br>Number of women who received three or more doses of a recommended prophylactic antimalarial drug treatment, at least one of which was received during an ANC visit, to prevent malaria during their last pregnancy that led to a live birth within the last two years[32]	Total number of pregnant women surveyed who delivered a live birth in the last 2 years
Content of Care	C.6 - In malaria-endemic areas in Africa, intermittent preventive treatment with sulfadoxine-pyrimethamine (IPTp-SP) is recommended for all pregnant women. Dosing should start in the second trimester, and doses should be given at least one month apart, with the objective of ensuring that at least three doses are received	Percentage of pregnant women attending ANC who receive a first (and/or second/third) dose of IPTp under direct observation	Proportion of women attending ANC clinics who receive IPT1/IPT2/IPT3 as directly observed treatment by a health worker to maximize compliance[33]	Number of pregnant women who receive IPT1 (IPT2/IPT3) under observation	Number of first ANC visits (stays the same regardless of IPT1/IPT2/IPT3)
Content of Care	C.6 - In malaria-endemic areas in Africa, intermittent preventive treatment with sulfadoxine-pyrimethamine (IPTp-SP) is recommended for all pregnant women. Dosing should start in the second trimester, and doses should be given at least one month apart, with the objective of ensuring that at least three doses are received	Number of third trimester ANC visits receiving IPT2 (or more) per month	Number of third trimester (≥28 weeks) ANC visits receiving IPT2 (or more) per month[9]	Number of third trimester ANC visits receiving IPT2 (or more) per month	Total number of third trimester (>28 weeks) ANC visits at facility per month
Content of Care	C.6 - In malaria-endemic areas in Africa, intermittent preventive treatment with sulfadoxine-pyrimethamine (IPTp-SP) is recommended for all pregnant women. Dosing should start in the second trimester, and doses should be given at least one month apart, with the objective of ensuring that at least three doses are received	Percentage of health facilities routinely providing preventative anti-malarial medication	Percentage of health facilities routinely providing preventative anti-malarial medication to ANC clients[10]	Number of health facilities routinely providing preventative anti-malarial medication to ANC clients	Total number of facilities surveyed

Domain	WHO ANC Recommendation	Measure	Definition(s)	Numerator(s)	Denominator(s)
Content of Care	C.7 - Oral pre-exposure prophylaxis (PrEP) containing tenofovir disoproxil fumarate (TDF) should be offered as an additional prevention choice for pregnant women at substantial risk of HIV infection as part of combination prevention approaches	PrEP coverage	% using PrEP in priority PrEP populations[25]	Number of members of the selected PrEP priority groups using PrEP within the last 12 months	Number of people in the selected PrEP priority groups
Health Systems	E.1 - It is recommended that each pregnant woman carries her own case notes during pregnancy to improve continuity, quality of care and her pregnancy experience	Percent of mothers of children 0-23 months with a maternal health card	Percent of mothers of children 0-23 months with a maternal health card (interviewer-confirmed)[21]	# of mothers with maternal health cards	Total # of mothers interviewed
Health Systems	E.6 - Policy-makers should consider educational, regulatory, financial, and personal and professional support interventions to recruit and retain qualified health workers in rural and remote areas	Health worker density and distribution	Number of health workers per 1000 population[24]	Number of health workers by cadre	Total population
Health Systems	E.7 - Antenatal care models with a minimum of eight contacts are recommended to reduce perinatal mortality and improve women's experience of care	Antenatal care (eight or more visits) (%)	Percentage of women aged 15–49 years with a live birth in a given time period who received antenatal care, eight times or more[1]	Number of women aged 15–49 years with a live birth in a given time period who received antenatal care eight or more times	Total number of women aged 15–49 years with a live birth in the same period<br><br>All female respondents in the household who have reached he age of menstruation (12 years and older) and were/are pregnant in the last 12 months[34]
Content of Care	Counselling about family planning	Percentage of health facilities routinely counselling ANC clients about family planning	Percentage of health facilities routinely counselling ANC clients about family planning[10]	Number of health facilities routinely counselling ANC clients about family planning	Total number of facilities surveyed
Content of Care	Counselling about family planning	Counselling on family planning	Percentage of pregnant women counselled on family planning[2]	Number of pregnant women counselled on family planning	Number of pregnant women in health registers or monthly summary forms
Content of Care	Blood pressure monitored	Blood pressure: At least six measures in routine antenatal care programme	National guidelines recommending at least six measures of blood pressure in routine antenatal care[35]	Number of national guidelines recommending at least six measures of blood pressure in routine antenatal care	Number of national guidelines reviewed

Domain	WHO ANC Recommendation	Measure	Definition(s)	Numerator(s)	Denominator(s)
Content of Care	Blood pressure monitored	Blood pressure: At least # measure(s) during pregnancy	Woman-reported content of antenatal care received at least once (or "x" times) during pregnancy: blood pressure measured	Number of women with at least one antenatal contact with any provider during pregnancy who reported that their blood pressure was measured at least once during pregnancy[36]<br><br>Blood pressure checked at least five times during antenatal care[37]	(1) all women with a live birth in the last 12 months prior to the survey; or (2) women who had at least one antenatal contact with any provider during that pregnancy[36]<br><br>All mothers in the sample who underwent prenatal care and delivery in the public health service whose children were born alive[37]
Content of Care	Blood pressure monitored	ANC visits with blood pressure reading documented	Number of ANC visits at facility per month with blood pressure reading documented[9]	Number of ANC visits per month with a documented blood pressure reading	Total number of ANC visits at facility per month (= new visits + revisits)
Content of Care	Blood pressure monitored	Receipt of basic ANC services	Receipt of all three basic services during antenatal care: blood pressure measurement, blood sample taken, urine sample taken[38]	Number of women surveyed who received all three basic services during antenatal care: blood pressure measurement, blood sample taken, urine sample taken	Total number of women surveyed
Content of Care	Blood pressure monitored	Blood pressure measured at last exam	Percentage of women who had their blood pressure measured at their most recent ANC visit	Number of survey/interview respondents reporting that their blood pressure was measured at their most recent ANC visit<br><br>Number of pregnant women with blood pressure recorded[2]	Total number of pregnant women using ANC during the data collection period who consented and were selected by systematic random sampling at the health facilities[39]<br><br>Total number of client exit interviews[40]<br><br>Number of pregnant women in health registers or monthly summary forms[2]

Domain	WHO ANC Recommendation	Measure	Definition(s)	Numerator(s)	Denominator(s)
Health Systems	First antenatal care visit in the first trimester	Percentage of pregnant women who began ANC during the first trimester of gestation	Proportion of pregnant women who initiate ANC in the first trimester[41]<br><br>Proportion of women with estimated gestational age at first antenatal visit < 12 weeks[42]<br><br>Proportion of women who receive antenatal assessments by 13 weeks of pregnancy[43]<br><br>Antenatal care initiated in the first trimester (<14 weeks) and minimum of 4 visits[44]<br><br>Percentage of women among whom the first medical visit occurred in the first trimester of pregnancy (or timeliness)[45]<br><br>Mothers recorded in the antenatal records with ANC in first trimester (<13 weeks)[46]<br><br>Women presenting in first trimester for first antenatal visit[14]	Number of pregnant women initiating ANC in first trimester[41]<br><br>Number of women attending ANC who began ANC during the first trimester[14]<br><br>Number of women whose first antenatal care visit was <12 weeks of gestational age[47]<br><br>Number of women whose first prenatal visit was ≤12 weeks[48]<br><br>Number of women with estimated gestational age at first antenatal visit < 12 weeks[42]<br><br>Number of pregnant women seen for ANC booking by 13 weeks 0 days[43]<br><br>Antenatal care initiated in the first trimester (<14 weeks) and minimum of 4 visits[44]<br><br>Number of women aged 15-49 surveyed who reported an obstetric episode from 2004-2009 and whose first medical visit occurred in the first trimester of pregnancy[45]<br><br>Mothers recorded in the antenatal records with ANC in first trimester (<13 weeks)[46]	Total number of women attending ANC[49]<br><br>All female respondents in the household who have reached he age of menstruation (12 years and older) and were/are pregnant in the last 12 months[34]<br><br>Total number of pregnant women using ANC during the data collection period who consented and were selected by systematic random sampling at the health facilities[39]<br><br>Total number of survey respondents; also total number of respondent electronic records[47]<br><br>Total number of medical charts reviewed[14 42 46 48]<br><br>Number of pregnant women[43]<br><br>Number of women sampled[44]<br><br>Total number of women aged 15-49 surveyed who reported an obstetric episode from 2004-2009[45]
Health Systems	First antenatal care visit in the first trimester	Timing of first antenatal visit (%)	Percentage of pregnant women aged 15–49 years who had their first antenatal visit in the first trimester[1]	Number of pregnant women aged 15-49 who had their first antenatal visit in the first trimester	Total number of women aged 15-49 years with at least one antenatal care visit in the same period

Domain	WHO ANC Recommendation	Measure	Definition(s)	Numerator(s)	Denominator(s)
Content of Care	Counselling about birth preparedness	Receiving ANC counselling about birth preparedness	Percentage of pregnant women who received at least one counselling session on birth preparedness	Number of women who had at least one antenatal contact with any provider during that pregnancy and report they were counselled about birth preparedness at least once during pregnancy[36]<br><br>Number of pregnant women counselled on emergency preparedness[2]	(1) all women with a live birth in the last 12 months prior to the survey; or (2) women who had at least one antenatal contact with any provider during that pregnancy[36]<br><br>Number of pregnant women in health registers or monthly summary forms[2]
Content of Care	Counselling about birth preparedness	Exposure to birth preparedness messages	(a) formal exposure, i.e. a respondent who received a key chain and/or was counselled using a birth preparedness flip-chart; and (b) exposure to specific birth preparedness messages, i.e. a respondent who reported having heard a specific message[50]	Formal exposure: total number of respondents who received a key chain and/or were counselled using a birth preparedness flip-chart<br><br>Message exposure: Total number of respondents exposed to specific birth preparedness messages: (a) "A pregnant women should make four antenatal care visits with a trained health worker"; (b) "A newborn should be breastfed for the first time immediately after birth"; and, (c) "A mother and newborn should have their health checked by a trained health worker within days after birth"	Total number of mothers of live infants aged less than one year at the time of the survey

*Note: Eight of the 58 resources (references 51-58) generated by the scoping review do not appear in the truncated table above. These eight resources are tools specific to the content of ANC and may contribute to the collection of multiple measures, providing important information about indicator methodology (e.g. method of measurement, measurement frequency, data sources) and current methodological work in their linked tabulation plans and manuals.[51-58]

References identified by the scoping review

1. MoNITOR. Maternal and Newborn Indicator Mapping. Geneva: World Health Organization, 2017.

2. Dwivedi V, Drake M, Rawlins B, et al. A Review of the Maternal and Newborn Health Content of National Health Management Information Systems in 13 Countries in Sub-Saharan Africa and South Asia. Washington, D.C.: Maternal and Child Health Integrated Program, 2014.

3. MEASURE Evaluation. Summary List of Indicators Chapel Hill, North Carolina, USA: MEASURE Evaluation; 2017 [Available from: https://www.measureevaluation.org/prh/rh_indicators/indicator-summary accessed 27 Aug 2017.

4. Flenady V, Wojcieszek AM, Fjeldheim I, et al. eRegistries: indicators for the WHO Essential Interventions for reproductive, maternal, newborn and child health. *Bmc Pregnancy and Childbirth* 2016;16 doi: 10.1186/s12884-016-1049-y

5. Hodgins S, McPherson R, Suvedi BK, et al. Testing a scalable community-based approach to improve maternal and neonatal health in rural Nepal. *Journal of perinatology : official journal of the California Perinatal Association* 2010;30(6):388-95. doi: 10.1038/jp.2009.181 [published Online First: 2009/11/13]

6. Marin GH, Silberman M, Uriarte A, et al. Healthcare model based on personalised attention: impact on maternal mortality and health system quality. *Quality in primary care* 2011;19(5):311-6. [published Online First: 2011/12/22]

7. Baqui AH, Rosecrans AM, Williams EK, et al. NGO facilitation of a government community-based maternal and neonatal health programme in rural India: improvements in equity. *Health Policy and Planning* 2008;23(4):234-43. doi: 10.1093/heapol/czn012

8. Kavitha N. Are Young Mothers in India Deprived of Maternal Health Care Services? A Comparative Study of Urban and Rural Areas. *Journal of Health Management* 2015;17(2):204-20. doi: http://dx.doi.org/10.1177/0972063415575809

9. Mwaniki MK, Vaid S, Chome IM, et al. Improving service uptake and quality of care of integrated maternal health services: the Kenya Kwale District improvement collaborative. *BMC health services research* 2014;14:416. doi: 10.1186/1472-6963-14-416 [published Online First: 2014/09/23]

10. Keating J. Nigeria Baseline Health Facility Survey, 2005: [Chapel Hill, North Carolina], University of North Carolina at Chapel Hill, Carolina Population Center [CPC], MEASURE Evaluation, 2006 Oct., 2006:[78] p.

11. Ogundipe O, Hoyo C, Ostbye T, et al. Factors associated with prenatal folic acid and iron supplementation among 21,889 pregnant women in Northern Tanzania: a cross-sectional hospital-based study. *BMC public health* 2012;12:481. doi: 10.1186/1471-2458-12-481 [published Online First: 2012/06/28]

12. Bloom S. Violence against Women and Girls: A Compendium of Monitoring and Evaluation Indicators. Chapel Hill, North Carolina, USA: MEASURE Evaluation, 2008.

13. Bunn MY, Higa NA, Parker WJ, et al. Domestic violence screening in pregnancy. *Hawaii medical journal* 2009;68(10):240-2. [published Online First: 2009/12/17]

14. Reeve C, Banfield S, Thomas A, et al. Community outreach midwifery-led model improves antenatal access in a disadvantaged population. *The Australian journal of rural health* 2016;24(3):200-6. doi: 10.1111/ajr.12249 [published Online First: 2015/09/24]

15. Chabikuli ON, Gwarzo U, Olufunso A, et al. Closing the prevention of mother-to-child transmission gap in Nigeria: an evaluation of service improvement intervention in Nigeria. *South African Family Practice* 2013;55(1):96-102.

16. Dako-Gyeke P, Dornoo B, Ayisi Addo S, et al. Towards elimination of mother-to-child transmission of HIV in Ghana: an analysis of national programme data. *International journal for equity in health* 2016;15:5. doi: 10.1186/s12939-016-0300-5 [published Online First: 2016/01/14]

17. Draper B, Abdullah F. A review of the prevention of mother-to-child transmission programme of the Western Cape provincial government, 2003 - 2004. *South African Medical Journal* 2008;98(6):431-4.

18. Herce ME, Mtande T, Chimbwandira F, et al. Supporting Option B+ scale up and strengthening the prevention of mother-to-child transmission cascade in central Malawi: results from a serial cross-sectional study. *BMC infectious diseases* 2015;15:328. doi: 10.1186/s12879-015-1065-y [published Online First: 2015/08/13]

19. Mukherjee S, Ghosh S, Goswami DN, et al. Performance evaluation of PPTCT (Prevention of parent to child transmission of HIV) programme: an experience from West Bengal. *The Indian journal of medical research* 2012;136(6):1011-9. [published Online First: 2013/02/09]

20. Delvaux T, Samreth S, Barr-DiChiara M, et al. Linked response for prevention, care, and treatment of HIV/AIDS, STIs, and reproductive health issues: results after 18 months of implementation in five operational districts in Cambodia. *Journal of acquired immune deficiency syndromes (1999)* 2011;57(3):e47-55. doi: 10.1097/QAI.0b013e318216d5cf [published Online First: 2011/03/17]

21. Bali K, Wheeler R, Davoodnia R, et al. Partnership for Maternal and Neonatal Health - West Pokot District Child Survival and Health Program. New York: Doctors of the World, 2007.

22. Global AIDS Response. Global AIDS Response Progress Reporting 2016. Geneva: UNAIDS, 2016.

23. Baker U, Okuga M, Waiswa P, et al. Bottlenecks in the implementation of essential screening tests in antenatal care: Syphilis, HIV, and anemia testing in rural Tanzania and Uganda. *International journal of gynaecology and obstetrics: the official organ of the International Federation of Gynaecology and Obstetrics* 2015;130 Suppl 1:S43-50. doi: 10.1016/j.ijgo.2015.04.017 [published Online First: 2015/06/10]

24. WHO. Global Reference List of 100 Core Health Indicators. Geneva: World Health Organization, 2015.

25. WHO. Consolidated Strategic Information Guidelines for HIV in the Health Sector. Geneva: World Health Organization, 2015a.

26. Hossain M, Broutet N, Hawkes S. The elimination of congenital syphilis: A comparison of the proposed world health organization action plan for the elimination of congenital syphilis with existing national maternal and congenital syphilis policies. *Sexually Transmitted Diseases* 2007;34(7):S22-S30. doi: 10.1097/olq.0000261049.84824.40

27. Olagbuji BN, Igbarumah S, Akintayo AA, et al. Maternal understanding of fetal movement in third trimester: a means for fetal monitoring and reducing stillbirth. *Nigerian journal of clinical practice* 2014;17(4):489-94. doi: 10.4103/1119-3077.134049 [published Online First: 2014/06/10]

28. Peat AM, Stacey T, Cronin R, et al. Maternal knowledge of fetal movements in late pregnancy. *The Australian & New Zealand journal of obstetrics & gynaecology* 2012;52(5):445-9. doi: 10.1111/j.1479-828X.2012.01462.x [published Online First: 2012/07/04]

29. Bookallil M, Chalmers E, Andrew B. Challenges in preventing pyelonephritis in pregnant women in Indigenous communities. *Rural and remote health* 2005;5(3):395. [published Online First: 2005/08/09]

30. Countdown to 2015. Building a Future for Women and Children: The 2012 Report. Washington, D.C.: World Health Organization and UNICEF, 2012.

31. Teferi E, Alemu H, Bulto T, et al. A descriptive study of the changes in coverage of preventive and promotive interventions before and after the introduction of integrated community case management (ICCM) in Ethiopia. *Ethiopian medical journal* 2014;52 Suppl 3:151-5. [published Online First: 2015/04/08]

32. MEASURE Evaluation, MEASURE DHS, President's Malaria Initiative, et al. Household Survey Indicators for Malaria Control. Chapel Hill, North Carolina, USA: MEASURE Evaluation, 2013.

33. WHO. Malaria in pregnancy: Guidelines for measuring key monitoring and evaluation indicators. Geneva: WHO, 2007:[38] p.

34. Economic Growth Center (EGC) at Yale University, Institute of Statistical SERIaUoG, . Ghana Socio-economic Panel Survey: Household Instrument Wave One (2009) Part B. Living Standards Measurement Study Questionnaire. Legon, Ghana: University of Ghana, 2009.

35. Beeckman K, Frith L, Gottfreosdottir H, et al. Measuring antenatal care use in Europe: is the content and timing of care in pregnancy tool applicable? *International Journal of Public Health* 2017;62(5):583-90. doi: 10.1007/s00038-017-0959-4

36. Marchant T, Tilley-Gyado RD, Tessema T, et al. Adding Content to Contacts: Measurement of High Quality Contacts for Maternal and Newborn Health in Ethiopia, North East Nigeria, and Uttar Pradesh, India. *PloS one* 2015;10(5) doi: 10.1371/journal.pone.0126840

37. Melo EC, de Oliveira RR, Mathias TAD. Factors associated with the quality of prenatal care: an approach to premature birth. *Revista Da Escola De Enfermagem Da Usp* 2015;49(4):540-48. doi: 10.1590/s0080-623420150000400002

38. Wilunda C, Tanaka S, Putoto G, et al. Evaluation of a maternal health care project in South West Shoa Zone, Ethiopia: before-and-after comparison. *Reproductive health* 2016;13(1):95. doi: 10.1186/s12978-016-0213-1 [published Online First: 2016/08/21]

39. Ejigu T, Woldie M, Kifle Y. Quality of antenatal care services at public health facilities of Bahir-Dar special zone, Northwest Ethiopia. *BMC health services research* 2013;13:443. doi: 10.1186/1472-6963-13-443 [published Online First: 2013/10/29]

40. McNabb M, Chukwu E, Ojo O, et al. Assessment of the quality of antenatal care services provided by health workers using a mobile phone decision support application in northern Nigeria: a pre/post-intervention study. *PloS one* 2015;10(5):e0123940. doi: 10.1371/journal.pone.0123940 [published Online First: 2015/05/06]

41. Mbuagbaw L, Medley N, Darzi AJ, et al. Health system and community level interventions for improving antenatal care coverage and health outcomes. *The Cochrane database of systematic reviews* 2015(12):Cd010994. doi: 10.1002/14651858.CD010994.pub2 [published Online First: 2015/12/02]

42. Rumbold AR, Bailie RS, Si D, et al. Delivery of maternal health care in Indigenous primary care services: baseline data for an ongoing quality improvement initiative. *BMC pregnancy and childbirth* 2011;11:16. doi: 10.1186/1471-2393-11-16 [published Online First: 2011/03/10]

43. National Institute for Health and Care Excellence. NICE menu indicator guidance: Indicator CCG30. London: NICE, 2016.

44. Yucel C, Taskin L, Low LK. Validity and reliability of the Turkish version of the Optimality Index-US (OI-US) to assess maternity care outcomes. *Midwifery* 2015;31(12):1135-42. doi: 10.1016/j.midw.2015.08.003 [published Online First: 2015/08/25]

45. Servan-Mori E, Contreras-Loya D, Gomez-Dantes O, et al. Use of performance metrics for the measurement of universal coverage for maternal care in Mexico. *Health Policy and Planning* 2017;32(5):625-33. doi: 10.1093/heapol/czw161

46. Kildea S, Gao Y, Rolfe M, et al. Remote links: Redesigning maternity care for Aboriginal women from remote communities in Northern Australia - A comparative cohort study. *Midwifery* 2016;34:47-57. doi: 10.1016/j.midw.2016.01.009 [published Online First: 2016/03/15]

47. Liu L, Li M, Yang L, et al. Measuring coverage in MNCH: a validation study linking population survey derived coverage to maternal, newborn, and child health care records in rural China. *PloS one* 2013;8(5):e60762. doi: 10.1371/journal.pone.0060762 [published Online First: 2013/05/15]

48. Lu J, Shen JJ, Chen G, et al. Regional disparities in prenatal care services in rural China. *Asia-Pacific journal of public health* 2011;23(5):682-9. doi: 10.1177/1010539511418356 [published Online First: 2011/08/20]

49. Doubova SV, Perez-Cuevas R, Ortiz-Panozo E, et al. Evaluation of the quality of antenatal care using electronic health record information in family medicine clinics of Mexico City. *BMC pregnancy and childbirth* 2014;14:168. doi: 10.1186/1471-2393-14-168 [published Online First: 2014/06/03]

50. McPherson RA, Khadka N, Moore JM, et al. Are birth-preparedness programmes effective? Results from a field trial in Siraha district, Nepal. *Journal of health, population, and nutrition* 2006;24(4):479-88. [published Online First: 2007/06/27]

51. MICS. Questionnaire for Individual Women. MICS6 Questionnaires. New York: UNICEF, 2017.

52. The DHS Program. Measure DHS service provision assessment survey: Inventory questionnaire. SPA Questionnaires. Rockville, Maryland, USA: ICF International, 2012.

53. The DHS Program. Measure DHS service provision assessment survey: Observation of ANC consultation. SPA Questionnaires. Rockville, Maryland, USA: ICF International, 2012.

54. The DHS Program. Measure DHS service provision assessment survey: Health worker interview. SPA Questionnaires. Rockville, Maryland, USA: ICF International, 2012.

55. The DHS Program. Measuring service availability and readiness: A health facility assessment methodology for monitoring health system strengthening. Service Readiness Indicators. Geneva: WHO and USAID, 2012.

56. The DHS Program. Malaria Indicator Survey Model Woman's Questionnaire. Malaria Surveys. Rockville, Maryland, USA: The DHS Program, 2016.

57. The DHS Program. Measure DHS service provision assessment survey: ANC client exit interview. SPA Questionnaires. Rockville, Maryland, USA: ICF International, 2012.

58. The DHS Program. Model Woman's Questionnaire. DHS Questionnaires and Manuals - Phase 7. Rockville, Maryland, USA: ICF International, 2017.
