# Supplementary material for: Developing measures for WHO recommendations on antenatal care for a positive pregnancy experience: a conceptual framework and scoping review
Source: BMJ Open. 2019 Apr 24;9(4):e024130. doi: 10.1136/bmjopen-2018-024130 (PMC6502222; doi:10.1136/bmjopen-2018-024130)
Supplement: Supplementary file 2 [file bmjopen-2018-024130supp002.pdf]

Table A2: ANC recommendations and established good clinical practices without existing measures

Domain	WHO ANC Recommendation or Established Good Clinical Practice
Content of Care	A.1.1 - Counselling about healthy eating and keeping physically active during pregnancy is recommended for pregnant women to stay healthy and to prevent excessive weight gain during pregnancy
Content of Care	A.1.2 - In undernourished populations, nutrition education on increasing daily energy and protein intake is recommended for pregnant women to reduce the risk of low-birth-weight neonates
Content of Care	A.1.3 - In undernourished populations, balanced energy and protein dietary supplementation is recommended for pregnant women to reduce the risk of stillbirths and small-for-gestational-age neonates
Content of Care	A.3 - In populations with low dietary calcium intake, daily calcium supplementation (1.5–2.0 g oral elemental calcium) is recommended for pregnant women to reduce the risk of pre-eclampsia
Content of Care	A.4 - Vitamin A supplementation is only recommended for pregnant women in areas where vitamin A deficiency is a severe public health problem to prevent night blindness
Content of Care	A.5 - Zinc supplementation for pregnant women is only recommended in the context of rigorous research
Content of Care	A.10 - For pregnant women with high daily caffeine intake (more than 300 mg per day), lowering daily caffeine intake during pregnancy is recommended to reduce the risk of pregnancy loss and low-birth-weight neonates
Content of Care	B.1.1 - Full blood count testing is the recommended method for diagnosing anaemia in pregnancy. In settings where full blood count testing is not available, on-site haemoglobin testing with a haemoglobinometer is recommended over the use of the haemoglobin colour scale as the method for diagnosing anaemia in pregnancy
Content of Care	B.1.2 - Midstream urine culture is the recommended method for diagnosing asymptomatic bacteriuria (ASB) in pregnancy. In settings where urine culture is not available, on-site midstream urine Gramstaining is recommended over the use of dipstick tests as the method for diagnosing ASB in pregnancy
Content of Care	B.1.4 - Hyperglycaemia first detected at any time during pregnancy should be classified as either gestational diabetes mellitus (GDM) or diabetes mellitus in pregnancy, according to WHO criteria
Content of Care	B.1.6 - Health-care providers should ask all pregnant women about their use of alcohol and other substances (past and present) as early as possible in the pregnancy and at every antenatal care visit
Content of Care	B.1.8 - In settings where the tuberculosis (TB) prevalence in the general population is 100/100 000 population or higher, systematic screening for active TB should be considered for pregnant women as part of antenatal care
Content of Care	B.2.4 - One ultrasound scan before 24 weeks of gestation (early ultrasound) is recommended for pregnant women to estimate gestational age, improve detection of fetal anomalies and multiple pregnancies, reduce induction of labour for post-term pregnancy, and improve a woman's pregnancy experience
Content of Care	C.2 - Antibiotic prophylaxis is only recommended to prevent recurrent urinary tract infections in pregnant women in the context of rigorous research

Content of Care	C.3 - Antenatal prophylaxis with anti-D immunoglobulin in non-sensitized Rh-negative pregnant women at 28 and 34 weeks of gestation to prevent RhD alloimmunization is only recommended in the context of rigorous research
Experience of Care	D.1 - Ginger, chamomile, vitamin B6 and/or acupuncture are recommended for the relief of nausea in early pregnancy, based on a woman's preferences and available options
Experience of Care	D.2 - Advice on diet and lifestyle is recommended to prevent and relieve heartburn in pregnancy. Antacid preparations can be offered to women with troublesome symptoms that are not relieved by lifestyle modification
Experience of Care	D.3 - Magnesium, calcium or non-pharmacological treatment options can be used for the relief of leg cramps in pregnancy, based on a woman's preferences and available options
Experience of Care	D.4 - Regular exercise throughout pregnancy is recommended to prevent low back and pelvic pain. There are a number of different treatment options that can be used, such as physiotherapy, support belts and acupuncture, based on a woman's preferences and available options
Experience of Care	D.5 - Wheat bran or other fibre supplements can be used to relieve constipation in pregnancy if the condition fails to respond to dietary modification, based on a woman's preferences and available options
Experience of Care	D.6 - Non-pharmacological options, such as compression stockings, leg elevation and water immersion, can be used for the management of varicose veins and oedema in pregnancy, based on a woman's preferences and available options
Health Systems	E.2 - Midwife-led continuity-of-care models, in which a known midwife or small group of known midwives supports a woman throughout the antenatal, intrapartum and postnatal continuum, are recommended for pregnant women in settings with well functioning midwifery programmes
Health Systems	E.3 - Group antenatal care provided by qualified health-care professionals may be offered as an alternative to individual antenatal care for pregnant women in the context of rigorous research, depending on a woman's preferences and provided that the infrastructure and resources for delivery of group antenatal care are available
Health Systems	E.4.1 - The implementation of community mobilization through facilitated participatory learning and action (PLA) cycles with women's groups is recommended to improve maternal and newborn health, particularly in rural settings with low access to health services. Participatory women's groups represent an opportunity for women to discuss their needs during pregnancy, including barriers to reaching care, and to increase support to pregnant women
Health Systems	E.4.2 - Packages of interventions that include household and community mobilization and antenatal home visits are recommended to improve antenatal care utilization and perinatal health outcomes, particularly in rural settings with low access to health services
Health Systems	E.5.1 - Task shifting the promotion of health-related behaviours for maternal and newborn health to a broad range of cadres, including lay health workers, auxiliary nurses, nurses, midwives and doctors is recommended
Health Systems	E.5.2 - Task shifting the distribution of recommended nutritional supplements and intermittent preventive treatment in pregnancy (IPTp) for malaria prevention to a broad range of cadres, including auxiliary nurses, nurses, midwives and doctors is recommended
Content of Care	Fetal heart rate monitored †

† Established good clinical practice within ANC
