# Supplementary material for: Developing measures for WHO recommendations on antenatal care for a positive pregnancy experience: a conceptual framework and scoping review
Source: BMJ Open. 2019 Apr 24;9(4):e024130. doi: 10.1136/bmjopen-2018-024130 (PMC6502222; doi:10.1136/bmjopen-2018-024130)
Supplement: Author's manuscript [file bmjopen-2018-024130.draft_revisions.pdf]

BMJ Open is committed to open peer review. As part of this commitment we make the peer review history of every article we publish publicly available.

When an article is published we post the peer reviewers' comments and the authors' responses online. We also post the versions of the paper that were used during peer review. These are the versions that the peer review comments apply to.

The versions of the paper that follow are the versions that were submitted during the peer review process. They are not the versions of record or the final published versions. They should not be cited or distributed as the published version of this manuscript.

BMJ Open is an open access journal and the full, final, typeset and author-corrected version of record of the manuscript is available on our site with no access controls, subscription charges or pay-per-view fees (http://bmjopen.bmj.com).

If you have any questions on BMJ Open's open peer review process please email editorial.bmjopen@bmj.com

BMJ Open

# Developing measures for WHO recommendations on antenatal care for a positive pregnancy experience: a scoping review and conceptual framework


| | |
|---|---|
| Journal: | *BMJ Open* |
| Manuscript ID | bmjopen-2018-024130 |
| Article Type: | Research |
| Date Submitted by the Author: | 11-May-2018 |



| | |
|---|---|
| Complete List of Authors: | Lattof, Samantha; World Health Organization, Department of Reproductive Health and Research<br>Tunçalp, Özge; World Health Organizations, Reproductive Health and Research<br>Moran, A; World Health Organization, Department of Maternal, Newborn, Child and Adolescent Health<br>Bucagu, Maurice; WHO, Department of Maternal, Newborn, Child and Adolescent Health<br>Chou , D ; World Health Organization, Department of Reproductive Health and Research<br>Diaz, Theresa; World Health Organizations, Department of Maternal, Newborn, Child and Adolescent Health<br>Gülmezoglu, A; World Health Organization, Department of Reproductive Health and Research |



| | |
|---|---|
| Keywords: | Protocols & guidelines < HEALTH SERVICES ADMINISTRATION & MANAGEMENT, Quality in health care < HEALTH SERVICES ADMINISTRATION & MANAGEMENT, OBSTETRICS, PUBLIC HEALTH |


SCHOLARONE™
Manuscripts

# Title

Developing measures for WHO recommendations on antenatal care for a positive pregnancy experience: a scoping review and conceptual framework

# Authors

Corresponding author:

Samantha R. Lattof, PhD; Department of Reproductive Health and Research including UNDP/UNFPA/UNICEF/WHO/World Bank Special Programme of Research, Development and Research Training in Human Reproduction (HRP), World Health Organization, Avenue Appia 20, 1202 Geneva, Switzerland; lattof@post.harvard.edu, tel. +49 171 7549561

Co-authors:

Özge Tunçalp, MD; Department of Reproductive Health and Research including UNDP/UNFPA/UNICEF/WHO/World Bank Special Programme of Research, Development and Research Training in Human Reproduction (HRP), World Health Organization, Geneva, Switzerland

Allisyn C. Moran, PhD; Scientist, Department of Maternal, Newborn, Child and Adolescent Health, World Health Organization, Geneva, Switzerland

Maurice Bucagu, PhD; Medical Officer, Department of Maternal, Newborn, Child and Adolescent Health, World Health Organization, Geneva, Switzerland

Doris Chou, MD; Medical Officer, Department of Reproductive Health and Research including UNDP/UNFPA/UNICEF/WHO/World Bank Special Programme of Research, Development and Research Training in Human Reproduction (HRP), World Health Organization, Geneva, Switzerland

Theresa Diaz, MD; Coordinator, Department of Maternal, Newborn, Child and Adolescent Health, World Health Organization, Geneva, Switzerland

A. Metin Gülmezoglu, MD; Coordinator, Department of Reproductive Health and Research including UNDP/UNFPA/UNICEF/WHO/World Bank Special Programme of Research, Development and Research Training in Human Reproduction (HRP), World Health Organization, Geneva, Switzerland

# Keywords

prenatal care; pregnancy; guideline; quality indicators; public health

# Word count

3,789 words

For peer review only

# Abstract

*Objectives*

In response to the newest World Health Organization (WHO) recommendations on routine antenatal care (ANC) for pregnant women and adolescent girls, this paper identifies the literature on existing ANC measures, presents a conceptual framework for quality ANC, maps existing measures to specific WHO recommendations, identifies gaps where new measures are needed to monitor the implementation and impact of routine ANC, and prioritizes measures for capture.

*Methods*

We conducted searches in four databases and five websites. Searches and application of inclusion/exclusion criteria followed the PRISMA flow approach. Data were extracted on measure information, methodology, methodological work, and implementation. We inductively developed and defined a conceptual framework for routine ANC based on these measures.

*Results*

This scoping review uncovered 46 existing measures that align with the guideline. Only 14/42 interventions and three established good clinical practices could be measured immediately using existing measures. Recommendations addressing the integration of ANC with allied fields are more likely to have existing measures than recommendations that focus on maternal health. When mapped to our conceptual framework, existing measures prioritize content of care and health systems; measures for girls' and women's experiences of care are notably lacking. Existing data sources for non-existent measures are currently limited.

*Conclusion*

Our research updates prior efforts to develop comprehensive measures of quality ANC and raises awareness of the need to better assess women's experiences of care. Given the inadequate number and distribution of existing ANC measures across the quality of care conceptual framework domains, new standardized measures are required to assess quality of routine ANC. Girls' and women's voices deserve greater acknowledgment when measuring the quality and delivery of ANC.


For peer review only

## Strengths and limitations of this study

- This research updates prior efforts to develop comprehensive measures of quality antenatal care and raises awareness of the need to better assess women's experiences of care.
- We searched a range of databases and websites with different disciplinary foci and solicited expert recommendations to maximize heterogeneity in the material included in this scoping review.
- To minimize bias in the scoping review and to generate reliable findings from which to draw conclusions about substantive measures for routine antenatal care, we employed the PRISMA flow approach.
- Methodological limitations may have resulted in missed relevant indicators, specifically relevant indicators in languages other than English, as well as indicators in grey literature and in-service reports, where it was unfeasible to search these infinite sources thoroughly.

For peer review only

## Introduction

In 2016, the World Health Organization (WHO) released its comprehensive recommendations on routine antenatal care (ANC) for pregnant women and adolescent girls.[1] A systematic review of women's views shaped the scope of this guideline, since women's experiences of ANC are key to transforming ANC and to creating the foundation for healthy motherhood. At its core, this guideline aims "to provide pregnant women with respectful, individualized, person-centred care at every contact, with implementation of effective clinical practices (interventions and tests), and provision of relevant and timely information, and psychosocial and emotional support, by practitioners with good clinical and interpersonal skills within a well functioning health system," (p. 105-06).[1] Monitoring the implementation and impact of routine ANC, as described in the guideline, requires moving beyond the global benchmark indicator of four or more ANC visits to include measures focusing on ANC content and care processes.[2]

The WHO has prioritized the development of indicators in recent years, releasing guidelines and measures for core health,[3] essential interventions,[4] preventable maternal mortality,[5] preventable newborn deaths and stillbirths,[6] and quality of maternal and newborn care in health facilities;[7] however, limited ANC measures appear throughout these documents. Other efforts to develop comprehensive measures of quality ANC have resulted in a narrow range of measures that emphasize clinical practices like hospitalization for uncontrolled diabetes[8] or indicators for service utilization, screening, and interventions.[9] Measuring women and girls' experience of respectful, individualized, person-centred ANC; the provision of relevant and timely information; and psychosocial and emotional support have received far less attention.

Variation in data quality, data (in)availability, and data heterogeneity (e.g. data sources, types of data, data uses) also challenge existing efforts to assess ANC coverage, content, and quality.[10] In recognition of these challenges, researchers have called for the collection of high-quality ANC data,[11] the measurement of content of care as well as the number of contacts,[2 5] the establishment of relevant indicators to support data collection for developing appropriate policy for vulnerable populations,[12] and greater support for the implementation of digital registries and data platforms that overcome gaps in the availability of existing information from publicly available household and facility surveys.[4] Country programmes have also called for better data so that decision-makers and advocates have the necessary evidence to improve maternal and newborn survival and health.[13]

With the launch of the new guideline and as countries reassess their ANC programmes accordingly, there is now an urgent need to develop and implement appropriate measures for national and global monitoring of routine ANC. This paper presents a scoping review that aims to (1) identify the literature on ANC measures as they relate to the WHO guideline[1] and (2) map existing measures to specific recommendations in the guideline. To inform the development of a monitoring framework for implementation of the WHO ANC model and ground the model in a conceptual framework for quality ANC, our paper also identifies gaps where new measures are needed for implementation and prioritizes measures for capture.

For peer review only

# Methods

## Conceptual framework

Within the context of maternal and newborn health, the WHO defines quality of care as "*the degree to which maternal and newborn health services (for individuals and populations) increase the likelihood of timely, appropriate care for the purpose of achieving desired outcomes that are both consistent with current professional knowledge and take into account the preferences and aspirations of individual women and their families,*" (p. 15).[7] Based on the guideline supplemented by the findings from the scoping review, we customized the WHO's framework for quality maternal and newborn health care[7] focusing on routine ANC. We designed the framework by grouping similar measures for assessing, improving, and monitoring ANC. Existing and non-existent ANC measures are mapped by recommendation to the framework's domains; thus, the framework only includes health system building blocks that align with the new ANC recommendations.

## Search strategy and screening process

After first testing search terms that have been identified as inappropriate for this scoping review (e.g. measure*), we conducted searches of the final search terms ((antenatal OR prenatal) AND (indicator*)) in four databases (PubMed, ISI Web of Science, ScienceDirect, Popline), according to the following specifications. The search included pregnant women and girls and any measure matching one of the recommendations in the publication *WHO recommendations on antenatal care for a positive pregnancy experience* or one of the four specified practices of good clinical practice during ANC, published since 1 January 2005. Searches were constrained by species (human) and language (English) when databases permitted and adapted to the particulars (e.g. wildcards, truncations, capacity for complex searches) of each electronic database. Searches in ScienceDirect were limited to title, abstract, and keywords. In addition, we thematically searched five additional websites (WHO, MEASURE Evaluation, The Demographic and Health Survey (DHS) Program, UNICEF Multiple Indicator Cluster Surveys (MICS), Countdown to 2030) for webpages related to indicators and/or to maternal health, in order to maximize the coverage of non-peer-reviewed materials.

SL conducted the searches and subsequent application of inclusion/exclusion criteria according to the PRISMA (Preferred Reporting Items for Systematic Reviews and Meta-Analyses) flow approach. SL and OT independently screened the first 50 items, compared results, and discussed and resolved any differences in understanding of the inclusion/exclusion criteria. The criteria were further elaborated where necessary on the basis of this independent screening. SL screened items identified by the search on the basis of title and abstract (TIAB). When inclusion or exclusion could not be determined on the basis of TIAB, full texts were screened. At the full text screening stage, two reviewers independently screened any items that SL considered borderline or problematic.

## Inclusion and exclusion criteria

This review focuses on measures of ANC for a positive pregnancy experience based on the WHO's most recent guideline.[1] As part of the guideline development process, the Guideline Development Group did not evaluate evidence or make recommendations for good clinical practices, as they are considered to be essential components of ANC.[1] Since these established

For peer review only

good clinical practices should be implemented alongside the guideline's recommendations and are part of the WHO ANC model, this scoping review also considers measures for four established practices of good ANC for inclusion. These established good clinical practices include: counselling on birth preparedness, counselling on family planning, monitoring of fetal heart rate, and monitoring of blood pressure.[1]

Resources on pregnant girls and women in any setting were considered. Peer-reviewed journal articles and (non-peer-reviewed) grey literature were eligible for inclusion. Potentially eligible resources included journal articles and un/published information from governments and other agencies, whether available in print or online, published in English. Quality was not assessed and was not a criterion for inclusion. Multiple references based on the same measure/recommendation were not excluded, as they might contain useful information on current methodological work and experiences implementing the measure.

For inclusion, resources must have:
- Used a measure that aligns with at least one of the recommendations in the publication *WHO recommendations on antenatal care for a positive pregnancy experience*[1] or one of the four specified clinical practices of good ANC
- Defined the measure
- Explained the methodology for measurement
- Be published in English
- Be published between 1 January 2005 and 26 August 2017

Unless resources conducting secondary analyses of popular global datasets (e.g. MICS, DHS, DHS Service Provision Assessments) introduced new measures or indices based on the data, these articles were excluded in favour of primary resources (e.g. DHS questionnaire, MICS questionnaire). If resources specified a measure's source as a common global measure (e.g. from Countdown to 2015, from the Millennium Development Goals), then these resources were excluded in favour of primary resources (e.g. Countdown to 2015 report[14]). This scoping review excludes the guideline's seven recommendations for which the Guideline Development Group recommended no intervention (A.1.4, A.6, A.7, A.8, A.9, B.2.3, and B.2.5).[1]

**Data extraction and analysis**

Since the focus of this research is on specific measures rather than specific studies, individual measure data were compiled from multiple studies. Data were extracted on measure information (e.g. definition, numerator, denominator), methodology (e.g. method of measurement, measurement frequency, data sources), published or current methodological work, and experiences implementing the measure. We inductively developed and defined a conceptual framework for routine ANC based on these measures. Results are presented in a narrative format accompanied by tables of identified measurement areas for ANC guideline monitoring and evaluation.

**Patient and Public Involvement**

This study did not involve patients.

For peer review only

# Results

### WHO quality of care framework focusing on routine antenatal care

Building on the WHO framework for quality maternal and newborn health care,[7] we mapped the existing and non-existent ANC measures across three dimensions by which to measure recommendations for routine ANC (Figure 1): health systems (existing measures = 7, non-existent measures = 6), content of care (existing measures = 39, non-existent measures = 16), and women's experience of care (existing measures = 0, non-existent measures = 6). These three domains form the core of the conceptual framework and influence outcomes at the individual and facility levels. This framework is intended to assist health care providers, managers, and policymakers to better understand and improve the quality of routine ANC for a positive pregnancy experience. It can also be used to assess health system characteristics required to deliver quality ANC.

The health system provides the structure by which factors such as service delivery models and health workforce organization impact quality of ANC processes. The content of ANC and women's experiences of ANC form the two pillars of quality ANC and are reliant upon the availability of human resources and physical resources. Content of care includes interventions related to maternal and fetal assessment and management, nutritional interventions, infectious disease testing and management, and counselling and information sharing. As part of the WHO quality of care framework, women's experience of care includes effective communication, respect and dignity and emotional support. When ANC recommendations were mapped to women's experience of care, assessment and management of physical symptoms were the only ones.

### Scoping review results

Figure 2 illustrates the screening process. The scoping generated a total of 58 resources that described 46 existing measures (Supplementary Material, Table A1). These measures align with 14 recommendations for a positive pregnancy experience and three established good clinical practices. Multiple measures exist for recommendations addressing iron and folic acid supplements (n=7), HIV and syphilis (n=7), tetanus toxoid vaccination (n=6), intermittent preventive treatment of malaria in pregnancy (IPTp) (n=4), intimate partner violence (n=2), and tobacco (n=2). Nine additional measures exist for three established good clinical practices within ANC: monitoring of blood pressure (n=5), counselling about birth preparedness (n=2), and counselling about family planning (n=2). Twenty-seven of the guideline's recommendations and one established good clinical practice lack existing measures (Supplementary Material, Table A2). Table 1 sorts these existing and missing measurement areas by topic. Proposed measurement areas for recommendations in the context of research are summarized in Table 2.

For peer review only

**Table 1: Identified measurement areas for ANC guideline monitoring and evaluation by topic areas**

| Topic Area | What to be measured? | Measure Details |
|---|---|---|
| **Health Systems** | | |
| Service delivery models | Antenatal care contacts (eight or more) | Currently being used |
| | Pregnant women carrying their own case notes | |
| | Service-specific availability and readiness: midwife-led continuity-of-care | Context-specific |
| | Timing of first antenatal care visit | Currently being used |
| Health workforce organization | Health worker density and distribution | Currently being used; context-specific |
| | Health units with at least one service provider trained to care for and refer sexual and gender-based violence survivors | Currently being used; context-specific |
| | Policy on task shifting for antenatal care (counselling and provision of selected interventions) | |
| Community engagement | Communities offering facilitated participatory learning and action cycles with women's groups to improve maternal and newborn health | Context-specific |
| Maternal and fetal assessment and management | Assessment for tobacco use and second-hand smoke exposure | Currently being used |
| | Assessment for use of alcohol and other substances | |
| | Ultrasound scan before 24 weeks | |
| | On-site haemoglobin testing for anaemia | Context-specific |
| | On-site testing for asymptomatic bacteriuria | Context-specific |
| | Treatment for asymptomatic bacteriuria | |
| | Symphysis-fundal height measurement | Context-specific |
| | Monitoring of fetal heart rate | Established good clinical practice within ANC |
| | Monitoring of blood pressure | Currently being used; established good clinical practice within ANC |
| Nutritional interventions | Iron and folic acid supplementation | Currently being used; context-specific |
| | Availability of balanced energy and protein dietary supplementation | |
| | Calcium supplementation | Context-specific |
| | Vitamin A supplementation coverage | Context-specific |
| | Caffeine intake information | |

For peer review only

| Topic Area | What to be measured? | Measure Details |
|---|---|---|
| Infectious disease testing and management | Pregnant women counselled and tested for HIV and know their results | Currently being used |
| | Testing for syphilis | Currently being used |
| | Treatment for helminths | Currently being used; context-specific |
| | Newborns protected at birth from tetanus | Currently being used |
| | Intermittent preventive therapy for malaria | Currently being used; context-specific |
| | Testing for tuberculosis | Context-specific |
| | Antiretroviral pre-exposure prophylaxis to prevent HIV infection | Context-specific |
| Counselling and information sharing | Counselling on diet and exercise in pregnancy | Context-specific |
| | Counselling on birth preparedness | Currently being used; established good clinical practice within ANC |
| | Counselling on family planning | Currently being used; established good clinical practice within ANC |
| Management of physiological symptoms | Information and treatment for common physiological symptoms (e.g. leg cramps, constipation, nausea) | |

Note: This table condenses measures by intervention type. For example, "iron and folic acid supplementation" includes all measures for recommendations A.2.1 (daily oral iron and folic acid supplementation) and A.2.2 (intermittent oral iron and folic acid supplementation).

**Table 2: Identified measurement areas for monitoring ANC recommendations in the context of research by topic areas**

| Topic Area | What to be measured? | Measure Details |
|---|---|---|
| **Health Systems** | | |
| Service delivery models | Service-specific availability and readiness: group antenatal care | |
| **Content of Care** | | |
| Maternal and fetal assessment and management | Prophylaxis for recurrent urinary tract infections | |
| | Prophylaxis with anti-D immunoglobulin in non-sensitized Rh-negative pregnant women | |
| | Daily fetal movement counting | Currently being used |
| | Zinc supplementation | |

For peer review only

Existing measures are primarily implemented within the content of care domain followed by the health systems domain. Existing measures for women's experience of ANC in terms of communication, support and respect are sparse and infrequently collected; our scoping review revealed no existing measures for recommendations involving women's experiences of care. The existing measures could permit immediate measurement of 14 ANC recommendations in the WHO guideline using currently available data. Three of the 14 recommendations (B.1.7, C.6, and E.7)[1] are perfectly aligned with existing measures. The 11 remaining recommendations have subtle gaps or discrepancies with the existing measures and would require minimal modification or disaggregation to be relevant. Furthermore, some of the measures that are currently used may need to be revised based on the new recommendations (e.g. number of ANC contacts).

Most measures currently being implemented have consistent definitions; however, ten measures exhibit variation in their definitions and measurement (i.e. numerator and denominator). The measure "iron and folic acid supplements for pregnant women (%)," for instance, has multiple definitions that vary by supplementation duration (unspecified, 90+ days, at least six months), how women obtained the supplements (received, received or bought, given), and supplement dosage (unspecified, any, amounts in accordance with recommended protocols).[4 15-17] The measure "first antenatal care visit in the first trimester" presents even greater variation in measurement with researchers defining the first trimester as: first trimester,[18 19] <12 weeks,[20 21] ≤12 weeks,[22] <13 weeks,[23] by 13 weeks 0 days,[24] and <14 weeks.[25]

Among the recommendations lacking existing measures (Supplementary Material, Table A2), these non-existent measures relate to interventions involving health systems (n=6), nutrition (n=7), maternal and fetal assessment (n=6), common physiological symptoms (n=6), preventative measures (n=2), and good clinical practice (n=1). Several proposed measures (Table 1) are for similar recommendations and could potentially be streamlined. For instance, recommendations E.5.1 and E.5.2 (task shifting components of ANC delivery) are quite similar; E.5.1 focuses on a broad range of activities whereas E.5.2 focuses solely on the distribution of nutritional supplements and IPTp for malaria prevention.[1] A single new health system measure "policy on task shifting for antenatal care" could collect data on both recommendations and then be disaggregated to permit monitoring the two recommendations separately.

Sources of existing data for these non-existent measures are currently limited. Monitoring the guideline's components of quality ANC that currently lack existing measures would thus require the development of new measures as well as improved data sources. Data for eight proposed measures could be obtained relatively quickly from women's individual ANC records (case notes), and health policy guidelines/directives. Data from existing clinical records could be difficult to obtain, as these data are often not linked for each ANC visit. Population-based surveys (e.g. DHS, MICS) do not currently capture the required data for these recommendations, necessitating the adaptation of existing tools to capture new measures. MICS, for example, currently asks whether women have a card or other document with their immunisations listed.[26] If this question (MN7) could be amended to include ANC case notes, it would permit the monitoring of recommendation E.1 (women-held case notes).[1]

# Discussion

This scoping review uncovered 46 existing measures that align with the WHO recommendations on routine ANC for pregnant women and adolescent girls.[1] Of the 42 recommended ANC interventions included in this scoping review, we found that only 14 WHO recommended interventions and three established good clinical practices could potentially be measured immediately using existing measures. Recommendations addressing the integration of ANC with allied fields (e.g. HIV, malaria, nutrition) are more likely to have existing measures than recommendations that focus solely on maternal health, especially recommendations related to organization of care (e.g. midwife-led continuity-of-care, group ANC). These existing measures prioritize content of care followed by health systems. Existing measures largely overlook girls' and women's experiences of care, the third dimension at the core of the quality of care framework for routine ANC. Research highlights that experience of care is an integral part of quality of care[27] and respectful care as described by women. In fact, experience of care overlaps with almost all of the quality of care domains described above.[28] Designing health care services with a women-centred approach is critical for utilization, quality, and impact of these services; therefore, girls' and women's voices deserve greater acknowledgment when measuring the quality and delivery of ANC.[29]

Like other researchers, we found that measures to assess ANC coverage and content are restricted by data heterogeneity, variation in data quality, and data availability.[4 10] Measures must be standardized in definition, measurement, and level of data collection and usage if they are to permit meaningful comparability across settings and over time. Monitoring the implementation and impact of routine ANC, as described in the WHO guideline,[1] is one of multiple efforts that would benefit from standardizing and strengthening the development of ANC measures. Moreover, improved measures would assist researchers and programme implementers in assessing the content and quality of ANC,[30] revealing ANC implementation bottlenecks,[31] assessing equity of ANC programme coverage and utilization,[32] and evaluating the effectiveness of new innovations like mHealth platforms for delivering maternal and child health services.[33]

Our research updates prior efforts to develop comprehensive measures of quality ANC,[8 9 34] and it raises awareness of the need to better assess women's experiences of care. New tools currently under development by the WHO-convened Maternal Morbidity Working Group recognize the importance of women's experiences in measuring maternal morbidity and improving the health care of pregnant women.[35 36] In addition to creating better tools to evaluate quality ANC as experienced by girls and women, researchers must also improve human capacity for correct, timely documentation of ANC data. Evidence from Brazil revealed a discrepancy between data from antenatal booklets and data recorded in the SISPRENATAL (System to Accompany the Prenatal and Birth Humanization Program) software.[37] Researchers attributed the discrepancy between data sources to the timing of data collection. Antenatal booklets, in which data is recorded during women's visits, produced more reliable data than the SISPRENATAL software, in which health unit employees who were uninvolved in women's visits completed worksheets after women's visits.[37] These findings reinforce the need for reliable, high-quality data sources to properly monitor and evaluate quality ANC.

Methodological limitations may have resulted in missed relevant indicators, specifically relevant indicators in languages other than English, as well as indicators in grey literature and in-service reports, where it was unfeasible to search these infinite sources thoroughly.

For peer review only

Finally, we were unable to locate the full text for nine items for screening. It is possible that these items may have been eligible for inclusion. To minimize bias in the scoping review and to generate reliable findings from which to draw conclusions about substantive measures for routine ANC, we employed the PRISMA flow approach. To mitigate possible publication bias, this search strategy incorporated non-peer-reviewed and unpublished papers and reports. We searched a range of databases and websites with different disciplinary foci and solicited expert recommendations to maximize heterogeneity in the material included in this scoping review.

Given the inadequate number and distribution of existing ANC measures across the quality of care framework domains, new standardized measures are required to assess quality of routine ANC. The WHO's new ANC model emphasizes integrated delivery of all the care women need during the antenatal period, so the measurement of ANC should also be integrated. Developing these measures will require strong partnerships and greater integration with allied fields so that the measures are implemented correctly. Our scoping review and conceptual framework will inform the monitoring framework for ANC that will incorporate a prioritized list of measures to better assist countries and health facilities in consistently monitoring and assessing progress towards improved ANC. Furthermore, partners in South Africa have been implementing the new ANC model at scale since April 2017. In collaboration with the South African Medical Research Council, WHO is analysing the effects of implementing this model on workload, detecting hypertension, and perinatal mortality with the associated set of indicators that will further inform the development of this monitoring and evaluation framework. This framework will determine the feasibility and utility of data collection as well as the indicators' place within the global monitoring efforts undertaken by initiatives such as Ending Preventable Maternal Mortality. Development and testing of new indicators, especially for critical areas such as experience of care, will be part of the proposed research agenda.

## Acknowledgements

We wish to thank Juan Pablo Peña-Rosas at the World Health Organization for his contributions to the antenatal guideline on which this work is based.

## Contributors

OT, ACM, and SRL conceived and planned the study with input from DC, TD, MB and AMG. SRL conducted the searches and subsequent application of inclusion/exclusion criteria. SRL and OT independently screened the first 50 items to ensure consistency in the inclusion/exclusion criteria. SRL input the data. OT, ACM, SRL, and MB contributed to the interpretation of the findings. SRL wrote the first draft of the manuscript, and all authors contributed to subsequent revisions.

## Funding

This work was supported by the UNDP-UNFPA-UNICEF-WHO-World Bank Special Programme of Research, Development and Research Training in Human Reproduction (HRP), a co-sponsored programme executed by the World Health Organization (WHO).

For peer review only

## Competing interests

We declare no competing interests.

## Data sharing statement

Data extraction workbook available upon request from the WHO.

## References


1. WHO. WHO recommendations on antenatal care for a positive pregnancy experience. Geneva: WHO, 2016:[152] p.

2. Hodgins S, D'Agostino A. The quality–coverage gap in antenatal care: toward better measurement of effective coverage. *Global Health, Science and Practice* 2014;2(2):173-81. doi: 10.9745/GHSP-D-13-00176

3. WHO. Global Reference List of 100 Core Health Indicators (plus health-related SDGs). Geneva: World Health Organization, 2018.

4. Flenady V, Wojcieszek AM, Fjeldheim I, et al. eRegistries: indicators for the WHO Essential Interventions for reproductive, maternal, newborn and child health. *Bmc Pregnancy and Childbirth* 2016;16 doi: 10.1186/s12884-016-1049-y

5. Moran AC, Jolivet RR, Chou D, et al. A common monitoring framework for ending preventable maternal mortality, 2015–2030: phase I of a multi-step process. *BMC Pregnancy and Childbirth* 2016;16(1):250. doi: 10.1186/s12884-016-1035-4

6. Moxon SG, Ruysen H, Kerber KJ, et al. Count every newborn; a measurement improvement roadmap for coverage data. *BMC pregnancy and childbirth* 2015;15 Suppl 2:S8. doi: 10.1186/1471-2393-15-s2-s8 [published Online First: 2015/09/24]

7. WHO. Standards for improving quality of maternal and newborn care in health facilities. Geneva: WHO, 2016.

8. Korst LM, Gregory KD, Lu MC, et al. A Framework for the Development of maternal quality of care indicators. *Maternal and child health journal* 2005;9(3):317-41. doi: 10.1007/s10995-005-0001-y [published Online First: 2005/09/15]

9. Bollini P, Quack-Lotscher K. Guidelines-based indicators to measure quality of antenatal care. *Journal of evaluation in clinical practice* 2013;19(6):1060-6. doi: 10.1111/jep.12027 [published Online First: 2013/03/27]

10. Moller A-B, Petzold M, Chou D, et al. Early antenatal care visit: a systematic analysis of regional and global levels and trends of coverage from 1990 to 2013. *The Lancet Global Health*;5(10):e977-e83. doi: 10.1016/S2214-109X(17)30325-X


For peer review only

11. Raynes-Greenow C. Gaps and challenges underpinning the first analysis of global coverage of early antenatal care. *The Lancet Global Health* 2017;5(10):e949-e50. doi: 10.1016/S2214-109X(17)30346-7

12. Steenkamp M, Bar-Zeev S, Rumbold A, et al. Pragmatic indicators for remote Aboriginal maternal and infant health care: why it matters and where to start. *Australian and New Zealand journal of public health* 2010;34 Suppl 1:S5-8. doi: 10.1111/j.1753-6405.2010.00545.x [published Online First: 2010/07/21]

13. Evidence for Action. MamaYe Evidence for Action Stories of Change: Selected Case Studies. London: E4A, 2015.

14. Countdown to 2015. Building a Future for Women and Children: The 2012 Report. Washington, D.C.: World Health Organization and UNICEF, 2012.

15. MEASURE Evaluation. Summary List of Indicators Chapel Hill, North Carolina, USA: MEASURE Evaluation; 2017 [Available from: https://www.measureevaluation.org/prh/rh_indicators/indicator-summary accessed 27 Aug 2017.

16. MONITOR. Maternal and Newborn Indicator Mapping. Geneva: World Health Organization, 2017.

17. Hodgins S, McPherson R, Suvedi BK, et al. Testing a scalable community-based approach to improve maternal and neonatal health in rural Nepal. *Journal of perinatology : official journal of the California Perinatal Association* 2010;30(6):388-95. doi: 10.1038/jp.2009.181 [published Online First: 2009/11/13]

18. Servan-Mori E, Contreras-Loya D, Gomez-Dantes O, et al. Use of performance metrics for the measurement of universal coverage for maternal care in Mexico. *Health Policy and Planning* 2017;32(5):625-33. doi: 10.1093/heapol/czw161

19. Mbuagbaw L, Medley N, Darzi AJ, et al. Health system and community level interventions for improving antenatal care coverage and health outcomes. *The Cochrane database of systematic reviews* 2015(12):Cd010994. doi: 10.1002/14651858.CD010994.pub2 [published Online First: 2015/12/02]

20. Liu L, Li M, Yang L, et al. Measuring coverage in MNCH: a validation study linking population survey derived coverage to maternal, newborn, and child health care records in rural China. *PloS one* 2013;8(5):e60762. doi: 10.1371/journal.pone.0060762 [published Online First: 2013/05/15]

21. Rumbold AR, Bailie RS, Si D, et al. Delivery of maternal health care in Indigenous primary care services: baseline data for an ongoing quality improvement initiative. *BMC pregnancy and childbirth* 2011;11:16. doi: 10.1186/1471-2393-11-16 [published Online First: 2011/03/10]

22. Lu J, Shen JJ, Chen G, et al. Regional disparities in prenatal care services in rural China. *Asia-Pacific journal of public health* 2011;23(5):682-9. doi: 10.1177/1010539511418356 [published Online First: 2011/08/20]

For peer review only

23. Kildea S, Gao Y, Rolfe M, et al. Remote links: Redesigning maternity care for Aboriginal women from remote communities in Northern Australia - A comparative cohort study. *Midwifery* 2016;34:47-57. doi: 10.1016/j.midw.2016.01.009 [published Online First: 2016/03/15]

24. National Institute for Health and Care Excellence. NICE menu indicator guidance: Indicator CCG30. London: NICE, 2016.

25. Yucel C, Taskin L, Low LK. Validity and reliability of the Turkish version of the Optimality Index-US (OI-US) to assess maternity care outcomes. *Midwifery* 2015;31(12):1135-42. doi: 10.1016/j.midw.2015.08.003 [published Online First: 2015/08/25]

26. MICS. Questionnaire for Individual Women. MICS6 Questionnaires. New York: UNICEF, 2017.

27. Bohren MA, Titiloye MA, Kyaddondo D, et al. Defining quality of care during childbirth from the perspectives of Nigerian and Ugandan women: A qualitative study. *International journal of gynaecology and obstetrics: the official organ of the International Federation of Gynaecology and Obstetrics* 2017;139 Suppl 1:4-16. doi: 10.1002/ijgo.12378

28. Shakibazadeh E, Namadian M, Bohren MA, et al. Respectful care during childbirth in health facilities globally: a qualitative evidence synthesis. *BJOG* 2017 doi: 10.1111/1471-0528.15015

29. Downe S, Finlayson K, Tuncalp, et al. What matters to women: a systematic scoping review to identify the processes and outcomes of antenatal care provision that are important to healthy pregnant women. *BJOG* 2016;123(4):529-39. doi: 10.1111/1471-0528.13819

30. Benova L, Tunçalp Ö, Moran AC, et al. Not just a number: examining coverage and content of antenatal care in low-income and middle-income countries. *BMJ Global Health* 2018;3(2) doi: 10.1136/bmjgh-2018-000779

31. Baker U, Okuga M, Waiswa P, et al. Bottlenecks in the implementation of essential screening tests in antenatal care: Syphilis, HIV, and anemia testing in rural Tanzania and Uganda. *International journal of gynaecology and obstetrics: the official organ of the International Federation of Gynaecology and Obstetrics* 2015;130 Suppl 1:S43-50. doi: 10.1016/j.ijgo.2015.04.017 [published Online First: 2015/06/10]

32. Baqui AH, Rosecrans AM, Williams EK, et al. NGO facilitation of a government community-based maternal and neonatal health programme in rural India: improvements in equity. *Health Policy and Planning* 2008;23(4):234-43. doi: 10.1093/heapol/czn012

33. Balakrishnan R, Gopichandran V, Chaturvedi S, et al. Continuum of Care Services for Maternal and Child Health using mobile technology - a health system strengthening strategy in low and middle income countries. *BMC Medical Informatics and Decision Making* 2016;16(84):1-8. doi: http://dx.doi.org/10.1186/s12911-016-0326-z

For peer review only

34. WHO. Reproductive health indicators: guidelines for their generation, interpretation and analysis for global monitoring. Geneva: WHO, 2006:63 p.

35. Say L, Barreix M, Chou D, et al. Maternal morbidity measurement tool pilot: study protocol. *Reproductive health* 2016;13(1):69. doi: 10.1186/s12978-016-0164-6 [published Online First: 2016/06/10]

36. Chou D, Tuncalp O, Firoz T, et al. Constructing maternal morbidity - towards a standard tool to measure and monitor maternal health beyond mortality. *BMC pregnancy and childbirth* 2016;16:45. doi: 10.1186/s12884-015-0789-4

37. Andreucci CB, Cecatti JG, Macchetti CE, et al. SISPRENATAL as a tool for evaluating quality of prenatal care. *Revista de saude publica* 2011;45(5):854-64. [published Online First: 2011/08/24]

For peer review only

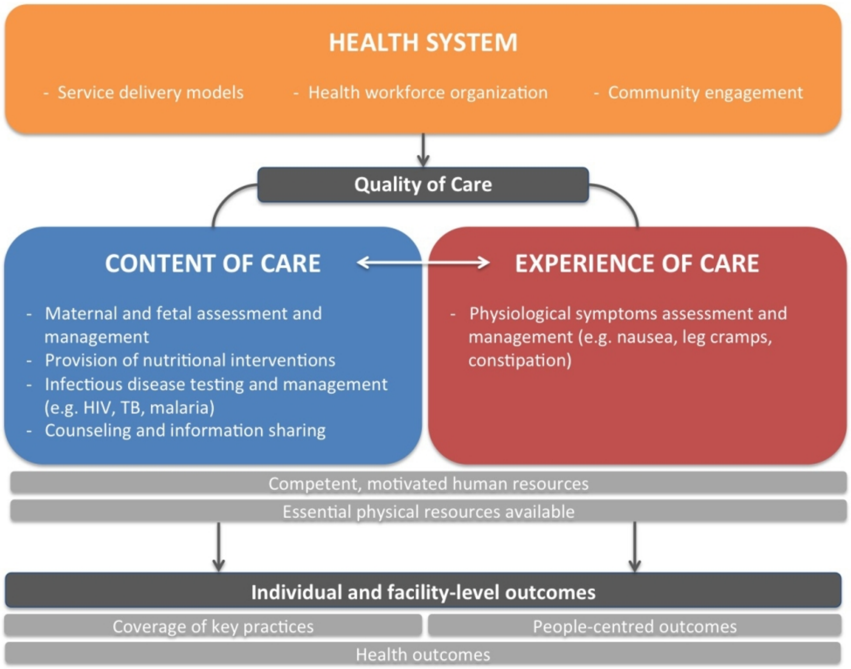


Figure 1: WHO framework for the quality of antenatal care. DHS = Demographic and Health Surveys. MICS = Multiple Indicator Cluster Surveys. WHO = World Health Organization. WoS = Web of Science.

528x396mm (72 x 72 DPI)

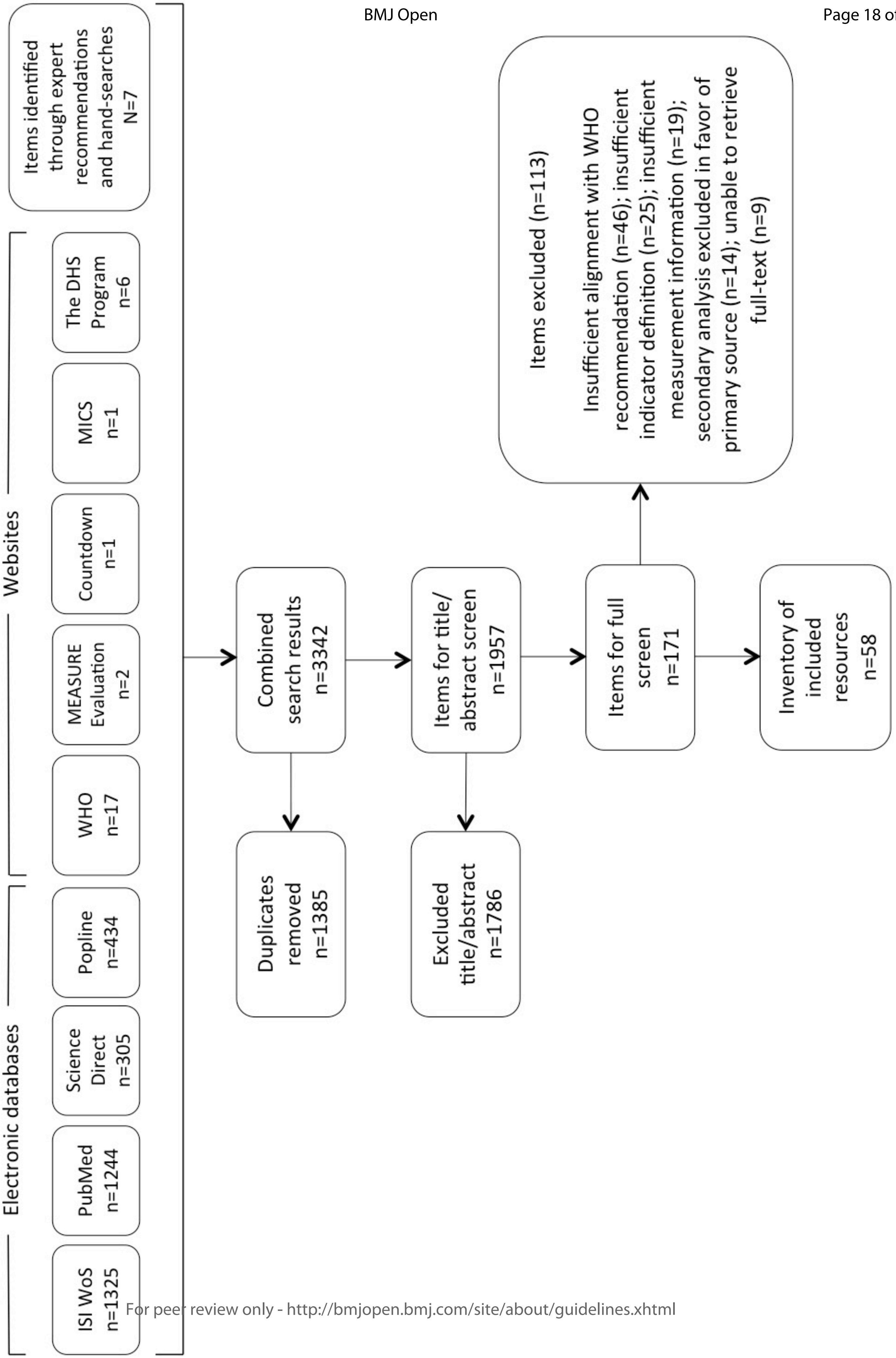
Electronic databases
ISI WoS n=1325
PubMed n=1244
Science Direct n=305
Popline n=434
Websites
WHO n=17
MEASURE Evaluation n=2
Countdown n=1
MICS n=1
The DHS Program n=6
Items identified through expert recommendations and hand-searches N=7
Combined search results n=3342
Duplicates removed n=1385
Items for title/ abstract screen n=1957
Excluded title/abstract n=1786
Items for full screen n=171
Items excluded (n=113)
Insufficient alignment with WHO recommendation (n=46); insufficient indicator definition (n=25); insufficient measurement information (n=19); secondary analysis excluded in favor of primary source (n=14); unable to retrieve full-text (n=9)
Inventory of included resources n=58

**Table A1: Current measures for ANC recommendations**

| Domain | WHO ANC Recommendation | Measure | Definition(s) | Numerator(s) | Denominator(s) |
|---|---|---|---|---|---|
| Content of Care | A.2.1 - Daily oral iron and folic acid supplementation with 30 mg to 60 mg of elemental iron and 400 g (0.4 mg) of folic acid is recommended for pregnant women to prevent maternal anaemia, puerperal sepsis, low birth weight, and preterm birth | Iron and folic acid supplements for pregnant women (%) | Percentage of pregnant women who received iron-folic acid supplementation for 90+ days[1 2]<br><br>Percent of women with a birth in the last two years who received *or bought* iron/folic acid supplements *for at least six months* during their last pregnancy in amounts that were in accordance with recommended protocols[3]<br><br>Proportion of pregnant women given iron and folic acid supplements[4]<br><br>Percentage of respondents who received any iron/folate during pregnancy[5] | Number of pregnant women who received the recommended number of iron/folic acid tablets during last pregnancy[1]<br><br>Number of pregnant women given 90+ IFA tablets[2]<br><br>Number of pregnant women who received *or purchased* the recommended number of iron/folic acid tablets during last pregnancy[3]<br><br>Number of pregnant women given iron and folic acid supplements[4]<br><br>Number of respondents who received any iron/folate during pregnancy[5] | Total number of pregnant women with a birth in last two years[1 3]<br><br>Number of pregnant women in health registers or monthly summary forms[2]<br><br>All eligible pregnancies (a woman having one documented ANC visit (unless the ANC visit is only for termination of pregnancy), OR any data documenting a pregnancy outcome or infant at any point of care)[4]<br><br>Total number of women surveyed who had delivered a live or stillbirth child during the year before the interview date[5] |
| Content of Care | A.2.1 - Daily oral iron and folic acid supplementation with 30 mg to 60 mg of elemental iron and 400 g (0.4 mg) of folic acid is recommended for pregnant women to prevent maternal anaemia, puerperal sepsis, low birth weight, and preterm birth | Preventive treatment with folic acid | Preventive treatment with folic acid: ratio of number of patients that completed folic acid prophylaxis to total number of pregnant women during the year[6] | Number of patients that completed folic acid prophylaxis | Total number of pregnant women during the year |
| Content of Care | A.2.1 - Daily oral iron and folic acid supplementation with 30 mg to 60 mg of elemental iron and 400 g (0.4 mg) of folic acid is recommended for pregnant women to prevent maternal anaemia, puerperal sepsis, low birth weight, and preterm birth | IFA supplementation | Mother consumed at least 100 iron-folic acid tablets during pregnancy[7 8] | Number of mothers who consumed at least 100 iron-folic acid tablets during pregnancy | Total number of study mothers (survey respondents who had a live birth in the reference period for each survey) |
| Content of Care | A.2.1 - Daily oral iron and folic acid supplementation with 30 mg to 60 mg of elemental iron and 400 g (0.4 mg) of folic acid is recommended for pregnant women to prevent maternal anaemia, puerperal sepsis, low birth weight, and preterm birth | ANC visits where the client received a 3-month supply of iron supplements and a 3-month supply of folate supplements | Number of ANC visits at facility per month where the client received a 3-month supply of iron supplements; Number of ANC visits at facility per month where the client received a 3-month supply of folate supplements[9] | Number of ANC visits per month where the client received a 3-month supply of iron supplements; Number of ANC visits at facility per month where the client received a 3-month supply of folate supplements | Total number of ANC visits at facility per month (= new visits + revisits) |

For peer review only

| Domain | WHO ANC Recommendation | Measure | Definition(s) | Numerator(s) | Denominator(s) |
|---|---|---|---|---|---|
| Content of Care | A.2.1 - Daily oral iron and folic acid supplementation with 30 mg to 60 mg of elemental iron and 400 g (0.4 mg) of folic acid is recommended for pregnant women to prevent maternal anaemia, puerperal sepsis, low birth weight, and preterm birth | Percentage of health facilities routinely providing iron and folic acid | Percentage of health facilities routinely providing iron and folic acid during ANC[10] | Number of health facilities where iron and folic acid supplementation is routinely provided during ANC | Total number of facilities surveyed |
| Content of Care | A.2.1 - Daily oral iron and folic acid supplementation with 30 mg to 60 mg of elemental iron and 400 g (0.4 mg) of folic acid is recommended for pregnant women to prevent maternal anaemia, puerperal sepsis, low birth weight, and preterm birth | Folic acid and iron supplementation in pregnancy | 1. Women reporting any folic acid supplementation during their pregnancy; and 2. Women reporting any iron supplementation during their pregnancy[11] | 1. Number of survey respondents reporting any folic acid supplementation during their pregnancy; 2. Number of survey respondents reporting any iron supplementation during their pregnancy<br><br>-OR- 1. Number of antenatal records reporting any folic acid supplementation during pregnancy; 2. Number of antenatal records reporting any iron supplementation during pregnancy | Total number of survey respondents<br><br>-OR- total number of antenatal records reviewed |
| Content of Care | A.2.2 - Intermittent oral iron and folic acid supplementation with 120 mg of elemental iron and 2800 g (2.8 mg) of folic acid once weekly is recommended for pregnant women to improve maternal and neonatal outcomes if daily iron is not acceptable due to side-effects, and in populations with an anaemia prevalence among pregnant women of less than 20% | Iron and folic acid supplements for pregnant women (%) | Percentage of pregnant women who received iron-folic acid supplementation for 90+ days[1]<br><br>Percent of women with a birth in the last two years who received *or bought* iron/folic acid supplements *for at least six months* during their last pregnancy in amounts that were in accordance with recommended protocols[3] | Number of pregnant women who received the recommended number of iron/folic acid tablets during last pregnancy[1]<br><br>Number of pregnant women who received *or purchased* the recommended number of iron/folic acid tablets during last pregnancy[3] | Total number of pregnant women with a birth in last two years |
| Health Systems | B.1.3 - Clinical enquiry about the possibility of intimate partner violence (IPV) should be strongly considered at antenatal care visits when assessing conditions that may be caused or complicated by IPV in order to improve clinical diagnosis and subsequent care, where there is the capacity to provide a supportive response (including referral where appropriate) and where the WHO minimum requirements are met | Percent of health units with at least one service provider trained to care for and refer SGBV survivors | Percent of health facilities in the geographic region of study (e.g., country, region, community) with at least one provider who has been trained within the past three years in the identification, care and support of sexual and gender-based violence (SGBV) survivors[3,12] | Number of health facilities reporting that they have both documented and adopted a protocol for the clinical management of SGBV survivors | Total number of health facilities surveyed |

For peer review only

| Domain | WHO ANC Recommendation | Measure | Definition(s) | Numerator(s) | Denominator(s) |
|---|---|---|---|---|---|
| Health Systems | B.1.3 - Clinical enquiry about the possibility of intimate partner violence (IPV) should be strongly considered at antenatal care visits when assessing conditions that may be caused or complicated by IPV in order to improve clinical diagnosis and subsequent care, where there is the capacity to provide a supportive response (including referral where appropriate) and where the WHO minimum requirements are met | Proportion of women who were asked about physical and sexual violence during a visit to a health unit | Proportion of women who presented to the clinic for any reason who were asked about physical or sexual violence, during a specific period of time (e.g., during the past 12 months)[12 13] | Number of women who were asked, during the course of their service provision at the unit, about any violence that had ever occurred, either physical or sexual, in the geographic area of study (nation, province, state, community).<br><br>If measured with a medical record review, all women's charts that note they were asked if they experienced any physical and sexual violence by a provider would be entered into the numerator.<br><br>If measured in a survey of women based on exit interviews from the health unit, all women leaving the clinic would be asked if a provider asked them if they had ever experienced any physical or sexual violence. All women answering yes would be entered into the numerator. | If the indicator is measured through a record review, this is the number of women's records that were reviewed at the health unit.<br><br>If the indicator is being measured through an exit interview, this is the total number of women interviewed. |
| Content of Care | B.1.5 - Health-care providers should ask all pregnant women about their tobacco use (past and present) and exposure to second-hand smoke as early as possible in the pregnancy and at every antenatal care visit | Proportion of pregnant women whose smoking status was checked at the first antenatal visit | Proportion of pregnant women whose smoking status was checked at the first antenatal visit[4] | Number of pregnant women whose smoking status was checked at the first ANC visit | Total number of eligible pregnancies (a woman having one documented ANC visit (unless the ANC visit is only for termination of pregnancy), OR any data documenting a pregnancy outcome or infant at any point of care) |
| Content of Care | B.1.5 - Health-care providers should ask all pregnant women about their tobacco use (past and present) and exposure to second-hand smoke as early as possible in the pregnancy and at every antenatal care visit | Cigarette smoking screened for in pregnancy | Percentage of women screened for smoking in pregnancy[14] | Number of medical records that indicate screening for smoking in pregnancy occurred | Total number of medical records reviewed |

For peer review only

| Domain | WHO ANC Recommendation | Measure | Definition(s) | Numerator(s) | Denominator(s) |
|---|---|---|---|---|---|
| Content of Care | B.1.7 - In high-prevalence settings, provider-initiated testing and counselling (PITC) for HIV should be considered a routine component of the package of care for pregnant women in all antenatal care settings. In low-prevalence settings, PITC can be considered for pregnant women in antenatal care settings as a key component of the effort to eliminate mother-to-child transmission of HIV, and to integrate HIV testing with syphilis, viral or other key tests, as relevant to the setting, and to strengthen the underlying maternal and child health systems | Pregnant women counselled and tested for HIV and know their results (%) | Percentage of pregnant women attending antenatal care (ANC), labour & delivery (L&D), and postpartum care services (PPC), plus women with known HIV infection attending ANC for a new pregnancy, who received testing and counselling for HIV in the last 12 months and who know their HIV test results[3] | The numerator can be summed from the categories below:<br>- Number of pregnant women who received an HIV test and result during ANC<br>- Number of pregnant women attending labour and delivery (L&D) with unknown HIV status who were tested in the L&D and received results<br>- Women with unknown HIV status attending postpartum services within 72 hours of delivery who were tested and received results<br>- Pregnant women with known HIV infection attending ANC for a new pregnancy. | The denominator is generated through a population estimate of the number of pregnant women giving birth in the last 12 months, which can be obtained from the Central Statistics Office estimates of births or the UN Population Division estimates. In countries with low-level and concentrated epidemics where policies to identify the HIV status of all pregnant women do not exist, the denominator should be adapted to the target population of pregnant women whose HIV status should be assessed. |
| Content of Care | B.1.7 - In high-prevalence settings, provider-initiated testing and counselling (PITC) for HIV should be considered a routine component of the package of care for pregnant women in all antenatal care settings. In low-prevalence settings, PITC can be considered for pregnant women in antenatal care settings as a key component of the effort to eliminate mother-to-child transmission of HIV, and to integrate HIV testing with syphilis, viral or other key tests, as relevant to the setting, and to strengthen the underlying maternal and child health systems | Number of pregnant women tested for HIV | Number of pregnant women attending ANC who are tested for HIV[15]<br><br>Number of ANC registrants receiving HIV testing and counselling[16]<br><br>Proportion of antenatal patients tested[17]<br><br>Proportion of pregnant women undergoing HIV testing and counselling during ANC[18]<br><br>Number (%) of newly registered antenatal cases tested for HIV[19]<br><br>Proportion of pregnant women who received testing and counselling at the first antenatal visit and received their results[4]<br><br>Proportion of pregnant women tested for HIV (of ANC1 and of total expected pregnant women)[20]<br><br>Proportion of pregnant women tested for HIV[2] | Total number of pregnant women attending ANC who are tested[15]<br><br>Number of ANC registrants receiving HIV testing and counselling[16]<br><br>Number of pregnant women with a new HTC test result documented in ANC clinic[18]<br><br>Number of newly registered antenatal cases tested for HIV[19]<br><br>Number of pregnant women who received testing and counselling at the first antenatal visit and received their results[4]<br><br>Number of pregnant women tested for HIV[2 20] | Total # of pregnant women attending ANC[15]<br><br>Total number of ANC registrants[16]<br><br>Total number of pregnant women presenting to ANC clinic without a prior documented positive HIV test result[18]<br><br>All new antenatal cases registered for the first time at any of the Integrated Counselling and Testing Centres that provided PPTCT services during any time of the antenatal period and also those cases who were registered for the first time at the time of admission during labour[19]<br><br>Total number of eligible pregnancies (a woman having one documented ANC visit (unless the ANC visit is only for termination of pregnancy), OR any data documenting a pregnancy outcome or infant at any point of care)[4]<br><br>Number of expected pregnant women[20]<br><br>Number of pregnant women in health registers or monthly summary forms[2] |

For peer review only

| Domain | WHO ANC Recommendation | Measure | Definition(s) | Numerator(s) | Denominator(s) |
|---|---|---|---|---|---|
| Content of Care | B.1.7 - In high-prevalence settings, provider-initiated testing and counselling (PITC) for HIV should be considered a routine component of the package of care for pregnant women in all antenatal care settings. In low-prevalence settings, PITC can be considered for pregnant women in antenatal care settings as a key component of the effort to eliminate mother-to-child transmission of HIV, and to integrate HIV testing with syphilis, viral or other key tests, as relevant to the setting, and to strengthen the underlying maternal and child health systems | Mothers of children 0-23 months who received HIV testing and counselling services during pregnancy (%) | % of mother of children 0-23 months who received HIV testing and counselling services during pregnancy[21] | # of mothers counselled and tested for HIV | Total # of mothers interviewed |
| Content of Care | B.1.7 - In high-prevalence settings, provider-initiated testing and counselling (PITC) for HIV should be considered a routine component of the package of care for pregnant women in all antenatal care settings. In low-prevalence settings, PITC can be considered for pregnant women in antenatal care settings as a key component of the effort to eliminate mother-to-child transmission of HIV, and to integrate HIV testing with syphilis, viral or other key tests, as relevant to the setting, and to strengthen the underlying maternal and child health systems | Percentage of health facilities routinely providing voluntary counselling and testing for HIV/AIDS | Percentage of health facilities routinely providing ANC clients with voluntary counselling and testing for HIV/AIDS[10] | Number of health facilities routinely providing ANC clients with voluntary counselling and testing for HIV/AIDS | Total number of facilities surveyed |
| Content of Care | B.1.7 - In high-prevalence settings, provider-initiated testing and counselling (PITC) for HIV should be considered a routine component of the package of care for pregnant women in all antenatal care settings. In low-prevalence settings, PITC can be considered for pregnant women in antenatal care settings as a key component of the effort to eliminate mother-to-child transmission of HIV, and to integrate HIV testing with syphilis, viral or other key tests, as relevant to the setting, and to strengthen the underlying maternal and child health systems | HIV testing in pregnant women | Percentage of pregnant women with known HIV status[22] | Number of pregnant women attending antenatal clinics (ANC) and/or had a facility-based delivery and were tested for HIV during pregnancy, or already knew they were HIV positive | Population-based denominator: Number of pregnant women who delivered within the past 12 months<br><br>Programme-based denominator: Number of pregnant women who attended an ANC or had a facility-based delivery in the past 12 months |

For peer review only

| Domain | WHO ANC Recommendation | Measure | Definition(s) | Numerator(s) | Denominator(s) |
|---|---|---|---|---|---|
| Content of Care | B.1.7 - In high-prevalence settings, provider-initiated testing and counselling (PITC) for HIV should be considered a routine component of the package of care for pregnant women in all antenatal care settings. In low-prevalence settings, PITC can be considered for pregnant women in antenatal care settings as a key component of the effort to eliminate mother-to-child transmission of HIV, and to integrate HIV testing with syphilis, viral or other key tests, as relevant to the setting, and to strengthen the underlying maternal and child health systems | Antenatal care: tested for syphilis (%) | Percent of women attending antenatal care services who were tested for syphilis[3]<br><br>Proportion of pregnant women tested for syphilis[2 20]<br><br>Proportion attending ANC at least once in a health facility where syphilis tests are available who report having received a test result for syphilis[23] | Number of women in antenatal care (ANC) who were screened for syphilis during pregnancy[3 24]<br><br>Number of women attending ANC services within the past 12 months who were tested for syphilis[25]<br><br>Number of pregnant women who were screened for syphilis at the first antenatal visit[4]<br><br>Number of pregnant women tested for syphilis[2 20]<br><br>Number of women with a live birth in the 12 months prior to the household survey who attended ANC at least once in a health facility where syphilis tests are available who report having received a test result for syphilis[23] | Number of pregnant women attending ANC clinics[3 24]<br><br>Number of women attending ANC services within the past 12 months[25]<br><br>Total number of eligible pregnancies (a woman having one documented ANC visit (unless the ANC visit is only for termination of pregnancy), OR any data documenting a pregnancy outcome or infant at any point of care)[4]<br><br>Number of expected pregnant women[20]<br><br>Number of pregnant women in health registers or monthly summary forms[2]<br><br>Number of women with a live birth in the 12 months prior to the household survey[23] |
| Content of Care | B.1.7 - In high-prevalence settings, provider-initiated testing and counselling (PITC) for HIV should be considered a routine component of the package of care for pregnant women in all antenatal care settings. In low-prevalence settings, PITC can be considered for pregnant women in antenatal care settings as a key component of the effort to eliminate mother-to-child transmission of HIV, and to integrate HIV testing with syphilis, viral or other key tests, as relevant to the setting, and to strengthen the underlying maternal and child health systems | Measures to ensure all pregnant women are screened and treated for syphilis | Existence of formal policy documents and national treatment protocols addressing maternal syphilis screening and treatment[26] | Number of countries with national-level policies addressing maternal syphilis screening and treatment | Total number of countries whose policies were reviewed |

For peer review only

| Domain | WHO ANC Recommendation | Measure | Definition(s) | Numerator(s) | Denominator(s) |
|---|---|---|---|---|---|
| Content of Care | B.2.1 - Daily fetal movement counting, such as with "count-to-ten" kick charts, is only recommended in the context of rigorous research | Antenatal care: fetal movement enquiry (%) | Number of pregnant women ≥28 weeks attending ANC who reported that antenatal care providers routinely enquired about fetal movement at each prenatal visit[27]<br><br>Proportion of survey respondents (gestational age >28 weeks) who reported that their lead maternity carer (LMC) regularly enquired about their baby's movements[28] | Number of pregnant women ≥28 weeks attending ANC who reported that antenatal care providers routinely enquired about fetal movement at each prenatal visit[27]<br><br>Number of pregnant women attending ANC (gestational age ≥28 weeks) who reported that their lead maternity carer regularly enquired about their baby's movements[28] | Total number of pregnant women ≥28 weeks attending ANC in the reporting period (who were surveyed) |
| Content of Care | C.1 - A seven-day antibiotic regimen is recommended for all pregnant women with asymptomatic bacteriuria (ASB) to prevent persistent bacteriuria, preterm birth and low birth weight | Number of episodes where immediate treatment was given per episode of positive urinalysis | Number of episodes where immediate treatment was given per episode of positive urinalysis[29] | Number of episodes of positive (abnormal) urinalysis where women received immediate treatment with antibiotics | Number of episodes of positive (abnormal) urinalysis in all charts reviewed |
| Content of Care | C.4 - In endemic areas, preventive anthelminthic treatment is recommended for pregnant women after the first trimester as part of worm infection reduction programmes | Percent of pregnant women who receive anthelminthic treatment during pregnancy | In areas of moderate to high endemicity of helminths (parasitic worms), the percent of pregnant women who receive presumptive anthelminthic treatment during their pregnancy. According to the 1998 IVACG/WHO/UNICEF "Guidelines for the Use of Iron Supplements to Prevent and Treat Iron Deficiency Anemia," treatment should be done once in the second and third trimester. In areas of low endemicity: the percent of pregnant women who received prescribed treatment during their pregnancy.[3]<br><br>Percentage of respondents who received deworming with albendazole during pregnancy[5]<br><br>Proportion of pregnant women provided deworming[2] | # of pregnant women who receive presumptive antihelminthic treatment[3]<br><br>Number of respondents who received deworming with albendazole during pregnancy[5]<br><br>Number of pregnant women provided deworming[2] | Total # of pregnant women[3]<br><br>Total number of women surveyed who had delivered a live or stillbirth child during the year before the interview date[5]<br><br>Number of pregnant women in health registers or monthly summary forms[2] |
| Content of Care | C.5 - Tetanus toxoid vaccination is recommended for all pregnant women, depending on previous tetanus vaccination exposure, to prevent neonatal mortality from tetanus | Percentage of newborns protected at birth from tetanus (takes into account mother's doses prior to pregnancy) | Percentage of newborns protected against tetanus[30] | Number of mothers with a live birth in the year prior to the survey who received two doses of tetanus toxoid vaccine within the appropriate interval prior to the infant's birth | Total number of women ages 15–49 with a live birth in the year prior to the survey |
| Content of Care | C.5 - Tetanus toxoid vaccination is recommended for all pregnant women, depending on previous tetanus vaccination exposure, to prevent neonatal mortality from tetanus | Percentage of health facilities offering tetanus toxoid vaccination services | Percentage of health facilities where tetanus toxoid vaccination services are available each day that ANC services are provided[10] | Number of health facilities where tetanus toxoid vaccination services are available each day that ANC services are provided | Total number of facilities surveyed |

For peer review only

| Domain | WHO ANC Recommendation | Measure | Definition(s) | Numerator(s) | Denominator(s) |
|---|---|---|---|---|---|
| Content of Care | C.5 - Tetanus toxoid vaccination is recommended for all pregnant women, depending on previous tetanus vaccination exposure, to prevent neonatal mortality from tetanus | Percent of women who received at least two doses of tetanus-toxoid vaccine in their last pregnancy | Proportion of pregnant women receiving at least two doses of tetanus-toxoid vaccine (TT2+)[2 3 7 8]<br><br>Percentage of respondents who received tetanus toxoid 2 doses during pregnancy or lifetime 5 doses[5] | Total TT2 + TT3 + TT4 + TT5 x 100 (Where TT2, TT3, TT4, TT5 refer to the 2nd, 3rd, 4th, or 5th dose of tetanus-toxoid vaccine administered)<br><br>Number of pregnant women given TT2 immunization[2]<br><br>Number of respondents who received tetanus toxoid 2 doses during pregnancy or lifetime 5 doses[5]<br><br>Number of respondents who received two or more TT injections[8] | Total # of live births<br><br>Number of pregnant women in health registers or monthly summary forms[2]<br><br>Total number of women surveyed who had delivered a live or stillbirth child during the year before the interview date[5]<br><br>Total number of ever married women surveyed in the age group 15-49 years[8] |
| Content of Care | C.5 - Tetanus toxoid vaccination is recommended for all pregnant women, depending on previous tetanus vaccination exposure, to prevent neonatal mortality from tetanus | Number of third trimester ANC visits protected against tetanus (at least TT2) per month | Number of third trimester (≥28 weeks) ANC visits protected against tetanus (at least TT2) per month[9] | Number of third trimester ANC visits protected against tetanus (at least TT2) per month | Total number of third trimester (>28 weeks) ANC visits at facility per month |
| Content of Care | C.5 - Tetanus toxoid vaccination is recommended for all pregnant women, depending on previous tetanus vaccination exposure, to prevent neonatal mortality from tetanus | Rate of tetanus vaccination | Rate of tetanus vaccination: ratio of number of pregnant women with complete vaccinations (verified at delivery) to total number of pregnant women during the year[6] | Number of pregnant women with complete vaccinations (verified at delivery) | Total number of pregnant women during the year |
| Content of Care | C.5 - Tetanus toxoid vaccination is recommended for all pregnant women, depending on previous tetanus vaccination exposure, to prevent neonatal mortality from tetanus | Mothers who received at least two tetanus toxoid vaccinations before the birth of their youngest child (%) | Percentage of mothers with children age 0-23 months who received at least two tetanus toxoid vaccinations before the birth of their youngest child[21]<br><br>Mothers of children 0-11 months who received ≥2 antenatal tetanus toxoid vaccinations[31] | # of Mothers who receiving at least 2 TT injections (Q19 + Q21=>2)[21]<br><br>Mothers of children 0-11 months who received ≥2 antenatal tetanus toxoid vaccinations[31] | Total # mothers interviewed[21]<br><br>Total number of mothers of children 0-11 months surveyed[31] |

For peer review only

| Domain | WHO ANC Recommendation | Measure | Definition(s) | Numerator(s) | Denominator(s) |
|---|---|---|---|---|---|
| Content of Care | C.6 - In malaria-endemic areas in Africa, intermittent preventive treatment with sulfadoxine-pyrimethamine (IPTp-SP) is recommended for all pregnant women. Dosing should start in the second trimester, and doses should be given at least one month apart, with the objective of ensuring that at least three doses are received | Intermittent preventive therapy for malaria during pregnancy (IPTp) (%) | Percentage of women who received three or more doses of intermittent preventive treatment during antenatal care visits during their last pregnancy | Number of women receiving three or more doses of recommended treatment[24]<br><br>Number of women who received three or more doses of a recommended prophylactic antimalarial drug treatment, at least one of which was received during an ANC visit, to prevent malaria during their last pregnancy that led to a live birth within the last two years[32] | Total number of pregnant women surveyed who delivered a live birth in the last 2 years |
| Content of Care | C.6 - In malaria-endemic areas in Africa, intermittent preventive treatment with sulfadoxine-pyrimethamine (IPTp-SP) is recommended for all pregnant women. Dosing should start in the second trimester, and doses should be given at least one month apart, with the objective of ensuring that at least three doses are received | Percentage of pregnant women attending ANC who receive a first (and/or second/third) dose of IPTp under direct observation | Proportion of women attending ANC clinics who receive IPT1/IPT2/IPT3 as directly observed treatment by a health worker to maximize compliance[33] | Number of pregnant women who receive IPT1 (IPT2/IPT3) under observation | Number of first ANC visits (stays the same regardless of IPT1/IPT2/IPT3) |
| Content of Care | C.6 - In malaria-endemic areas in Africa, intermittent preventive treatment with sulfadoxine-pyrimethamine (IPTp-SP) is recommended for all pregnant women. Dosing should start in the second trimester, and doses should be given at least one month apart, with the objective of ensuring that at least three doses are received | Number of third trimester ANC visits receiving IPT2 (or more) per month | Number of third trimester ($\geq$28 weeks) ANC visits receiving IPT2 (or more) per month[9] | Number of third trimester ANC visits receiving IPT2 (or more) per month | Total number of third trimester (>28 weeks) ANC visits at facility per month |
| Content of Care | C.6 - In malaria-endemic areas in Africa, intermittent preventive treatment with sulfadoxine-pyrimethamine (IPTp-SP) is recommended for all pregnant women. Dosing should start in the second trimester, and doses should be given at least one month apart, with the objective of ensuring that at least three doses are received | Percentage of health facilities routinely providing preventative anti-malarial medication | Percentage of health facilities routinely providing preventative anti-malarial medication to ANC clients[10] | Number of health facilities routinely providing preventative anti-malarial medication to ANC clients | Total number of facilities surveyed |

For peer review only

| Domain | WHO ANC Recommendation | Measure | Definition(s) | Numerator(s) | Denominator(s) |
|---|---|---|---|---|---|
| Content of Care | C.7 - Oral pre-exposure prophylaxis (PrEP) containing tenofovir disoproxil fumarate (TDF) should be offered as an additional prevention choice for pregnant women at substantial risk of HIV infection as part of combination prevention approaches | PrEP coverage | % using PrEP in priority PrEP populations[25] | Number of members of the selected PrEP priority groups using PrEP within the last 12 months | Number of people in the selected PrEP priority groups |
| Health Systems | E.1 - It is recommended that each pregnant woman carries her own case notes during pregnancy to improve continuity, quality of care and her pregnancy experience | Percent of mothers of children 0-23 months with a maternal health card | Percent of mothers of children 0-23 months with a maternal health card (interviewer-confirmed)[21] | # of mothers with maternal health cards | Total # of mothers interviewed |
| Health Systems | E.6 - Policy-makers should consider educational, regulatory, financial, and personal and professional support interventions to recruit and retain qualified health workers in rural and remote areas | Health worker density and distribution | Number of health workers per 1000 population[24] | Number of health workers by cadre | Total population |
| Health Systems | E.7 - Antenatal care models with a minimum of eight contacts are recommended to reduce perinatal mortality and improve women's experience of care | Antenatal care (eight or more visits) (%) | Percentage of women aged 15−49 years with a live birth in a given time period who received antenatal care, eight times or more[1] | Number of women aged 15−49 years with a live birth in a given time period who received antenatal care eight or more times | Total number of women aged 15−49 years with a live birth in the same period<br><br>All female respondents in the household who have reached he age of menstruation (12 years and older) and were/are pregnant in the last 12 months[34] |
| Content of Care | Counselling about family planning | Percentage of health facilities routinely counselling ANC clients about family planning | Percentage of health facilities routinely counselling ANC clients about family planning[10] | Number of health facilities routinely counselling ANC clients about family planning | Total number of facilities surveyed |
| Content of Care | Counselling about family planning | Counselling on family planning | Percentage of pregnant women counselled on family planning[2] | Number of pregnant women counselled on family planning | Number of pregnant women in health registers or monthly summary forms |
| Content of Care | Blood pressure monitored | Blood pressure: At least six measures in routine antenatal care programme | National guidelines recommending at least six measures of blood pressure in routine antenatal care[35] | Number of national guidelines recommending at least six measures of blood pressure in routine antenatal care | Number of national guidelines reviewed |

For peer review only

| Domain | WHO ANC Recommendation | Measure | Definition(s) | Numerator(s) | Denominator(s) |
|---|---|---|---|---|---|
| Content of Care | Blood pressure monitored | Blood pressure: At least # measure(s) during pregnancy | Woman-reported content of antenatal care received at least once (or "x" times) during pregnancy: blood pressure measured | Number of women with at least one antenatal contact with any provider during pregnancy who reported that their blood pressure was measured at least once during pregnancy[36] | (1) all women with a live birth in the last 12 months prior to the survey; or (2) women who had at least one antenatal contact with any provider during that pregnancy[36] |
| | | | | Blood pressure checked at least five times during antenatal care[37] | All mothers in the sample who underwent prenatal care and delivery in the public health service whose children were born alive[37] |
| Content of Care | Blood pressure monitored | ANC visits with blood pressure reading documented | Number of ANC visits at facility per month with blood pressure reading documented[9] | Number of ANC visits per month with a documented blood pressure reading | Total number of ANC visits at facility per month (= new visits + revisits) |
| Content of Care | Blood pressure monitored | Receipt of basic ANC services | Receipt of all three basic services during antenatal care: blood pressure measurement, blood sample taken, urine sample taken[38] | Number of women surveyed who received all three basic services during antenatal care: blood pressure measurement, blood sample taken, urine sample taken | Total number of women surveyed |
| Content of Care | Blood pressure monitored | Blood pressure measured at last exam | Percentage of women who had their blood pressure measured at their most recent ANC visit | Number of survey/interview respondents reporting that their blood pressure was measured at their most recent ANC visit | Total number of pregnant women using ANC during the data collection period who consented and were selected by systematic random sampling at the health facilities[39] |
| | | | | Number of pregnant women with blood pressure recorded[2] | Total number of client exit interviews[40] |
| | | | | | Number of pregnant women in health registers or monthly summary forms[2] |

For peer review only

| Domain | WHO ANC Recommendation | Measure | Definition(s) | Numerator(s) | Denominator(s) |
|---|---|---|---|---|---|
| Health Systems | First antenatal care visit in the first trimester | Percentage of pregnant women who began ANC during the first trimester of gestation | Proportion of pregnant women who initiate ANC in the first trimester[41]<br><br>Proportion of women with estimated gestational age at first antenatal visit < 12 weeks[42]<br><br>Proportion of women who receive antenatal assessments by 13 weeks of pregnancy[43]<br><br>Antenatal care initiated in the first trimester (<14 weeks) and minimum of 4 visits[44]<br><br>Percentage of women among whom the first medical visit occurred in the first trimester of pregnancy (or timeliness)[45]<br><br>Mothers recorded in the antenatal records with ANC in first trimester (<13 weeks)[46]<br><br>Women presenting in first trimester for first antenatal visit[14] | Number of pregnant women initiating ANC in first trimester[41]<br><br>Number of women attending ANC who began ANC during the first trimester[14]<br><br>Number of women whose first antenatal care visit was <12 weeks of gestational age[47]<br><br>Number of women whose first prenatal visit was ≤12 weeks[48]<br><br>Number of women with estimated gestational age at first antenatal visit < 12 weeks[42]<br><br>Number of pregnant women seen for ANC booking by 13 weeks 0 days[43]<br><br>Antenatal care initiated in the first trimester (<14 weeks) and minimum of 4 visits[44]<br><br>Number of women aged 15-49 surveyed who reported an obstetric episode from 2004-2009 and whose first medical visit occurred in the first trimester of pregnancy[45]<br><br>Mothers recorded in the antenatal records with ANC in first trimester (<13 weeks)[46] | Total number of women attending ANC[49]<br><br>All female respondents reporting a birth in the last five years[50]<br><br>All female respondents in the household who have reached he age of menstruation (12 years and older) and were/are pregnant in the last 12 months[34]<br><br>Total number of pregnant women using ANC during the data collection period who consented and were selected by systematic random sampling at the health facilities[39]<br><br>Total number of survey respondents; also total number of respondent electronic records[47]<br><br>Total number of medical charts reviewed[14,42,46,48]<br><br>Number of pregnant women[43]<br><br>Number of women sampled[44]<br><br>Total number of women aged 15-49 surveyed who reported an obstetric episode from 2004-2009[45] |
| Health Systems | First antenatal care visit in the first trimester | Timing of first antenatal visit (%) | Percentage of pregnant women aged 15–49 years who had their first antenatal visit in the first trimester[1] | Number of pregnant women aged 15-49 who had their first antenatal visit in the first trimester | Total number of women aged 15-49 years with at least one antenatal care visit in the same period |

For peer review only

| Domain | WHO ANC Recommendation | Measure | Definition(s) | Numerator(s) | Denominator(s) |
|---|---|---|---|---|---|
| Content of Care | Counselling about birth preparedness | Receiving ANC counselling about birth preparedness | Percentage of pregnant women who received at least one counselling session on birth preparedness | Number of pregnant women attending ANC who, during this visit or a previous visit, had a provider discuss things they should have in preparation for delivery (e.g. planning in case of emergency, things to bring to a facility, things to prepare at home for the delivery)[51]<br><br>Number of women who had at least one antenatal contact with any provider during that pregnancy and report they were counselled about birth preparedness at least once during pregnancy[36]<br><br>Number of pregnant women counselled on emergency preparedness[2] | Total number of pregnant women attending ANC who were surveyed in the reporting period[51]<br><br>(1) all women with a live birth in the last 12 months prior to the survey; or (2) women who had at least one antenatal contact with any provider during that pregnancy[36]<br><br>Number of pregnant women in health registers or monthly summary forms[2] |
| Content of Care | Counselling about birth preparedness | Exposure to birth preparedness messages | (a) formal exposure, i.e. a respondent who received a key chain and/or was counselled using a birth preparedness flip-chart; and (b) exposure to specific birth preparedness messages, i.e. a respondent who reported having heard a specific message[52] | Formal exposure: total number of respondents who received a key chain and/or were counselled using a birth preparedness flip-chart<br><br>Message exposure: Total number of respondents exposed to specific birth preparedness messages: (a) "A pregnant women should make four antenatal care visits with a trained health worker"; (b) "A newborn should be breastfed for the first time immediately after birth"; and, (c) "A mother and newborn should have their health checked by a trained health worker within days after birth" | Total number of mothers of live infants aged less than one year at the time of the survey |

*Note: Six of the 58 resources generated by the scoping review do not appear in the truncated table above. These six resources provided important information about indicator methodology (e.g. method of measurement, measurement frequency, data sources) and current methodological work.[53-58]

For peer review only

**References**


1. MoNITOR. Maternal and Newborn Indicator Mapping. Geneva: World Health Organization, 2017.

2. Dwivedi V, Drake M, Rawlins B, et al. A Review of the Maternal and Newborn Health Content of National Health Management Information Systems in 13 Countries in Sub-Saharan Africa and South Asia. Washington, D.C.: Maternal and Child Health Integrated Program, 2014.

3. MEASURE Evaluation. Summary List of Indicators Chapel Hill, North Carolina, USA: MEASURE Evaluation; 2017 [Available from: https://www.measureevaluation.org/prh/rh_indicators/indicator-summary accessed 27 Aug 2017.

4. Flenady V, Wojcieszek AM, Fjeldheim I, et al. eRegistries: indicators for the WHO Essential Interventions for reproductive, maternal, newborn and child health. *Bmc Pregnancy and Childbirth* 2016;16 doi: 10.1186/s12884-016-1049-y

5. Hodgins S, McPherson R, Suvedi BK, et al. Testing a scalable community-based approach to improve maternal and neonatal health in rural Nepal. *Journal of perinatology : official journal of the California Perinatal Association* 2010;30(6):388-95. doi: 10.1038/jp.2009.181 [published Online First: 2009/11/13]

6. Marin GH, Silberman M, Uriarte A, et al. Healthcare model based on personalised attention: impact on maternal mortality and health system quality. *Quality in primary care* 2011;19(5):311-6. [published Online First: 2011/12/22]

7. Baqui AH, Rosecrans AM, Williams EK, et al. NGO facilitation of a government community-based maternal and neonatal health programme in rural India: improvements in equity. *Health Policy and Planning* 2008;23(4):234-43. doi: 10.1093/heapol/czn012

8. Kavitha N. Are Young Mothers in India Deprived of Maternal Health Care Services? A Comparative Study of Urban and Rural Areas. *Journal of Health Management* 2015;17(2):204-20. doi: http://dx.doi.org/10.1177/0972063415575809

9. Mwaniki MK, Vaid S, Chome IM, et al. Improving service uptake and quality of care of integrated maternal health services: the Kenya Kwale District improvement collaborative. *BMC health services research* 2014;14:416. doi: 10.1186/1472-6963-14-416 [published Online First: 2014/09/23]

10. Keating J. Nigeria Baseline Health Facility Survey, 2005: [Chapel Hill, North Carolina], University of North Carolina at Chapel Hill, Carolina Population Center [CPC], MEASURE Evaluation, 2006 Oct., 2006:[78] p.

11. Ogundipe O, Hoyo C, Ostbye T, et al. Factors associated with prenatal folic acid and iron supplementation among 21,889 pregnant women in Northern Tanzania: a cross-sectional hospital-based study. *BMC public health* 2012;12:481. doi: 10.1186/1471-2458-12-481 [published Online First: 2012/06/28]

12. Bloom S. Violence against Women and Girls: A Compendium of Monitoring and Evaluation Indicators. Chapel Hill, North Carolina, USA: MEASURE Evaluation, 2008.

13. Bunn MY, Higa NA, Parker WJ, et al. Domestic violence screening in pregnancy. *Hawaii medical journal* 2009;68(10):240-2. [published Online First: 2009/12/17]


For peer review only

14. Reeve C, Banfield S, Thomas A, et al. Community outreach midwifery-led model improves antenatal access in a disadvantaged population. *The Australian journal of rural health* 2016;24(3):200-6. doi: 10.1111/ajr.12249 [published Online First: 2015/09/24]

15. Chabikuli ON, Gwarzo U, Olufunso A, et al. Closing the prevention of mother-to-child transmission gap in Nigeria: an evaluation of service improvement intervention in Nigeria. *South African Family Practice* 2013;55(1):96-102.

16. Dako-Gyeke P, Dornoo B, Ayisi Addo S, et al. Towards elimination of mother-to-child transmission of HIV in Ghana: an analysis of national programme data. *International journal for equity in health* 2016;15:5. doi: 10.1186/s12939-016-0300-5 [published Online First: 2016/01/14]

17. Draper B, Abdullah F. A review of the prevention of mother-to-child transmission programme of the Western Cape provincial government, 2003 - 2004. *South African Medical Journal* 2008;98(6):431-4.

18. Herce ME, Mtande T, Chimbwandira F, et al. Supporting Option B+ scale up and strengthening the prevention of mother-to-child transmission cascade in central Malawi: results from a serial cross-sectional study. *BMC infectious diseases* 2015;15:328. doi: 10.1186/s12879-015-1065-y [published Online First: 2015/08/13]

19. Mukherjee S, Ghosh S, Goswami DN, et al. Performance evaluation of PPTCT (Prevention of parent to child transmission of HIV) programme: an experience from West Bengal. *The Indian journal of medical research* 2012;136(6):1011-9. [published Online First: 2013/02/09]

20. Delvaux T, Samreth S, Barr-DiChiara M, et al. Linked response for prevention, care, and treatment of HIV/AIDS, STIs, and reproductive health issues: results after 18 months of implementation in five operational districts in Cambodia. *Journal of acquired immune deficiency syndromes (1999)* 2011;57(3):e47-55. doi: 10.1097/QAI.0b013e318216d5cf [published Online First: 2011/03/17]

21. Bali K, Wheeler R, Davoodnia R, et al. Partnership for Maternal and Neonatal Health - West Pokot District Child Survival and Health Program. New York: Doctors of the World, 2007.

22. Global AIDS Response. Global AIDS Response Progress Reporting 2016. Geneva: UNAIDS, 2016.

23. Baker U, Okuga M, Waiswa P, et al. Bottlenecks in the implementation of essential screening tests in antenatal care: Syphilis, HIV, and anemia testing in rural Tanzania and Uganda. *International journal of gynaecology and obstetrics: the official organ of the International Federation of Gynaecology and Obstetrics* 2015;130 Suppl 1:S43-50. doi: 10.1016/j.ijgo.2015.04.017 [published Online First: 2015/06/10]

24. WHO. Global Reference List of 100 Core Health Indicators. Geneva: World Health Organization, 2015.

25. WHO. Consolidated Strategic Information Guidelines for HIV in the Health Sector. Geneva: World Health Organization, 2015.

26. Hossain M, Broutet N, Hawkes S. The elimination of congenital syphilis: A comparison of the proposed world health organization action plan for the elimination of congenital syphilis with existing national maternal and congenital syphilis policies. *Sexually Transmitted Diseases* 2007;34(7):S22-S30. doi: 10.1097/olq.0000261049.84824.40


For peer review only

27. Olagbuji BN, Igbarumah S, Akintayo AA, et al. Maternal understanding of fetal movement in third trimester: a means for fetal monitoring and reducing stillbirth. *Nigerian journal of clinical practice* 2014;17(4):489-94. doi: 10.4103/1119-3077.134049 [published Online First: 2014/06/10]

28. Peat AM, Stacey T, Cronin R, et al. Maternal knowledge of fetal movements in late pregnancy. *The Australian & New Zealand journal of obstetrics & gynaecology* 2012;52(5):445-9. doi: 10.1111/j.1479-828X.2012.01462.x [published Online First: 2012/07/04]

29. Bookallil M, Chalmers E, Andrew B. Challenges in preventing pyelonephritis in pregnant women in Indigenous communities. *Rural and remote health* 2005;5(3):395. [published Online First: 2005/08/09]

30. Countdown to 2015. Building a Future for Women and Children: The 2012 Report. Washington, D.C.: World Health Organization and UNICEF, 2012.

31. Teferi E, Alemu H, Bulto T, et al. A descriptive study of the changes in coverage of preventive and promotive interventions before and after the introduction of integrated community case management (ICCM) in Ethiopia. *Ethiopian medical journal* 2014;52 Suppl 3:151-5. [published Online First: 2015/04/08]

32. MEASURE Evaluation, MEASURE DHS, President's Malaria Initiative, et al. Household Survey Indicators for Malaria Control. Chapel Hill, North Carolina, USA: MEASURE Evaluation, 2013.

33. WHO. Malaria in pregnancy: Guidelines for measuring key monitoring and evaluation indicators. Geneva: WHO, 2007:[38] p.

34. Economic Growth Center (EGC) at Yale University, Institute of Statistical SERIaUoG, . Ghana Socio-economic Panel Survey: Household Instrument Wave One (2009) Part B. Living Standards Measurement Study Questionnaire. Legon, Ghana: University of Ghana, 2009.

35. Beeckman K, Frith L, Gottfreosdottir H, et al. Measuring antenatal care use in Europe: is the content and timing of care in pregnancy tool applicable? *International Journal of Public Health* 2017;62(5):583-90. doi: 10.1007/s00038-017-0959-4

36. Marchant T, Tilley-Gyado RD, Tessema T, et al. Adding Content to Contacts: Measurement of High Quality Contacts for Maternal and Newborn Health in Ethiopia, North East Nigeria, and Uttar Pradesh, India. *PloS one* 2015;10(5) doi: 10.1371/journal.pone.0126840

37. Melo EC, de Oliveira RR, Mathias TAD. Factors associated with the quality of prenatal care: an approach to premature birth. *Revista Da Escola De Enfermagem Da Usp* 2015;49(4):540-48. doi: 10.1590/s0080-623420150000400002

38. Wilunda C, Tanaka S, Putoto G, et al. Evaluation of a maternal health care project in South West Shoa Zone, Ethiopia: before-and-after comparison. *Reproductive health* 2016;13(1):95. doi: 10.1186/s12978-016-0213-1 [published Online First: 2016/08/21]

39. Ejigu T, Woldie M, Kifle Y. Quality of antenatal care services at public health facilities of Bahir-Dar special zone, Northwest Ethiopia. *BMC health services research* 2013;13:443. doi: 10.1186/1472-6963-13-443 [published Online First: 2013/10/29]

For peer review only

40. McNabb M, Chukwu E, Ojo O, et al. Assessment of the quality of antenatal care services provided by health workers using a mobile phone decision support application in northern Nigeria: a pre/post-intervention study. *PloS one* 2015;10(5):e0123940. doi: 10.1371/journal.pone.0123940 [published Online First: 2015/05/06]

41. Mbuagbaw L, Medley N, Darzi AJ, et al. Health system and community level interventions for improving antenatal care coverage and health outcomes. *The Cochrane database of systematic reviews* 2015(12):Cd010994. doi: 10.1002/14651858.CD010994.pub2 [published Online First: 2015/12/02]

42. Rumbold AR, Bailie RS, Si D, et al. Delivery of maternal health care in Indigenous primary care services: baseline data for an ongoing quality improvement initiative. *BMC pregnancy and childbirth* 2011;11:16. doi: 10.1186/1471-2393-11-16 [published Online First: 2011/03/10]

43. National Institute for Health and Care Excellence. NICE menu indicator guidance: Indicator CCG30. London: NICE, 2016.

44. Yucel C, Taskin L, Low LK. Validity and reliability of the Turkish version of the Optimality Index-US (OI-US) to assess maternity care outcomes. *Midwifery* 2015;31(12):1135-42. doi: 10.1016/j.midw.2015.08.003 [published Online First: 2015/08/25]

45. Servan-Mori E, Contreras-Loya D, Gomez-Dantes O, et al. Use of performance metrics for the measurement of universal coverage for maternal care in Mexico. *Health Policy and Planning* 2017;32(5):625-33. doi: 10.1093/heapol/czw161

46. Kildea S, Gao Y, Rolfe M, et al. Remote links: Redesigning maternity care for Aboriginal women from remote communities in Northern Australia - A comparative cohort study. *Midwifery* 2016;34:47-57. doi: 10.1016/j.midw.2016.01.009 [published Online First: 2016/03/15]

47. Liu L, Li M, Yang L, et al. Measuring coverage in MNCH: a validation study linking population survey derived coverage to maternal, newborn, and child health care records in rural China. *PloS one* 2013;8(5):e60762. doi: 10.1371/journal.pone.0060762 [published Online First: 2013/05/15]

48. Lu J, Shen JJ, Chen G, et al. Regional disparities in prenatal care services in rural China. *Asia-Pacific journal of public health* 2011;23(5):682-9. doi: 10.1177/1010539511418356 [published Online First: 2011/08/20]

49. Doubova SV, Perez-Cuevas R, Ortiz-Panozo E, et al. Evaluation of the quality of antenatal care using electronic health record information in family medicine clinics of Mexico City. *BMC pregnancy and childbirth* 2014;14:168. doi: 10.1186/1471-2393-14-168 [published Online First: 2014/06/03]

50. The DHS Program. Model Woman's Questionnaire. DHS Questionnaires and Manuals - Phase 7. Rockville, Maryland, USA: ICF International, 2017.

51. The DHS Program. Measure DHS service provision assessment survey: ANC client exit interview. SPA Questionnaires. Rockville, Maryland, USA: ICF International, 2012.

52. McPherson RA, Khadka N, Moore JM, et al. Are birth-preparedness programmes effective? Results from a field trial in Siraha district, Nepal. *Journal of health, population, and nutrition* 2006;24(4):479-88. [published Online First: 2007/06/27]

53. MICS. Questionnaire for Individual Women. MICS6 Questionnaires. New York: UNICEF, 2017.

For peer review only

54. The DHS Program. Measure DHS service provision assessment survey: Inventory questionnaire. SPA Questionnaires. Rockville, Maryland, USA: ICF International, 2012.

55. The DHS Program. Measure DHS service provision assessment survey: Observation of ANC consultation. SPA Questionnaires. Rockville, Maryland, USA: ICF International, 2012.

56. The DHS Program. Measure DHS service provision assessment survey: Health worker interview. SPA Questionnaires. Rockville, Maryland, USA: ICF International, 2012.

57. The DHS Program. Measuring service availability and readiness: A health facility assessment methodology for monitoring health system strengthening. Service Readiness Indicators. Geneva: WHO and USAID, 2012.

58. The DHS Program. Malaria Indicator Survey Model Woman's Questionnaire. Malaria Surveys. Rockville, Maryland, USA: The DHS Program, 2016.

For peer review only

**Table A2: ANC recommendations and established good clinical practices without existing measures**

| Domain | WHO ANC Recommendation or Established Good Clinical Practice |
|---|---|
| Content of Care | A.1.1 - Counselling about healthy eating and keeping physically active during pregnancy is recommended for pregnant women to stay healthy and to prevent excessive weight gain during pregnancy |
| Content of Care | A.1.2 - In undernourished populations, nutrition education on increasing daily energy and protein intake is recommended for pregnant women to reduce the risk of low-birth-weight neonates |
| Content of Care | A.1.3 - In undernourished populations, balanced energy and protein dietary supplementation is recommended for pregnant women to reduce the risk of stillbirths and small-for-gestational-age neonates |
| Content of Care | A.3 - In populations with low dietary calcium intake, daily calcium supplementation (1.5–2.0 g oral elemental calcium) is recommended for pregnant women to reduce the risk of pre-eclampsia |
| Content of Care | A.4 - Vitamin A supplementation is only recommended for pregnant women in areas where vitamin A deficiency is a severe public health problem to prevent night blindness |
| Content of Care | A.5 - Zinc supplementation for pregnant women is only recommended in the context of rigorous research |
| Content of Care | A.10 - For pregnant women with high daily caffeine intake (more than 300 mg per day), lowering daily caffeine intake during pregnancy is recommended to reduce the risk of pregnancy loss and low-birth-weight neonates |
| Content of Care | B.1.1 - Full blood count testing is the recommended method for diagnosing anaemia in pregnancy. In settings where full blood count testing is not available, on-site haemoglobin testing with a haemoglobinometer is recommended over the use of the haemoglobin colour scale as the method for diagnosing anaemia in pregnancy |
| Content of Care | B.1.2 - Midstream urine culture is the recommended method for diagnosing asymptomatic bacteriuria (ASB) in pregnancy. In settings where urine culture is not available, on-site midstream urine Gramstaining is recommended over the use of dipstick tests as the method for diagnosing ASB in pregnancy |
| Content of Care | B.1.4 - Hyperglycaemia first detected at any time during pregnancy should be classified as either gestational diabetes mellitus (GDM) or diabetes mellitus in pregnancy, according to WHO criteria |
| Content of Care | B.1.6 - Health-care providers should ask all pregnant women about their use of alcohol and other substances (past and present) as early as possible in the pregnancy and at every antenatal care visit |
| Content of Care | B.1.8 - In settings where the tuberculosis (TB) prevalence in the general population is 100/100 000 population or higher, systematic screening for active TB should be considered for pregnant women as part of antenatal care |
| Content of Care | B.2.4 - One ultrasound scan before 24 weeks of gestation (early ultrasound) is recommended for pregnant women to estimate gestational age, improve detection of fetal anomalies and multiple pregnancies, reduce induction of labour for post-term pregnancy, and improve a woman's pregnancy experience |
| Content of Care | C.2 - Antibiotic prophylaxis is only recommended to prevent recurrent urinary tract infections in pregnant women in the context of rigorous research |

For peer review only

| | |
|---|---|
| Content of Care | C.3 - Antenatal prophylaxis with anti-D immunoglobulin in non-sensitized Rh-negative pregnant women at 28 and 34 weeks of gestation to prevent RhD alloimmunization is only recommended in the context of rigorous research |
| Experience of Care | D.1 - Ginger, chamomile, vitamin B6 and/or acupuncture are recommended for the relief of nausea in early pregnancy, based on a woman's preferences and available options |
| Experience of Care | D.2 - Advice on diet and lifestyle is recommended to prevent and relieve heartburn in pregnancy. Antacid preparations can be offered to women with troublesome symptoms that are not relieved by lifestyle modification |
| Experience of Care | D.3 - Magnesium, calcium or non-pharmacological treatment options can be used for the relief of leg cramps in pregnancy, based on a woman's preferences and available options |
| Experience of Care | D.4 - Regular exercise throughout pregnancy is recommended to prevent low back and pelvic pain. There are a number of different treatment options that can be used, such as physiotherapy, support belts and acupuncture, based on a woman's preferences and available options |
| Experience of Care | D.5 - Wheat bran or other fibre supplements can be used to relieve constipation in pregnancy if the condition fails to respond to dietary modification, based on a woman's preferences and available options |
| Experience of Care | D.6 - Non-pharmacological options, such as compression stockings, leg elevation and water immersion, can be used for the management of varicose veins and oedema in pregnancy, based on a woman's preferences and available options |
| Health Systems | E.2 - Midwife-led continuity-of-care models, in which a known midwife or small group of known midwives supports a woman throughout the antenatal, intrapartum and postnatal continuum, are recommended for pregnant women in settings with well functioning midwifery programmes |
| Health Systems | E.3 - Group antenatal care provided by qualified health-care professionals may be offered as an alternative to individual antenatal care for pregnant women in the context of rigorous research, depending on a woman's preferences and provided that the infrastructure and resources for delivery of group antenatal care are available |
| Health Systems | E.4.1 - The implementation of community mobilization through facilitated participatory learning and action (PLA) cycles with women's groups is recommended to improve maternal and newborn health, particularly in rural settings with low access to health services. Participatory women's groups represent an opportunity for women to discuss their needs during pregnancy, including barriers to reaching care, and to increase support to pregnant women |
| Health Systems | E.4.2 - Packages of interventions that include household and community mobilization and antenatal home visits are recommended to improve antenatal care utilization and perinatal health outcomes, particularly in rural settings with low access to health services |
| Health Systems | E.5.1 - Task shifting the promotion of health-related behaviours for maternal and newborn health to a broad range of cadres, including lay health workers, auxiliary nurses, nurses, midwives and doctors is recommended |
| Health Systems | E.5.2 - Task shifting the distribution of recommended nutritional supplements and intermittent preventive treatment in pregnancy (IPTp) for malaria prevention to a broad range of cadres, including auxiliary nurses, nurses, midwives and doctors is recommended |
| Content of Care | Fetal heart rate monitored † |

† Established good clinical practice within ANC

For peer review only

BMJ Open

# Developing measures for WHO recommendations on antenatal care for a positive pregnancy experience: a conceptual framework and scoping review

| | |
|---|---|
| Journal: | *BMJ Open* |
| Manuscript ID | bmjopen-2018-024130.R1 |
| Article Type: | Research |
| Date Submitted by the Author: | 11-Dec-2018 |
| Complete List of Authors: | Lattof, Samantha; World Health Organization, Department of Reproductive Health and Research<br>Tunçalp, Özge; World Health Organizations, Reproductive Health and Research<br>Moran, A; World Health Organization, Department of Maternal, Newborn, Child and Adolescent Health<br>Bucagu, Maurice; WHO, Department of Maternal, Newborn, Child and Adolescent Health<br>Chou , D ; World Health Organization, Department of Reproductive Health and Research<br>Diaz, Theresa; World Health Organizations, Department of Maternal, Newborn, Child and Adolescent Health<br>Gülmezoglu, A; World Health Organization, Department of Reproductive Health and Research |
| <b>Primary Subject Heading</b>: | Obstetrics and gynaecology |
| Secondary Subject Heading: | Evidence based practice, Global health, Public health, Reproductive medicine |
| Keywords: | Protocols & guidelines < HEALTH SERVICES ADMINISTRATION & MANAGEMENT, Quality in health care < HEALTH SERVICES ADMINISTRATION & MANAGEMENT, OBSTETRICS, PUBLIC HEALTH |
| | |

SCHOLARONE™
Manuscripts

# Title

Developing measures for WHO recommendations on antenatal care for a positive pregnancy experience: a conceptual framework and scoping review

# Authors

Corresponding author:

Samantha R. Lattof, PhD; Department of Reproductive Health and Research including UNDP/UNFPA/UNICEF/WHO/World Bank Special Programme of Research, Development and Research Training in Human Reproduction (HRP), World Health Organization, Avenue Appia 20, 1202 Geneva, Switzerland; lattof@post.harvard.edu, tel. +49 171 7549561

Co-authors:

Özge Tunçalp, MD; Department of Reproductive Health and Research including UNDP/UNFPA/UNICEF/WHO/World Bank Special Programme of Research, Development and Research Training in Human Reproduction (HRP), World Health Organization, Geneva, Switzerland

Allisyn C. Moran, PhD; Scientist, Department of Maternal, Newborn, Child and Adolescent Health, World Health Organization, Geneva, Switzerland

Maurice Bucagu, PhD; Medical Officer, Department of Maternal, Newborn, Child and Adolescent Health, World Health Organization, Geneva, Switzerland

For peer review only

Doris Chou, MD; Medical Officer, Department of Reproductive Health and Research including UNDP/UNFPA/UNICEF/WHO/World Bank Special Programme of Research, Development and Research Training in Human Reproduction (HRP), World Health Organization, Geneva, Switzerland

Theresa Diaz, MD; Coordinator, Department of Maternal, Newborn, Child and Adolescent Health, World Health Organization, Geneva, Switzerland

A. Metin Gülmezoglu, MD; Coordinator, Department of Reproductive Health and Research including UNDP/UNFPA/UNICEF/WHO/World Bank Special Programme of Research, Development and Research Training in Human Reproduction (HRP), World Health Organization, Geneva, Switzerland

## Keywords

prenatal care; pregnancy; guideline; quality indicators; public health

## Word count

3,916 words

## Abstract

*Objectives*

In response to the newest World Health Organization (WHO) recommendations on routine antenatal care (ANC) for pregnant women and adolescent girls, this paper identifies the literature on existing ANC measures, presents a conceptual framework for quality ANC, maps existing measures to specific WHO recommendations, identifies gaps where new

For peer review only

measures are needed to monitor the implementation and impact of routine ANC, and prioritizes measures for capture.

*Methods*

We conducted searches in four databases and five websites. Searches and application of inclusion/exclusion criteria followed the PRISMA flow approach for scoping reviews. Data were extracted on measure information, methodology, methodological work, and implementation. We adapted and refined a conceptual framework for routine ANC based on these measures.

*Results*

This scoping review uncovered 58 resources describing 46 existing measures that align with WHO recommendations and good clinical practices for ANC. Of the 42 WHO-recommended ANC interventions and four good clinical practices included in this scoping review, only 14 WHO-recommended interventions and three established good clinical practices could potentially be measured immediately using existing measures. Recommendations addressing the integration of ANC with allied fields are more likely to have existing measures than recommendations that focus on maternal health. When mapped to our conceptual framework, existing measures prioritize content of care and health systems; measures for girls' and women's experiences of care are notably lacking. Existing data sources for non-existent measures are currently limited.

*Conclusion*

Our research updates prior efforts to develop comprehensive measures of quality ANC and raises awareness of the need to better assess experiences of ANC. Given the inadequate number and distribution of existing ANC measures across the quality of care conceptual framework domains, new standardized measures are required to assess quality of routine ANC. Girls' and women's voices deserve greater acknowledgment when measuring the quality and delivery of ANC.

For peer review only

## Strengths and limitations of this study

- This research updates prior efforts to develop comprehensive measures of quality antenatal care and raises awareness of the need to better assess girls' and women's experiences of care.
- We searched a range of databases and websites with different disciplinary foci and solicited expert recommendations to maximize heterogeneity in the material included in this scoping review.
- To minimize bias in the scoping review and to generate reliable findings from which to draw conclusions about substantive measures for routine antenatal care, we employed the PRISMA flow approach for scoping reviews.
- Methodological limitations may have resulted in missed relevant measures, specifically relevant measures in languages other than English, as well as measures in grey literature and in-service reports, where it was unfeasible to search these infinite sources thoroughly.

For peer review only

# Introduction

In 2016, the World Health Organization (WHO) released its comprehensive recommendations on routine antenatal care (ANC) for pregnant women and adolescent girls.[1] A systematic review of women's views shaped the scope of this guideline, since women's experiences of ANC are key to transforming ANC and to creating the foundation for healthy motherhood. At its core, this guideline aims "to provide pregnant women with respectful, individualized, person-centred care at every contact, with implementation of effective clinical practices (interventions and tests), and provision of relevant and timely information, and psychosocial and emotional support, by practitioners with good clinical and interpersonal skills within a well functioning health system," (p. 105-06).[1] Monitoring the implementation and impact of routine ANC, as described in the guideline, requires moving beyond the global benchmark indicator of four or more ANC visits to include measures focusing on ANC content and care processes.[2]

The WHO has prioritized the development of indicators in recent years, releasing guidelines and measures for core health,[3] essential interventions,[4] preventable maternal mortality,[5] preventable newborn deaths and stillbirths,[6] and quality of maternal and newborn care in health facilities;[7] however, limited ANC measures appear throughout these documents. Other efforts to develop comprehensive measures of quality ANC have resulted in a narrow range of measures that emphasize clinical practices like hospitalization for uncontrolled diabetes[8] or indicators for service utilization, screening, and interventions.[9] Measuring women and girls' experience of respectful, individualized, person-centred ANC; the provision of relevant and timely information; and psychosocial and emotional support have received far less attention.

Variation in data quality, data (in)availability, and data heterogeneity (e.g. data sources, types of data, data uses) also challenge existing efforts to assess ANC coverage, content, and quality.[10] In recognition of these challenges, researchers have called for the collection of high-quality ANC

For peer review only - http://bmjopen.bmj.com/site/about/guidelines.xhtml

data,[11] the measurement of content of care as well as the number of contacts,[2 5] the establishment of relevant indicators to support data collection for developing appropriate policy for vulnerable populations,[12] and greater support for the implementation of digital registries and data platforms that overcome gaps in the availability of existing information from publicly available household and facility surveys.[4] Country programmes have also called for better data so that decision-makers and advocates have the necessary evidence to improve maternal and newborn survival and health.[13]

With the launch of the new guideline and as countries reassess their ANC programmes accordingly, there is now an urgent need to develop and implement appropriate measures for national and global monitoring of routine ANC. This paper presents a scoping review that aims to (1) identify the literature on ANC measures as they relate to the WHO guideline[1] and (2) map existing measures to specific recommendations in the guideline. To inform the development of a monitoring framework for implementation of the WHO ANC model and ground the model in a conceptual framework for quality ANC, our paper also identifies gaps where new measures are needed for implementation and prioritizes measures for capture.

For peer review only

# Methods

## Conceptual framework

Within the context of maternal and newborn health, the WHO defines quality of care as "the degree to which maternal and newborn health services (for individuals and populations) increase the likelihood of timely, appropriate care for the purpose of achieving desired outcomes that are both consistent with current professional knowledge and take into account the preferences and aspirations of individual women and their families," (p. 15).[7] Based on the guideline and supplemented by the findings from the scoping review, we customized the WHO's framework for quality maternal and newborn health care[7] focusing on routine ANC. We designed the framework by grouping similar measures for assessing, improving, and monitoring ANC. Needed ANC measures, both existing and non-existing, are mapped by recommendation to the framework's domains; thus, the framework only includes health system building blocks that align with the new ANC recommendations. In presenting the results, we use the conceptual framework's three domains to group findings from the scoping review.

## Search strategy and screening process

After first testing search terms for their appropriateness for this scoping review (e.g. measure∗), we conducted searches of the final search terms ((antenatal OR prenatal) AND (indicator∗)) in four databases (PubMed, ISI Web of Science, ScienceDirect, Popline). The term "measure∗" was too broad in the pilot searches and necessitated that we restrict the searches to "indicator∗." We realize, however, that the term "indicator" or "indicators" is closely related to monitoring and evaluation. This scoping review is not intended as a list of indicators for monitoring, so we use the term "measure" rather than "indicator" in the manuscript to emphasize that this scoping review examines how ANC services can be measured according to the guideline. In the next phase, these measures will be prioritized to inform the common indicators within WHO ANC monitoring framework.

For peer review only - http://bmjopen.bmj.com/site/about/guidelines.xhtml

Table 1 provides specifications of the items scoped in this review. The search included pregnant women and girls and any measured intervention matching one of the 42 recommended interventions in the publication *WHO recommendations on antenatal care for a positive pregnancy experience* or one of the four specified practices of good clinical practice during ANC. Searches were constrained by species (human) and language (English) when databases permitted and adapted to the particulars (e.g. wildcards, truncations, capacity for complex searches) of each electronic database. Searches in ScienceDirect were limited to title, abstract, and keywords. In addition, we thematically searched five additional websites (WHO, MEASURE Evaluation, The Demographic and Health Survey (DHS) Program, UNICEF Multiple Indicator Cluster Surveys (MICS), Countdown to 2030) for webpages related to measures and/or to maternal health, in order to maximize the coverage of non-peer-reviewed materials.

For peer review only

**Table 1: PICOTS criteria used in the scoping review**

| PICOTS | Inclusion criteria |
|---|---|
| Population | Pregnant girls and women |
| Intervention | A measured intervention related to one of the 42 recommended interventions in the publication *WHO recommendations on antenatal care for a positive pregnancy experience*[1] or one of the four specified practices of good clinical practice during ANC (i.e. counselling on birth preparedness, counselling on family planning, monitoring of fetal heart rate, and monitoring of blood pressure) |
| Control | None |
| Outcomes | - Reports a measure that aligns with at least one of the recommendations in the publication *WHO recommendations on antenatal care for a positive pregnancy experience*[1] or one of the four specified good clinical practices for ANC<br>- Defines the measure<br>- Explains the methodology for measurement |
| Timeframe | Duration of antenatal care |
| Setting | Any |

SL conducted the searches and subsequent application of inclusion/exclusion criteria according to the PRISMA (Preferred Reporting Items for Systematic Reviews and Meta-Analyses) flow approach for scoping reviews. For quality

For peer review only

assurance purposes, SL and ÖT independently screened the first 50 items managed in EndNote, compared results, and discussed and resolved any differences in understanding of the inclusion/exclusion criteria. The criteria were further elaborated where necessary on the basis of their independent screening. SL screened items identified by the search on the basis of title and abstract (TIAB). When inclusion or exclusion could not be determined on the basis of TIAB, SL screened full texts. At the full text screening stage, ÖT and AM independently screened any items that SL considered borderline or problematic. We report information on this scoping review using the PRISMA extension checklist for scoping reviews.[14]

**Inclusion and exclusion criteria**

This review focused on measures of ANC for a positive pregnancy experience based on the WHO's most recent guideline.[1] The guideline defines a positive pregnancy experience as "maintaining physical and sociocultural normality, maintaining a healthy pregnancy for mother and baby (including preventing or treating risks, illness and death), having an effective transition to positive labour and birth, and achieving positive motherhood (including maternal self-esteem, competence and autonomy)," (p. lx).[1] As part of the guideline development process, the Guideline Development Group did not evaluate evidence or make recommendations for good clinical practices, as they are considered to be essential components of ANC.[1] Since these established good clinical practices should be implemented alongside the guideline's recommendations and are part of the WHO ANC model, this scoping review also considered measures for four established practices of good ANC for inclusion. These established good clinical practices included: counselling on birth preparedness, counselling on family planning, monitoring of fetal heart rate, and monitoring of blood pressure.[1]

Resources on pregnant girls and women in any setting were considered. Peer-reviewed journal articles and (non-peer-reviewed) grey literature were eligible for inclusion. Potentially eligible resources included journal articles and un/published information from governments and other agencies, whether

For peer review only

available in print or online, published in English. Quality was not assessed and was not a criterion for inclusion. Multiple references based on the same measure/recommendation were not excluded, as they might contain useful information on current methodological work and experiences implementing the measure.

For inclusion, resources must have:

- Used a measure that aligns with at least one of the recommendations in the publication *WHO recommendations on antenatal care for a positive pregnancy experience*[1] or one of the four specified clinical practices of good ANC
- Defined the measure
- Explained the methodology for measurement
- Been published in English
- Been published between 1 January 2005 and 26 August 2017

Unless resources conducting secondary analyses of existing global datasets (e.g. MICS, DHS, DHS Service Provision Assessments) introduced new measures or indices based on the data or provided additional information on the measure's methodology, these articles were excluded. When resources noted that they used a common global measure (e.g. a measure from Countdown to 2015), then we excluded these resources in favour of the primary resource (e.g. the Countdown to 2015 report that developed or approved the measure[15]). This scoping review excludes the guideline's seven recommendations for which the Guideline Development Group recommended no intervention (A.1.4, A.6, A.7, A.8, A.9, B.2.3, and B.2.5).[1]

**Data extraction and analysis**

Since the focus of this research is on specific measures rather than specific studies, individual measure data were compiled from multiple studies. Data were extracted on measure information (e.g. definition, numerator, denominator), methodology (e.g. method of measurement, measurement frequency, data sources), published or current methodological work, and

For peer review only

experiences implementing the measure. We refined the conceptual framework for routine ANC based on these measures. Results are presented in a narrative format accompanied by tables of identified measurement areas for ANC guideline monitoring and evaluation.

### Patient and public involvement

This scoping review did not involve patients. However, the need for this scoping review was initiated by the WHO ANC guideline for which multiple qualitative evidence syntheses that explored women's needs and perspectives during ANC were conducted. As part of the guideline's development, the panel included a patient representative and members representing women.

## Results

### WHO quality of care framework focusing on routine antenatal care

Building on the WHO framework for quality maternal and newborn health care,[7] we mapped the existing and non-existent ANC measures across three dimensions by which to measure recommendations for routine ANC (Figure 1): health systems (existing measures = 7, non-existent measures = 6), content of care (existing measures = 39, non-existent measures = 16), and women's experience of care (existing measures = 0, non-existent measures = 6). These three domains form the core of the conceptual framework and influence outcomes at the individual and facility levels. This framework is intended to assist health care providers, managers, and policymakers to better understand and improve the quality of routine ANC for a positive pregnancy experience. It can also be used to assess health system characteristics required to deliver quality ANC.

The health system provides the structure by which factors such as service delivery models and health workforce organization impact quality of ANC processes. The content of ANC and women's experiences of ANC form the

two pillars of quality ANC and are reliant upon the availability of human resources and physical resources. Content of care includes interventions related to maternal and fetal assessment and management, nutritional interventions, infectious disease testing and management, and counselling and information sharing. As part of the WHO quality of care framework, women's experience of care includes effective communication, respect and dignity and emotional support. When ANC recommendations were mapped to women's experience of care, assessment and management of physical symptoms were the only ones.

**Scoping review results**

Figure 2 illustrates the search and screening processes. The scoping generated a total of 58 resources that described 46 existing measures (Supplementary Material, Table A1). These measures align with 14 recommendations for a positive pregnancy experience and three established good clinical practices. Whilst the scoping review identified measures that were common across topic areas and reported by multiple authors (Supplementary Material, Table A1), it also identified unique measures. Multiple unique measures exist for recommendations addressing iron and folic acid supplements (n=7), HIV and syphilis (n=7), tetanus toxoid vaccination (n=6), intermittent preventive treatment of malaria in pregnancy (IPTp) (n=4), intimate partner violence (n=2), and tobacco (n=2). Nine additional measures exist for three established good clinical practices within ANC: monitoring of blood pressure (n=5), counselling about birth preparedness (n=2), and counselling about family planning (n=2). Twenty-seven of the guideline's recommendations and one established good clinical practice lack existing measures (Supplementary Material, Table A2). Table 2 sorts these existing and missing measurement areas by topic. Proposed measurement areas for recommendations in the context of research are summarized in Table 3.

For peer review only

**Table 2: Identified measurement areas for ANC guideline monitoring and evaluation by topic areas**

| Topic area | What to be measured? | Measures identified by the scoping review | Scoping review sources |
|---|---|---|---|
| **Health systems** | | | |
| Service delivery models | Antenatal care contacts (eight or more) | x | 16 |
| | Pregnant women carrying their own case notes | x | 17 |
| | Service-specific availability and readiness: midwife-led continuity-of-care⁎ | | |
| | Timing of first antenatal care visit | x | 16 18-29 |
| Health workforce organization | Health worker density and distribution⁎ | x | 30 |
| | Health units with at least one service provider trained to care for and refer sexual and gender-based violence survivors⁎ | x | 31-33 |
| | Policy on task shifting for antenatal care (counselling and provision of selected interventions) | | |
| Community engagement | Communities offering facilitated participatory learning and action cycles with women's groups to improve maternal and newborn health⁎ | | |
| **Content of care** | | | |

| Topic area | What to be measured? | Measures identified by the scoping review | Scoping review sources |
|---|---|---|---|
| Maternal and fetal assessment and management | Assessment for tobacco use and second-hand smoke exposure | x | 4 26 |
| | Assessment for use of alcohol and other substances | | |
| | Ultrasound scan before 24 weeks | | |
| | On-site haemoglobin testing for anaemia* | | |
| | On-site testing for asymptomatic bacteriuria* | | |
| | Treatment for asymptomatic bacteriuria | | |
| | Symphysis-fundal height measurement* | | |
| | Monitoring of fetal heart rate§ | | |
| | Monitoring of blood pressure§ | x | 20 34-40 |
| Nutritional interventions | Iron and folic acid supplementation* | x | 4 16 33 35 39 41-46 |
| | Availability of balanced energy and protein dietary supplementation | | |
| | Calcium supplementation* | | |
| | Vitamin A supplementation coverage* | | |
| | Caffeine intake information | | |
| Infectious disease testing and management | Pregnant women counselled and tested for HIV and know their results | x | 4 17 33 35 44 47-53 |
| | Testing for syphilis | x | 33 35 49 54 |

| Topic area | What to be measured? | Measures identified by the scoping review | Scoping review sources |
|---|---|---|---|
| | | | 55 |
| | Treatment for helminths⁎ | x | 33 35 42 |
| | Newborns protected at birth from tetanus | x | 15 17 33 35 39 41-45 56 |
| | Intermittent preventive therapy for malaria⁎ | x | 30 39 44 57 58 |
| | Testing for tuberculosis⁎ | | |
| | Antiretroviral pre-exposure prophylaxis to prevent HIV infection⁎ | x | 59 |
| Counselling and information sharing | Counselling on diet and exercise in pregnancy⁎ | | |
| | Counselling on birth preparedness§ | x | 35 36 60 |
| | Counselling on family planning§ | x | 35 44 |
| **Experience of care** | | | |
| Management of physiological symptoms | Information and treatment for common physiological symptoms (e.g. leg cramps, constipation, nausea) | | |

⁎ Measurement area is for an ANC recommendation that is unique to specific contexts (e.g. undernourished populations, high-prevalence settings, malaria-endemic areas).
§ Measurement area is for a good clinical practice within ANC.

Note: This table condenses measures by intervention type. For example, “iron and folic acid supplementation” includes all measures for recommendations A.2.1 (daily oral iron and folic acid supplementation) and A.2.2 (intermittent oral iron and folic acid supplementation).

For peer review only

**Table 3: Identified measurement areas for monitoring ANC recommendations in the context of research by topic areas**

| Topic area | What to be measured? | Measures identified by the scoping review | Scoping review sources |
|---|---|---|---|
| **Health systems** | | | |
| Service delivery models | Service-specific availability and readiness: group antenatal care | | |
| **Content of care** | | | |
| Maternal and fetal assessment and management | Prophylaxis for recurrent urinary tract infections | x | 61 |
| | Prophylaxis with anti-D immunoglobulin in non-sensitized Rh-negative pregnant women | | |
| | Daily fetal movement counting | x | 62 63 |
| | Zinc supplementation | | |

Existing measures are primarily implemented within the conceptual framework's content of care domain followed by the health systems domain. Existing measures for women's experience of ANC in terms of communication, support and respect are sparse and infrequently collected. Our scoping review revealed no existing measures captured during ANC for recommendations involving women's experiences of care. The existing measures could permit immediate measurement of 14 ANC recommendations in the WHO guideline using currently available data. Three of the 14 recommendations (B.1.7, C.6, and E.7)[1] are perfectly aligned with existing measures. The 11 remaining recommendations have subtle gaps or

For peer review only

discrepancies with the existing measures and would require minimal modification or disaggregation to be relevant. Furthermore, some of the measures that are currently used may need to be revised based on the new recommendations (e.g., number of ANC contacts).

Most measures currently being implemented have consistent definitions; however, ten measures exhibit variation in their definitions and measurement (i.e. numerator and denominator). The measure “iron and folic acid supplements for pregnant women (%),” for instance, has multiple definitions that vary by supplementation duration (unspecified, 90+ days, at least six months), how women obtained the supplements (received, received or bought, given), and supplement dosage (unspecified, any, amounts in accordance with recommended protocols).[4,16,33,42] The measure “first antenatal care visit in the first trimester” presents even greater variation in measurement with researchers defining the first trimester as: first trimester,[24,28] <12 weeks,[22,27] ≤12 weeks,[23] <13 weeks,[21] by 13 weeks 0 days,[25] and <14 weeks.[29]

Several proposed measures (Table 2) are for similar recommendations and could potentially be streamlined. For instance, recommendations E.5.1 and E.5.2 (task shifting components of ANC delivery) are quite similar; E.5.1 focuses on a broad range of activities whereas E.5.2 focuses solely on the distribution of nutritional supplements and IPTp for malaria prevention.[1] A single new health system measure "policy on task shifting for antenatal care" could collect data on both recommendations and then be disaggregated to permit monitoring the two recommendations separately. Among the recommendations lacking existing measures (Supplementary Material, Table A2), these non-existent measures relate to interventions involving health systems (n=6), nutrition (n=7), maternal and fetal assessment (n=6), common physiological symptoms (n=6), preventative measures (n=2), and good clinical practice (n=1).

Sources of existing data for these non-existent measures are currently limited. Monitoring the guideline’s components of quality ANC that currently

For peer review only

lack existing measures would thus require the development of new measures as well as improved data sources. Data for eight proposed measures could be obtained relatively quickly from women's individual ANC records (case notes), and health policy guidelines/directives. Data from existing clinical records could be difficult to obtain, as these data are often not linked for each ANC visit. Population-based surveys (e.g. DHS, MICS) do not currently capture the required data for these recommendations, necessitating the adaptation of existing tools to capture new measures. MICS, for example, currently asks whether women have a card or other document with their immunisations listed.[64] If this question (MN7) could be amended to include ANC case notes, it would permit the monitoring of recommendation E.1 (women-held case notes).[1]

## Discussion

This scoping review uncovered 46 existing measures that align with the WHO recommendations on routine ANC for pregnant women and adolescent girls.[1] Of the 42 recommended ANC interventions and four good clinical practices included in this scoping review, we found that only 14 WHO recommended interventions and three established good clinical practices could potentially be measured immediately using existing measures. Recommendations addressing the integration of ANC with allied fields (e.g. HIV, malaria, nutrition) are more likely to have existing measures than recommendations that focus solely on maternal health, especially recommendations related to organization of care (e.g. midwife-led continuity-of-care, group ANC).

These existing ANC measures prioritize content of care followed by health systems. Existing ANC measures largely overlook girls' and women's experiences of care, the third dimension at the core of the quality of care framework for routine ANC. Research highlights that experience of care is an integral part of quality of care[65] and respectful care as described by women. In fact, experience of care overlaps with almost all of the quality of care domains described above.[66] Designing health care services with a

For peer review only

women-centred approach is critical for utilization, quality, and impact of these services; therefore, girls' and women's voices deserve greater acknowledgment when measuring the quality and delivery of ANC.[67]

Like other researchers, we found that measures to assess ANC coverage and content are restricted by data heterogeneity, variation in data quality, and data availability.[4 10] Measures must be standardized in definition, measurement, and level of data collection and usage if they are to permit meaningful comparability across settings and over time. Monitoring the implementation and impact of routine ANC, as described in the WHO guideline,[1] is one of multiple efforts that would benefit from standardizing and strengthening the development of ANC measures. Moreover, improved measures would assist researchers and programme implementers in assessing the content and quality of ANC,[68] tracking effective implementation of policies addressing social determinants of maternal health,[69] revealing ANC implementation bottlenecks,[54] assessing equity of ANC programme coverage and utilization,[41] and evaluating the effectiveness of new innovations like mHealth platforms for delivering maternal and child health services.[70]

Our research updates prior efforts to develop comprehensive measures of quality ANC,[8 9 71] and it raises awareness of the need to better assess women's experiences of care. New tools currently under development by the WHO-convened Maternal Morbidity Working Group recognize the importance of women's experiences in measuring maternal morbidity and improving the health care of pregnant women.[72 73] In addition to creating better tools to evaluate quality ANC as experienced by girls and women, researchers must also improve human capacity for correct, timely documentation of ANC data. Evidence from Brazil revealed a discrepancy between data from antenatal booklets and data recorded in the SISPRENATAL (System to Accompany the Prenatal and Birth Humanization Program) software.[74] Researchers attributed the discrepancy between data sources to the timing of data collection. Antenatal booklets, in which data is recorded during women's visits, produced more reliable data than the SISPRENATAL software, in

For peer review only

which health unit employees who were uninvolved in women's visits completed worksheets after women's visits.[74] These findings reinforce the need for reliable, high-quality data sources to properly monitor and evaluate quality ANC. Furthermore, greater attention is needed to psychosocial and emotional support during pregnancy, starting with further development and refinement of these terms as they apply within ANC.

Methodological limitations may have resulted in missed relevant measures, specifically relevant measures in languages other than English, as well as measures in grey literature and in-service reports, where it was unfeasible to search these infinite sources thoroughly. Finally, we were unable to locate the full text for nine items for screening. It is possible that these items may have been eligible for inclusion. To minimize bias in the scoping review and to generate reliable findings from which to draw conclusions about substantive measures for routine ANC, we employed the PRISMA flow approach for scoping reviews. To mitigate possible publication bias, this search strategy incorporated non-peer-reviewed and unpublished papers and reports. We searched a range of databases and websites with different disciplinary foci and solicited expert recommendations to maximize heterogeneity in the material included in this scoping review.

## Conclusion

Given the inadequate number and distribution of existing ANC measures across the quality of care framework domains, new standardized measures are required to assess quality of routine ANC. The WHO's new ANC model emphasizes integrated delivery of all the care women need during the antenatal period, so the measurement of ANC should also be integrated. Developing these measures will require strong partnerships and greater integration with allied fields so that the measures are implemented correctly.

Our scoping review and conceptual framework will inform the monitoring framework for ANC that will incorporate a prioritized list of measures to better assist countries and health facilities in consistently monitoring and

For peer review only

assessing progress towards improved ANC. Furthermore, partners in South Africa have been implementing the new ANC model at scale since April 2017. In collaboration with the South African Medical Research Council, WHO is analysing the effects of implementing this model on workload, detecting hypertension, and perinatal mortality with the associated set of indicators that will further inform the development of this monitoring framework. This framework will determine the feasibility and utility of data collection as well as the indicators' place within the global monitoring efforts undertaken by initiatives such as Ending Preventable Maternal Mortality. Development and testing of new indicators, especially for critical areas such as experience of care, will be part of the proposed research agenda.

## Acknowledgements

We wish to thank Juan Pablo Peña-Rosas at the WHO for his contributions to the antenatal guideline on which this work is based.

## Contributors

ÖT, ACM, and SRL conceived and planned the study with input from DC, TD, MB and AMG. SRL conducted the searches and subsequent application of inclusion/exclusion criteria. SRL and ÖT independently screened the first 50 items to ensure consistency in the inclusion/exclusion criteria. SRL inputted the data. ÖT, ACM, SRL, and MB contributed to the interpretation of the findings. SRL wrote the first draft of the manuscript, and all authors contributed to subsequent revisions.

## Funding

This work was supported by the United States Agency for International Development.

For peer review only

## Competing interests

We declare no competing interests.

## Data sharing statement

The review protocol and data extraction workbook are available upon request from the WHO.

For peer review only

## References

1. WHO. WHO recommendations on antenatal care for a positive pregnancy experience. Geneva: WHO, 2016a:[152] p.

2. Hodgins S, D'Agostino A. The quality-coverage gap in antenatal care: toward better measurement of effective coverage. *Global Health, Science and Practice* 2014;2(2):173-81. doi: 10.9745/GHSP-D-13-00176

3. WHO. Global Reference List of 100 Core Health Indicators (plus health-related SDGs). Geneva: World Health Organization, 2018.

4. Flenady V, Wojcieszek AM, Fjeldheim I, et al. eRegistries: indicators for the WHO Essential Interventions for reproductive, maternal, newborn and child health. *Bmc Pregnancy and Childbirth* 2016;16 doi: 10.1186/s12884-016-1049-y

5. Moran AC, Jolivet RR, Chou D, et al. A common monitoring framework for ending preventable maternal mortality, 2015-2030: phase I of a multi-step process. *BMC Pregnancy and Childbirth* 2016;16(1):250. doi: 10.1186/s12884-016-1035-4

6. Moxon SG, Ruysen H, Kerber KJ, et al. Count every newborn; a measurement improvement roadmap for coverage data. *BMC pregnancy and childbirth* 2015;15 Suppl 2:S8. doi: 10.1186/1471-2393-15-s2-s8 [published Online First: 2015/09/24]

7. WHO. Standards for improving quality of maternal and newborn care in health facilities. Geneva: WHO, 2016b.

8. Korst LM, Gregory KD, Lu MC, et al. A Framework for the Development of maternal quality of care indicators. *Maternal and child health journal* 2005;9(3):317-41. doi: 10.1007/s10995-005-0001-y [published Online First: 2005/09/15]

9. Bollini P, Quack-Lotscher K. Guidelines-based indicators to measure quality of antenatal care. *Journal of evaluation in clinical practice* 2013;19(6):1060-6. doi: 10.1111/jep.12027 [published Online First: 2013/03/27]

10. Moller A-B, Petzold M, Chou D, et al. Early antenatal care visit: a systematic analysis of regional and global levels and trends of coverage from 1990 to 2013. *The Lancet Global Health*;5(10):e977-e83. doi: 10.1016/S2214-109X(17)30325-X

11. Raynes-Greenow C. Gaps and challenges underpinning the first analysis of global coverage of early antenatal care. *The Lancet Global Health* 2017;5(10):e949-e50. doi: 10.1016/S2214-109X(17)30346-7

12. Steenkamp M, Bar-Zeev S, Rumbold A, et al. Pragmatic indicators for remote Aboriginal maternal and infant health care: why it matters and where to start. *Australian and New Zealand journal of public health* 2010;34 Suppl 1:S5-8. doi: 10.1111/j.1753-6405.2010.00545.x [published Online First: 2010/07/21]

13. Evidence for Action. MamaYe Evidence for Action Stories of Change: Selected Case Studies. London: E4A, 2015.

14. Tricco AC, Lillie E, Zarin W, et al. Prisma extension for scoping reviews (prisma-scr): Checklist and explanation. *Annals of Internal Medicine* 2018;169(7):467-73. doi: 10.7326/M18-0850

For peer review only

15. Countdown to 2015. Building a Future for Women and Children: The 2012 Report. Washington, D.C.: World Health Organization and UNICEF, 2012.

16. MoNITOR. Maternal and Newborn Indicator Mapping. Geneva: World Health Organization, 2017.

17. Bali K, Wheeler R, Davoodnia R, et al. Partnership for Maternal and Neonatal Health - West Pokot District Child Survival and Health Program. New York: Doctors of the World, 2007.

18. Doubova SV, Perez-Cuevas R, Ortiz-Panozo E, et al. Evaluation of the quality of antenatal care using electronic health record information in family medicine clinics of Mexico City. *BMC pregnancy and childbirth* 2014;14:168. doi: 10.1186/1471-2393-14-168 [published Online First: 2014/06/03]

19. Economic Growth Center (EGC) at Yale University, Institute of Statistical SERIaUoG, . Ghana Socio-economic Panel Survey: Household Instrument Wave One (2009) Part B. Living Standards Measurement Study Questionnaire. Legon, Ghana: University of Ghana, 2009.

20. Ejigu T, Woldie M, Kifle Y. Quality of antenatal care services at public health facilities of Bahir-Dar special zone, Northwest Ethiopia. *BMC health services research* 2013;13:443. doi: 10.1186/1472-6963-13-443 [published Online First: 2013/10/29]

21. Kildea S, Gao Y, Rolfe M, et al. Remote links: Redesigning maternity care for Aboriginal women from remote communities in Northern Australia - A comparative cohort study. *Midwifery* 2016;34:47-57. doi: 10.1016/j.midw.2016.01.009 [published Online First: 2016/03/15]

22. Liu L, Li M, Yang L, et al. Measuring coverage in MNCH: a validation study linking population survey derived coverage to maternal, newborn, and child health care records in rural China. *PloS one* 2013;8(5):e60762. doi: 10.1371/journal.pone.0060762 [published Online First: 2013/05/15]

For peer review only

23. Lu J, Shen JJ, Chen G, et al. Regional disparities in prenatal care services in rural China. *Asia-Pacific journal of public health* 2011;23(5):682-9. doi: 10.1177/1010539511418356 [published Online First: 2011/08/20]

24. Mbuagbaw L, Medley N, Darzi AJ, et al. Health system and community level interventions for improving antenatal care coverage and health outcomes. *The Cochrane database of systematic reviews* 2015(12):Cd010994. doi: 10.1002/14651858.CD010994.pub2 [published Online First: 2015/12/02]

25. National Institute for Health and Care Excellence. NICE menu indicator guidance: Indicator CCG30. London: NICE, 2016.

26. Reeve C, Banfield S, Thomas A, et al. Community outreach midwifery-led model improves antenatal access in a disadvantaged population. *The Australian journal of rural health* 2016;24(3):200-6. doi: 10.1111/ajr.12249 [published Online First: 2015/09/24]

27. Rumbold AR, Bailie RS, Si D, et al. Delivery of maternal health care in Indigenous primary care services: baseline data for an ongoing quality improvement initiative. *BMC pregnancy and childbirth* 2011;11:16. doi: 10.1186/1471-2393-11-16 [published Online First: 2011/03/10]

28. Servan-Mori E, Contreras-Loya D, Gomez-Dantes O, et al. Use of performance metrics for the measurement of universal coverage for maternal care in Mexico. *Health Policy and Planning* 2017;32(5):625-33. doi: 10.1093/heapol/czw161

29. Yucel C, Taskin L, Low LK. Validity and reliability of the Turkish version of the Optimality Index-US (OI-US) to assess maternity care outcomes. *Midwifery* 2015;31(12):1135-42. doi: 10.1016/j.midw.2015.08.003 [published Online First: 2015/08/25]

30. WHO. Global Reference List of 100 Core Health Indicators. Geneva: World Health Organization, 2015.

For peer review only

31. Bloom S. Violence against Women and Girls: A Compendium of Monitoring and Evaluation Indicators. Chapel Hill, North Carolina, USA: MEASURE Evaluation, 2008.

32. Bunn MY, Higa NA, Parker WJ, et al. Domestic violence screening in pregnancy. *Hawaii medical journal* 2009;68(10):240-2. [published Online First: 2009/12/17]

33. MEASURE Evaluation. Summary List of Indicators Chapel Hill, North Carolina, USA: MEASURE Evaluation; 2017 [Available from: https://www.measureevaluation.org/prh/rh_indicators/indicator-summary accessed 27 Aug 2017.

34. Beeckman K, Frith L, Gottfreosdottir H, et al. Measuring antenatal care use in Europe: is the content and timing of care in pregnancy tool applicable? *International Journal of Public Health* 2017;62(5):583-90. doi: 10.1007/s00038-017-0959-4

35. Dwivedi V, Drake M, Rawlins B, et al. A Review of the Maternal and Newborn Health Content of National Health Management Information Systems in 13 Countries in Sub-Saharan Africa and South Asia. Washington, D.C.: Maternal and Child Health Integrated Program, 2014.

36. Marchant T, Tilley-Gyado RD, Tessema T, et al. Adding Content to Contacts: Measurement of High Quality Contacts for Maternal and Newborn Health in Ethiopia, North East Nigeria, and Uttar Pradesh, India. *PloS one* 2015;10(5) doi: 10.1371/journal.pone.0126840

37. McNabb M, Chukwu E, Ojo O, et al. Assessment of the quality of antenatal care services provided by health workers using a mobile phone decision support application in northern Nigeria: a pre/post-intervention study. *PloS one* 2015;10(5):e0123940. doi: 10.1371/journal.pone.0123940 [published Online First: 2015/05/06]

38. Melo EC, de Oliveira RR, Mathias TAD. Factors associated with the quality of prenatal care: an approach to premature birth. *Revista Da*

For peer review only

*Escola De Enfermagem Da Usp* 2015;49(4):540-48. doi: 10.1590/s0080-623420150000400002

39. Mwaniki MK, Vaid S, Chome IM, et al. Improving service uptake and quality of care of integrated maternal health services: the Kenya Kwale District improvement collaborative. *BMC health services research* 2014;14:416. doi: 10.1186/1472-6963-14-416 [published Online First: 2014/09/23]

40. Wilunda C, Tanaka S, Putoto G, et al. Evaluation of a maternal health care project in South West Shoa Zone, Ethiopia: before-and-after comparison. *Reproductive health* 2016;13(1):95. doi: 10.1186/s12978-016-0213-1 [published Online First: 2016/08/21]

41. Baqui AH, Rosecrans AM, Williams EK, et al. NGO facilitation of a government community-based maternal and neonatal health programme in rural India: improvements in equity. *Health Policy and Planning* 2008;23(4):234-43. doi: 10.1093/heapol/czn012

42. Hodgins S, McPherson R, Suvedi BK, et al. Testing a scalable community-based approach to improve maternal and neonatal health in rural Nepal. *Journal of perinatology : official journal of the California Perinatal Association* 2010;30(6):388-95. doi: 10.1038/jp.2009.181 [published Online First: 2009/11/13]

43. Kavitha N. Are Young Mothers in India Deprived of Maternal Health Care Services? A Comparative Study of Urban and Rural Areas. *Journal of Health Management* 2015;17(2):204-20. doi: http://dx.doi.org/10.1177/0972063415575809

44. Keating J. Nigeria Baseline Health Facility Survey, 2005: [Chapel Hill, North Carolina], University of North Carolina at Chapel Hill, Carolina Population Center [CPC], MEASURE Evaluation, 2006 Oct., 2006:[78] p.

45. Marin GH, Silberman M, Uriarte A, et al. Healthcare model based on personalised attention: impact on maternal mortality and health system

For peer review only

quality. *Quality in primary care* 2011;19(5):311-6. [published Online First: 2011/12/22]

46. Ogundipe O, Hoyo C, Ostbye T, et al. Factors associated with prenatal folic acid and iron supplementation among 21,889 pregnant women in Northern Tanzania: a cross-sectional hospital-based study. *BMC public health* 2012;12:481. doi: 10.1186/1471-2458-12-481 [published Online First: 2012/06/28]

47. Chabikuli ON, Gwarzo U, Olufunso A, et al. Closing the prevention of mother-to-child transmission gap in Nigeria: an evaluation of service improvement intervention in Nigeria. *South African Family Practice* 2013;55(1):96-102.

48. Dako-Gyeke P, Dornoo B, Ayisi Addo S, et al. Towards elimination of mother-to-child transmission of HIV in Ghana: an analysis of national programme data. *International journal for equity in health* 2016;15:5. doi: 10.1186/s12939-016-0300-5 [published Online First: 2016/01/14]

49. Delvaux T, Samreth S, Barr-DiChiara M, et al. Linked response for prevention, care, and treatment of HIV/AIDS, STIs, and reproductive health issues: results after 18 months of implementation in five operational districts in Cambodia. *Journal of acquired immune deficiency syndromes (1999)* 2011;57(3):e47-55. doi: 10.1097/QAI.0b013e318216d5cf [published Online First: 2011/03/17]

50. Draper B, Abdullah F. A review of the prevention of mother-to-child transmission programme of the Western Cape provincial government, 2003 - 2004. *South African Medical Journal* 2008;98(6):431-4.

51. Global AIDS Response. Global AIDS Response Progress Reporting 2016. Geneva: UNAIDS, 2016.

52. Herce ME, Mtande T, Chimbwandira F, et al. Supporting Option B+ scale up and strengthening the prevention of mother-to-child transmission cascade in central Malawi: results from a serial cross-

For peer review only

sectional study. *BMC infectious diseases* 2015;15:328. doi: 10.1186/s12879-015-1065-y [published Online First: 2015/08/13]

53. Mukherjee S, Ghosh S, Goswami DN, et al. Performance evaluation of PPTCT (Prevention of parent to child transmission of HIV) programme: an experience from West Bengal. *The Indian journal of medical research* 2012;136(6):1011-9. [published Online First: 2013/02/09]

54. Baker U, Okuga M, Waiswa P, et al. Bottlenecks in the implementation of essential screening tests in antenatal care: Syphilis, HIV, and anemia testing in rural Tanzania and Uganda. *International journal of gynaecology and obstetrics: the official organ of the International Federation of Gynaecology and Obstetrics* 2015;130 Suppl 1:S43-50. doi: 10.1016/j.ijgo.2015.04.017 [published Online First: 2015/06/10]

55. Hossain M, Broutet N, Hawkes S. The elimination of congenital syphilis: A comparison of the proposed world health organization action plan for the elimination of congenital syphilis with existing national maternal and congenital syphilis policies. *Sexually Transmitted Diseases* 2007;34(7):S22-S30. doi: 10.1097/olq.0000261049.84824.40

56. Teferi E, Alemu H, Bulto T, et al. A descriptive study of the changes in coverage of preventive and promotive interventions before and after the introduction of integrated community case management (ICCM) in Ethiopia. *Ethiopian medical journal* 2014;52 Suppl 3:151-5. [published Online First: 2015/04/08]

57. MEASURE Evaluation, MEASURE DHS, President's Malaria Initiative, et al. Household Survey Indicators for Malaria Control. Chapel Hill, North Carolina, USA: MEASURE Evaluation, 2013.

58. WHO. Malaria in pregnancy: Guidelines for measuring key monitoring and evaluation indicators. Geneva: WHO, 2007:[38] p.

59. WHO. Consolidated Strategic Information Guidelines for HIV in the Health Sector. Geneva: World Health Organization, 2015a.

For peer review only

60. McPherson RA, Khadka N, Moore JM, et al. Are birth-preparedness programmes effective? Results from a field trial in Siraha district, Nepal. *Journal of health, population, and nutrition* 2006;24(4):479-88. [published Online First: 2007/06/27]

61. Bookallil M, Chalmers E, Andrew B. Challenges in preventing pyelonephritis in pregnant women in Indigenous communities. *Rural and remote health* 2005;5(3):395. [published Online First: 2005/08/09]

62. Olagbuji BN, Igbarumah S, Akintayo AA, et al. Maternal understanding of fetal movement in third trimester: a means for fetal monitoring and reducing stillbirth. *Nigerian journal of clinical practice* 2014;17(4):489-94. doi: 10.4103/1119-3077.134049 [published Online First: 2014/06/10]

63. Peat AM, Stacey T, Cronin R, et al. Maternal knowledge of fetal movements in late pregnancy. *The Australian & New Zealand journal of obstetrics & gynaecology* 2012;52(5):445-9. doi: 10.1111/j.1479-828X.2012.01462.x [published Online First: 2012/07/04]

64. MICS. Questionnaire for Individual Women. MICS6 Questionnaires. New York: UNICEF, 2017.

65. Bohren MA, Titiloye MA, Kyaddondo D, et al. Defining quality of care during childbirth from the perspectives of Nigerian and Ugandan women: A qualitative study. *International journal of gynaecology and obstetrics: the official organ of the International Federation of Gynaecology and Obstetrics* 2017;139 Suppl 1:4-16. doi: 10.1002/ijgo.12378

66. Shakibazadeh E, Namadian M, Bohren MA, et al. Respectful care during childbirth in health facilities globally: a qualitative evidence synthesis. *BJOG* 2017 doi: 10.1111/1471-0528.15015

67. Downe S, Finlayson K, Tuncalp, et al. What matters to women: a systematic scoping review to identify the processes and outcomes of antenatal care provision that are important to healthy pregnant women. *BJOG* 2016;123(4):529-39. doi: 10.1111/1471-0528.13819

For peer review only

68. Benova L, Tunçalp Ö, Moran AC, et al. Not just a number: examining coverage and content of antenatal care in low-income and middle-income countries. *BMJ Global Health* 2018;3(2) doi: 10.1136/bmjgh-2018-000779

69. Jolivet RR, Moran AC, O'Connor M, et al. Ending preventable maternal mortality: phase II of a multi-step process to develop a monitoring framework, 2016-2030. *BMC Pregnancy and Childbirth* 2018;18(1):258. doi: 10.1186/s12884-018-1763-8

70. Balakrishnan R, Gopichandran V, Chaturvedi S, et al. Continuum of Care Services for Maternal and Child Health using mobile technology - a health system strengthening strategy in low and middle income countries. *BMC Medical Informatics and Decision Making* 2016;16(84):1-8. doi: http://dx.doi.org/10.1186/s12911-016-0326-z

71. WHO. Reproductive health indicators: guidelines for their generation, interpretation and analysis for global monitoring. Geneva: WHO, 2006:63 p.

72. Say L, Barreix M, Chou D, et al. Maternal morbidity measurement tool pilot: study protocol. *Reproductive health* 2016;13(1):69. doi: 10.1186/s12978-016-0164-6 [published Online First: 2016/06/10]

73. Chou D, Tuncalp O, Firoz T, et al. Constructing maternal morbidity - towards a standard tool to measure and monitor maternal health beyond mortality. *BMC pregnancy and childbirth* 2016;16:45. doi: 10.1186/s12884-015-0789-4

74. Andreucci CB, Cecatti JG, Macchetti CE, et al. SISPRENATAL as a tool for evaluating quality of prenatal care. *Revista de saude publica* 2011;45(5):854-64. [published Online First: 2011/08/24]

## Figure legends

For peer review only

**Figure 1: WHO framework for the quality of antenatal care.** DHS = Demographic and Health Surveys. MICS = Multiple Indicator Cluster Surveys. WHO = World Health Organization. WoS = Web of Science.

**Figure 2: An overview of the scoping review's search and screening processes.**

For peer review only

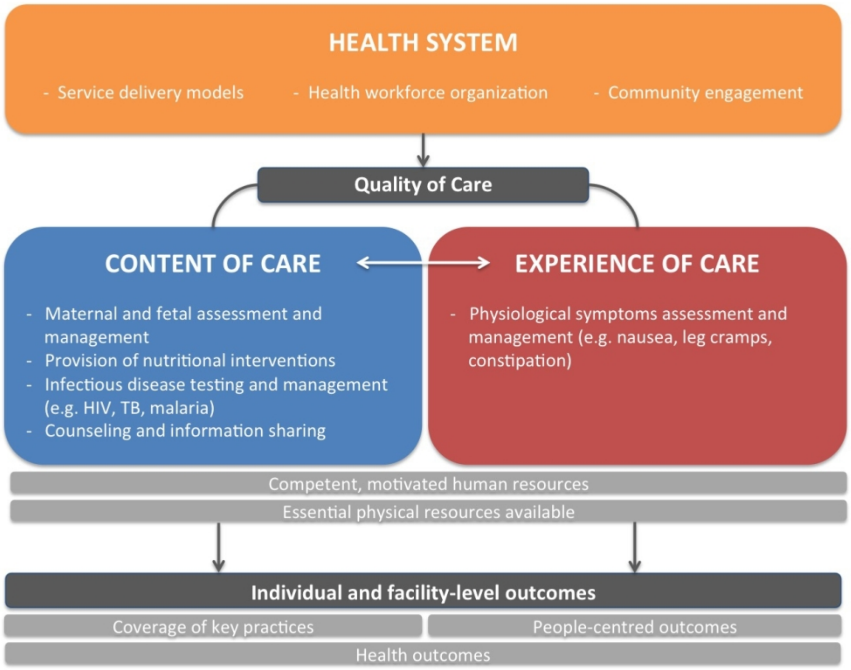


Figure 1: WHO framework for the quality of antenatal care. DHS = Demographic and Health Surveys. MICS = Multiple Indicator Cluster Surveys. WHO = World Health Organization. WoS = Web of Science.

528x396mm (72 x 72 DPI)

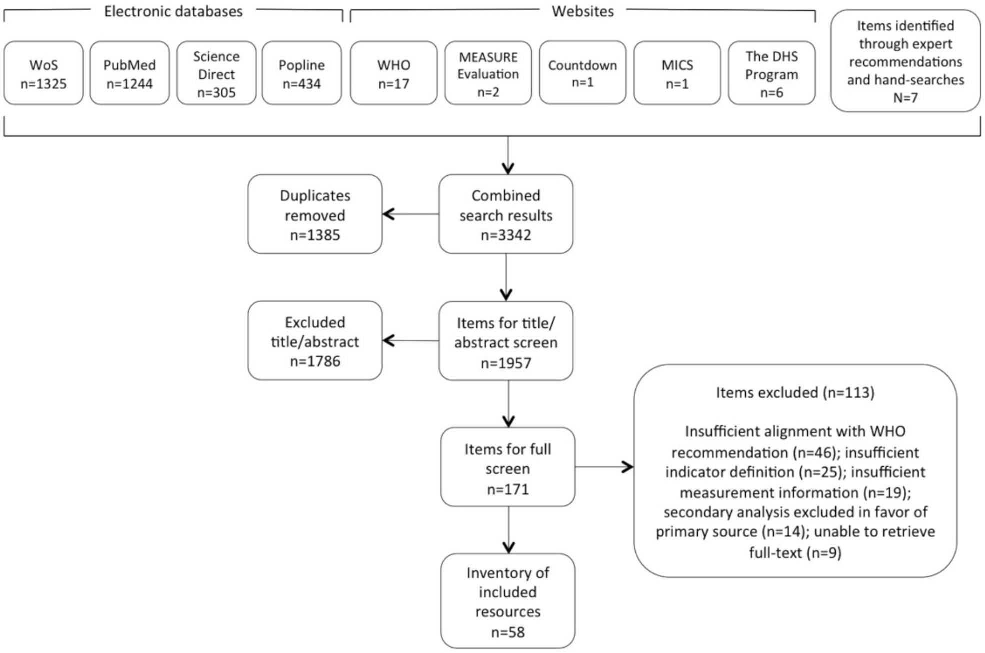


Figure 2: An overview of the scoping review's search and screening processes

528x396mm (300 x 300 DPI)

**Table A1: Current measures for ANC recommendations**

| Domain | WHO ANC Recommendation | Measure | Definition(s) | Numerator(s) | Denominator(s) |
|---|---|---|---|---|---|
| Content of Care | A.2.1 - Daily oral iron and folic acid supplementation with 30 mg to 60 mg of elemental iron and 400 g (0.4 mg) of folic acid is recommended for pregnant women to prevent maternal anaemia, puerperal sepsis, low birth weight, and preterm birth | Iron and folic acid supplements for pregnant women (%) | Percentage of pregnant women who received iron-folic acid supplementation for 90+ days[1 2]<br><br>Percent of women with a birth in the last two years who received *or bought* iron/folic acid supplements *for at least six months* during their last pregnancy in amounts that were in accordance with recommended protocols[3]<br><br>Proportion of pregnant women given iron and folic acid supplements[4]<br><br>Percentage of respondents who received any iron/folate during pregnancy[5] | Number of pregnant women who received the recommended number of iron/folic acid tablets during last pregnancy[1]<br><br>Number of pregnant women given 90+ IFA tablets[2]<br><br>Number of pregnant women who received *or purchased* the recommended number of iron/folic acid tablets during last pregnancy[3]<br><br>Number of pregnant women given iron and folic acid supplements[4]<br><br>Number of respondents who received any iron/folate during pregnancy[5] | Total number of pregnant women with a birth in last two years[1 3]<br><br>Number of pregnant women in health registers or monthly summary forms[2]<br><br>All eligible pregnancies (a woman having one documented ANC visit (unless the ANC visit is only for termination of pregnancy), OR any data documenting a pregnancy outcome or infant at any point of care)[4]<br><br>Total number of women surveyed who had delivered a live or stillbirth child during the year before the interview date[5] |
| Content of Care | A.2.1 - Daily oral iron and folic acid supplementation with 30 mg to 60 mg of elemental iron and 400 g (0.4 mg) of folic acid is recommended for pregnant women to prevent maternal anaemia, puerperal sepsis, low birth weight, and preterm birth | Preventive treatment with folic acid | Preventive treatment with folic acid: ratio of number of patients that completed folic acid prophylaxis to total number of pregnant women during the year[6] | Number of patients that completed folic acid prophylaxis | Total number of pregnant women during the year |
| Content of Care | A.2.1 - Daily oral iron and folic acid supplementation with 30 mg to 60 mg of elemental iron and 400 g (0.4 mg) of folic acid is recommended for pregnant women to prevent maternal anaemia, puerperal sepsis, low birth weight, and preterm birth | IFA supplementation | Mother consumed at least 100 iron-folic acid tablets during pregnancy[7 8] | Number of mothers who consumed at least 100 iron-folic acid tablets during pregnancy | Total number of study mothers (survey respondents who had a live birth in the reference period for each survey) |
| Content of Care | A.2.1 - Daily oral iron and folic acid supplementation with 30 mg to 60 mg of elemental iron and 400 g (0.4 mg) of folic acid is recommended for pregnant women to prevent maternal anaemia, puerperal sepsis, low birth weight, and preterm birth | ANC visits where the client received a 3-month supply of iron supplements and a 3-month supply of folate supplements | Number of ANC visits at facility per month where the client received a 3-month supply of iron supplements; Number of ANC visits at facility per month where the client received a 3-month supply of folate supplements[9] | Number of ANC visits per month where the client received a 3-month supply of iron supplements; Number of ANC visits at facility per month where the client received a 3-month supply of folate supplements | Total number of ANC visits at facility per month (= new visits + revisits) |

For peer review only

| Domain | WHO ANC Recommendation | Measure | Definition(s) | Numerator(s) | Denominator(s) |
|---|---|---|---|---|---|
| Content of Care | A.2.1 - Daily oral iron and folic acid supplementation with 30 mg to 60 mg of elemental iron and 400 g (0.4 mg) of folic acid is recommended for pregnant women to prevent maternal anaemia, puerperal sepsis, low birth weight, and preterm birth | Percentage of health facilities routinely providing iron and folic acid | Percentage of health facilities routinely providing iron and folic acid during ANC[10] | Number of health facilities where iron and folic acid supplementation is routinely provided during ANC | Total number of facilities surveyed |
| Content of Care | A.2.1 - Daily oral iron and folic acid supplementation with 30 mg to 60 mg of elemental iron and 400 g (0.4 mg) of folic acid is recommended for pregnant women to prevent maternal anaemia, puerperal sepsis, low birth weight, and preterm birth | Folic acid and iron supplementation in pregnancy | 1. Women reporting any folic acid supplementation during their pregnancy; and 2. Women reporting any iron supplementation during their pregnancy[11] | 1. Number of survey respondents reporting any folic acid supplementation during their pregnancy; 2. Number of survey respondents reporting any iron supplementation during their pregnancy<br><br>-OR- 1. Number of antenatal records reporting any folic acid supplementation during pregnancy; 2. Number of antenatal records reporting any iron supplementation during pregnancy | Total number of survey respondents<br><br>-OR- total number of antenatal records reviewed |
| Content of Care | A.2.2 - Intermittent oral iron and folic acid supplementation with 120 mg of elemental iron and 2800 g (2.8 mg) of folic acid once weekly is recommended for pregnant women to improve maternal and neonatal outcomes if daily iron is not acceptable due to side-effects, and in populations with an anaemia prevalence among pregnant women of less than 20% | Iron and folic acid supplements for pregnant women (%) | Percentage of pregnant women who received iron-folic acid supplementation for 90+ days[1]<br><br>Percent of women with a birth in the last two years who received *or bought* iron/folic acid supplements *for at least six months* during their last pregnancy in amounts that were in accordance with recommended protocols[3] | Number of pregnant women who received the recommended number of iron/folic acid tablets during last pregnancy[1]<br><br>Number of pregnant women who received *or purchased* the recommended number of iron/folic acid tablets during last pregnancy[3] | Total number of pregnant women with a birth in last two years |
| Health Systems | B.1.3 - Clinical enquiry about the possibility of intimate partner violence (IPV) should be strongly considered at antenatal care visits when assessing conditions that may be caused or complicated by IPV in order to improve clinical diagnosis and subsequent care, where there is the capacity to provide a supportive response (including referral where appropriate) and where the WHO minimum requirements are met | Percent of health units with at least one service provider trained to care for and refer SGBV survivors | Percent of health facilities in the geographic region of study (e.g., country, region, community) with at least one provider who has been trained within the past three years in the identification, care and support of sexual and gender-based violence (SGBV) survivors[3,12] | Number of health facilities reporting that they have both documented and adopted a protocol for the clinical management of SGBV survivors | Total number of health facilities surveyed |

For peer review only

| Domain | WHO ANC Recommendation | Measure | Definition(s) | Numerator(s) | Denominator(s) |
|---|---|---|---|---|---|
| Health Systems | B.1.3 - Clinical enquiry about the possibility of intimate partner violence (IPV) should be strongly considered at antenatal care visits when assessing conditions that may be caused or complicated by IPV in order to improve clinical diagnosis and subsequent care, where there is the capacity to provide a supportive response (including referral where appropriate) and where the WHO minimum requirements are met | Proportion of women who were asked about physical and sexual violence during a visit to a health unit | Proportion of women who presented to the clinic for any reason who were asked about physical or sexual violence, during a specific period of time (e.g., during the past 12 months)[12 13] | Number of women who were asked, during the course of their service provision at the unit, about any violence that had ever occurred, either physical or sexual, in the geographic area of study (nation, province, state, community).<br><br>If measured with a medical record review, all women's charts that note they were asked if they experienced any physical and sexual violence by a provider would be entered into the numerator.<br><br>If measured in a survey of women based on exit interviews from the health unit, all women leaving the clinic would be asked if a provider asked them if they had ever experienced any physical or sexual violence. All women answering yes would be entered into the numerator. | If the indicator is measured through a record review, this is the number of women's records that were reviewed at the health unit.<br><br>If the indicator is being measured through an exit interview, this is the total number of women interviewed. |
| Content of Care | B.1.5 - Health-care providers should ask all pregnant women about their tobacco use (past and present) and exposure to second-hand smoke as early as possible in the pregnancy and at every antenatal care visit | Proportion of pregnant women whose smoking status was checked at the first antenatal visit | Proportion of pregnant women whose smoking status was checked at the first antenatal visit[4] | Number of pregnant women whose smoking status was checked at the first ANC visit | Total number of eligible pregnancies (a woman having one documented ANC visit (unless the ANC visit is only for termination of pregnancy), OR any data documenting a pregnancy outcome or infant at any point of care) |
| Content of Care | B.1.5 - Health-care providers should ask all pregnant women about their tobacco use (past and present) and exposure to second-hand smoke as early as possible in the pregnancy and at every antenatal care visit | Cigarette smoking screened for in pregnancy | Percentage of women screened for smoking in pregnancy[14] | Number of medical records that indicate screening for smoking in pregnancy occurred | Total number of medical records reviewed |

For peer review only

| Domain | WHO ANC Recommendation | Measure | Definition(s) | Numerator(s) | Denominator(s) |
|---|---|---|---|---|---|
| Content of Care | B.1.7 - In high-prevalence settings, provider-initiated testing and counselling (PITC) for HIV should be considered a routine component of the package of care for pregnant women in all antenatal care settings. In low-prevalence settings, PITC can be considered for pregnant women in antenatal care settings as a key component of the effort to eliminate mother-to-child transmission of HIV, and to integrate HIV testing with syphilis, viral or other key tests, as relevant to the setting, and to strengthen the underlying maternal and child health systems | Pregnant women counselled and tested for HIV and know their results (%) | Percentage of pregnant women attending antenatal care (ANC), labour & delivery (L&D), and postpartum care services (PPC), plus women with known HIV infection attending ANC for a new pregnancy, who received testing and counselling for HIV in the last 12 months and who know their HIV test results[3] | The numerator can be summed from the categories below:<br>- Number of pregnant women who received an HIV test and result during ANC<br>- Number of pregnant women attending labour and delivery (L&D) with unknown HIV status who were tested in the L&D and received results<br>- Women with unknown HIV status attending postpartum services within 72 hours of delivery who were tested and received results<br>- Pregnant women with known HIV infection attending ANC for a new pregnancy. | The denominator is generated through a population estimate of the number of pregnant women giving birth in the last 12 months, which can be obtained from the Central Statistics Office estimates of births or the UN Population Division estimates. In countries with low-level and concentrated epidemics where policies to identify the HIV status of all pregnant women do not exist, the denominator should be adapted to the target population of pregnant women whose HIV status should be assessed. |
| Content of Care | B.1.7 - In high-prevalence settings, provider-initiated testing and counselling (PITC) for HIV should be considered a routine component of the package of care for pregnant women in all antenatal care settings. In low-prevalence settings, PITC can be considered for pregnant women in antenatal care settings as a key component of the effort to eliminate mother-to-child transmission of HIV, and to integrate HIV testing with syphilis, viral or other key tests, as relevant to the setting, and to strengthen the underlying maternal and child health systems | Number of pregnant women tested for HIV | Number of pregnant women attending ANC who are tested for HIV[15]<br><br>Number of ANC registrants receiving HIV testing and counselling[16]<br><br>Proportion of antenatal patients tested[17]<br><br>Proportion of pregnant women undergoing HIV testing and counselling during ANC[18]<br><br>Number (%) of newly registered antenatal cases tested for HIV[19]<br><br>Proportion of pregnant women who received testing and counselling at the first antenatal visit and received their results[4]<br><br>Proportion of pregnant women tested for HIV (of ANC1 and of total expected pregnant women)[20]<br><br>Proportion of pregnant women tested for HIV[2] | Total number of pregnant women attending ANC who are tested[15]<br><br>Number of ANC registrants receiving HIV testing and counselling[16]<br><br>Number of pregnant women with a new HTC test result documented in ANC clinic[18]<br><br>Number of newly registered antenatal cases tested for HIV[19]<br><br>Number of pregnant women who received testing and counselling at the first antenatal visit and received their results[4]<br><br>Number of pregnant women tested for HIV[2 20] | Total # of pregnant women attending ANC[15]<br><br>Total number of ANC registrants[16]<br><br>Total number of pregnant women presenting to ANC clinic without a prior documented positive HIV test result[18]<br><br>All new antenatal cases registered for the first time at any of the Integrated Counselling and Testing Centres that provided PPTCT services during any time of the antenatal period and also those cases who were registered for the first time at the time of admission during labour[19]<br><br>Total number of eligible pregnancies (a woman having one documented ANC visit (unless the ANC visit is only for termination of pregnancy), OR any data documenting a pregnancy outcome or infant at any point of care)[4]<br><br>Number of expected pregnant women[20]<br><br>Number of pregnant women in health registers or monthly summary forms[2] |

For peer review only

| Domain | WHO ANC Recommendation | Measure | Definition(s) | Numerator(s) | Denominator(s) |
|---|---|---|---|---|---|
| Content of Care | B.1.7 - In high-prevalence settings, provider-initiated testing and counselling (PITC) for HIV should be considered a routine component of the package of care for pregnant women in all antenatal care settings. In low-prevalence settings, PITC can be considered for pregnant women in antenatal care settings as a key component of the effort to eliminate mother-to-child transmission of HIV, and to integrate HIV testing with syphilis, viral or other key tests, as relevant to the setting, and to strengthen the underlying maternal and child health systems | Mothers of children 0-23 months who received HIV testing and counselling services during pregnancy (%) | % of mother of children 0-23 months who received HIV testing and counselling services during pregnancy[21] | # of mothers counselled and tested for HIV | Total # of mothers interviewed |
| Content of Care | B.1.7 - In high-prevalence settings, provider-initiated testing and counselling (PITC) for HIV should be considered a routine component of the package of care for pregnant women in all antenatal care settings. In low-prevalence settings, PITC can be considered for pregnant women in antenatal care settings as a key component of the effort to eliminate mother-to-child transmission of HIV, and to integrate HIV testing with syphilis, viral or other key tests, as relevant to the setting, and to strengthen the underlying maternal and child health systems | Percentage of health facilities routinely providing voluntary counselling and testing for HIV/AIDS | Percentage of health facilities routinely providing ANC clients with voluntary counselling and testing for HIV/AIDS[10] | Number of health facilities routinely providing ANC clients with voluntary counselling and testing for HIV/AIDS | Total number of facilities surveyed |
| Content of Care | B.1.7 - In high-prevalence settings, provider-initiated testing and counselling (PITC) for HIV should be considered a routine component of the package of care for pregnant women in all antenatal care settings. In low-prevalence settings, PITC can be considered for pregnant women in antenatal care settings as a key component of the effort to eliminate mother-to-child transmission of HIV, and to integrate HIV testing with syphilis, viral or other key tests, as relevant to the setting, and to strengthen the underlying maternal and child health systems | HIV testing in pregnant women | Percentage of pregnant women with known HIV status[22] | Number of pregnant women attending antenatal clinics (ANC) and/or had a facility-based delivery and were tested for HIV during pregnancy, or already knew they were HIV positive | Population-based denominator: Number of pregnant women who delivered within the past 12 months<br><br>Programme-based denominator: Number of pregnant women who attended an ANC or had a facility-based delivery in the past 12 months |

For peer review only

| Domain | WHO ANC Recommendation | Measure | Definition(s) | Numerator(s) | Denominator(s) |
|---|---|---|---|---|---|
| Content of Care | B.1.7 - In high-prevalence settings, provider-initiated testing and counselling (PITC) for HIV should be considered a routine component of the package of care for pregnant women in all antenatal care settings. In low-prevalence settings, PITC can be considered for pregnant women in antenatal care settings as a key component of the effort to eliminate mother-to-child transmission of HIV, and to integrate HIV testing with syphilis, viral or other key tests, as relevant to the setting, and to strengthen the underlying maternal and child health systems | Antenatal care: tested for syphilis (%) | Percent of women attending antenatal care services who were tested for syphilis[3]<br><br>Proportion of pregnant women tested for syphilis[2 20]<br><br>Proportion attending ANC at least once in a health facility where syphilis tests are available who report having received a test result for syphilis[23] | Number of women in antenatal care (ANC) who were screened for syphilis during pregnancy[3 24]<br><br>Number of women attending ANC services within the past 12 months who were tested for syphilis[25]<br><br>Number of pregnant women who were screened for syphilis at the first antenatal visit[4]<br><br>Number of pregnant women tested for syphilis[2 20]<br><br>Number of women with a live birth in the 12 months prior to the household survey who attended ANC at least once in a health facility where syphilis tests are available who report having received a test result for syphilis[23] | Number of pregnant women attending ANC clinics[3 24]<br><br>Number of women attending ANC services within the past 12 months[25]<br><br>Total number of eligible pregnancies (a woman having one documented ANC visit (unless the ANC visit is only for termination of pregnancy), OR any data documenting a pregnancy outcome or infant at any point of care)[4]<br><br>Number of expected pregnant women[20]<br><br>Number of pregnant women in health registers or monthly summary forms[2]<br><br>Number of women with a live birth in the 12 months prior to the household survey[23] |
| Content of Care | B.1.7 - In high-prevalence settings, provider-initiated testing and counselling (PITC) for HIV should be considered a routine component of the package of care for pregnant women in all antenatal care settings. In low-prevalence settings, PITC can be considered for pregnant women in antenatal care settings as a key component of the effort to eliminate mother-to-child transmission of HIV, and to integrate HIV testing with syphilis, viral or other key tests, as relevant to the setting, and to strengthen the underlying maternal and child health systems | Measures to ensure all pregnant women are screened and treated for syphilis | Existence of formal policy documents and national treatment protocols addressing maternal syphilis screening and treatment[26] | Number of countries with national-level policies addressing maternal syphilis screening and treatment | Total number of countries whose policies were reviewed |

For peer review only

| Domain | WHO ANC Recommendation | Measure | Definition(s) | Numerator(s) | Denominator(s) |
|---|---|---|---|---|---|
| Content of Care | B.2.1 - Daily fetal movement counting, such as with “count-to-ten” kick charts, is only recommended in the context of rigorous research | Antenatal care: fetal movement enquiry (%) | Number of pregnant women >28 weeks attending ANC who reported that antenatal care providers routinely enquired about fetal movement at each prenatal visit[27]<br><br>Proportion of survey respondents (gestational age >28 weeks) who reported that their lead maternity carer (LMC) regularly enquired about their baby’s movements[28] | Number of pregnant women >28 weeks attending ANC who reported that antenatal care providers routinely enquired about fetal movement at each prenatal visit[27]<br><br>Number of pregnant women attending ANC (gestational age >28 weeks) who reported that their lead maternity carer regularly enquired about their baby’s movements[28] | Total number of pregnant women >28 weeks attending ANC in the reporting period (who were surveyed) |
| Content of Care | C.1 - A seven-day antibiotic regimen is recommended for all pregnant women with asymptomatic bacteriuria (ASB) to prevent persistent bacteriuria, preterm birth and low birth weight | Number of episodes where immediate treatment was given per episode of positive urinalysis | Number of episodes where immediate treatment was given per episode of positive urinalysis[29] | Number of episodes of positive (abnormal) urinalysis where women received immediate treatment with antibiotics | Number of episodes of positive (abnormal) urinalysis in all charts reviewed |
| Content of Care | C.4 - In endemic areas, preventive anthelminthic treatment is recommended for pregnant women after the first trimester as part of worm infection reduction programmes | Percent of pregnant women who receive anthelminthic treatment during pregnancy | In areas of moderate to high endemicity of helminths (parasitic worms), the percent of pregnant women who receive presumptive anthelminthic treatment during their pregnancy. According to the 1998 IVACG/WHO/UNICEF "Guidelines for the Use of Iron Supplements to Prevent and Treat Iron Deficiency Anemia," treatment should be done once in the second and third trimester. In areas of low endemicity: the percent of pregnant women who received prescribed treatment during their pregnancy.[3]<br><br>Percentage of respondents who received deworming with albendazole during pregnancy[5]<br><br>Proportion of pregnant women provided deworming[2] | # of pregnant women who receive presumptive antihelminthic treatment[3]<br><br>Number of respondents who received deworming with albendazole during pregnancy[5]<br><br>Number of pregnant women provided deworming[2] | Total # of pregnant women[3]<br><br>Total number of women surveyed who had delivered a live or stillbirth child during the year before the interview date[5]<br><br>Number of pregnant women in health registers or monthly summary forms[2] |
| Content of Care | C.5 - Tetanus toxoid vaccination is recommended for all pregnant women, depending on previous tetanus vaccination exposure, to prevent neonatal mortality from tetanus | Percentage of newborns protected at birth from tetanus (takes into account mother's doses prior to pregnancy) | Percentage of newborns protected against tetanus[30] | Number of mothers with a live birth in the year prior to the survey who received two doses of tetanus toxoid vaccine within the appropriate interval prior to the infant’s birth | Total number of women ages 15–49 with a live birth in the year prior to the survey |
| Content of Care | C.5 - Tetanus toxoid vaccination is recommended for all pregnant women, depending on previous tetanus vaccination exposure, to prevent neonatal mortality from tetanus | Percentage of health facilities offering tetanus toxoid vaccination services | Percentage of health facilities where tetanus toxoid vaccination services are available each day that ANC services are provided[10] | Number of health facilities where tetanus toxoid vaccination services are available each day that ANC services are provided | Total number of facilities surveyed |

For peer review only

| Domain | WHO ANC Recommendation | Measure | Definition(s) | Numerator(s) | Denominator(s) |
|---|---|---|---|---|---|
| Content of Care | C.5 - Tetanus toxoid vaccination is recommended for all pregnant women, depending on previous tetanus vaccination exposure, to prevent neonatal mortality from tetanus | Percent of women who received at least two doses of tetanus-toxoid vaccine in their last pregnancy | Proportion of pregnant women receiving at least two doses of tetanus-toxoid vaccine (TT2+)[2 3 7 8]<br><br>Percentage of respondents who received tetanus toxoid 2 doses during pregnancy or lifetime 5 doses[5] | Total TT2 + TT3 + TT4 + TT5 x 100 (Where TT2, TT3, TT4, TT5 refer to the 2nd, 3rd, 4th, or 5th dose of tetanus-toxoid vaccine administered)<br><br>Number of pregnant women given TT2 immunization[2]<br><br>Number of respondents who received tetanus toxoid 2 doses during pregnancy or lifetime 5 doses[5]<br><br>Number of respondents who received two or more TT injections[8] | Total # of live births<br><br>Number of pregnant women in health registers or monthly summary forms[2]<br><br>Total number of women surveyed who had delivered a live or stillbirth child during the year before the interview date[5]<br><br>Total number of ever married women surveyed in the age group 15-49 years[8] |
| Content of Care | C.5 - Tetanus toxoid vaccination is recommended for all pregnant women, depending on previous tetanus vaccination exposure, to prevent neonatal mortality from tetanus | Number of third trimester ANC visits protected against tetanus (at least TT2) per month | Number of third trimester (≥28 weeks) ANC visits protected against tetanus (at least TT2) per month[9] | Number of third trimester ANC visits protected against tetanus (at least TT2) per month | Total number of third trimester (>28 weeks) ANC visits at facility per month |
| Content of Care | C.5 - Tetanus toxoid vaccination is recommended for all pregnant women, depending on previous tetanus vaccination exposure, to prevent neonatal mortality from tetanus | Rate of tetanus vaccination | Rate of tetanus vaccination: ratio of number of pregnant women with complete vaccinations (verified at delivery) to total number of pregnant women during the year[6] | Number of pregnant women with complete vaccinations (verified at delivery) | Total number of pregnant women during the year |
| Content of Care | C.5 - Tetanus toxoid vaccination is recommended for all pregnant women, depending on previous tetanus vaccination exposure, to prevent neonatal mortality from tetanus | Mothers who received at least two tetanus toxoid vaccinations before the birth of their youngest child (%) | Percentage of mothers with children age 0-23 months who received at least two tetanus toxoid vaccinations before the birth of their youngest child[21]<br><br>Mothers of children 0-11 months who received ≥2 antenatal tetanus toxoid vaccinations[31] | # of Mothers who receiving at least 2 TT injections (Q19 + Q21=>2)[21]<br><br>Mothers of children 0-11 months who received ≥2 antenatal tetanus toxoid vaccinations[31] | Total # mothers interviewed[21]<br><br>Total number of mothers of children 0-11 months surveyed[31] |

For peer review only

| Domain | WHO ANC Recommendation | Measure | Definition(s) | Numerator(s) | Denominator(s) |
|---|---|---|---|---|---|
| Content of Care | C.6 - In malaria-endemic areas in Africa, intermittent preventive treatment with sulfadoxine-pyrimethamine (IPTp-SP) is recommended for all pregnant women. Dosing should start in the second trimester, and doses should be given at least one month apart, with the objective of ensuring that at least three doses are received | Intermittent preventive therapy for malaria during pregnancy (IPTp) (%) | Percentage of women who received three or more doses of intermittent preventive treatment during antenatal care visits during their last pregnancy | Number of women receiving three or more doses of recommended treatment[24]<br><br>Number of women who received three or more doses of a recommended prophylactic antimalarial drug treatment, at least one of which was received during an ANC visit, to prevent malaria during their last pregnancy that led to a live birth within the last two years[32] | Total number of pregnant women surveyed who delivered a live birth in the last 2 years |
| Content of Care | C.6 - In malaria-endemic areas in Africa, intermittent preventive treatment with sulfadoxine-pyrimethamine (IPTp-SP) is recommended for all pregnant women. Dosing should start in the second trimester, and doses should be given at least one month apart, with the objective of ensuring that at least three doses are received | Percentage of pregnant women attending ANC who receive a first (and/or second/third) dose of IPTp under direct observation | Proportion of women attending ANC clinics who receive IPT1/IPT2/IPT3 as directly observed treatment by a health worker to maximize compliance[33] | Number of pregnant women who receive IPT1 (IPT2/IPT3) under observation | Number of first ANC visits (stays the same regardless of IPT1/IPT2/IPT3) |
| Content of Care | C.6 - In malaria-endemic areas in Africa, intermittent preventive treatment with sulfadoxine-pyrimethamine (IPTp-SP) is recommended for all pregnant women. Dosing should start in the second trimester, and doses should be given at least one month apart, with the objective of ensuring that at least three doses are received | Number of third trimester ANC visits receiving IPT2 (or more) per month | Number of third trimester ($\geq$28 weeks) ANC visits receiving IPT2 (or more) per month[9] | Number of third trimester ANC visits receiving IPT2 (or more) per month | Total number of third trimester (>28 weeks) ANC visits at facility per month |
| Content of Care | C.6 - In malaria-endemic areas in Africa, intermittent preventive treatment with sulfadoxine-pyrimethamine (IPTp-SP) is recommended for all pregnant women. Dosing should start in the second trimester, and doses should be given at least one month apart, with the objective of ensuring that at least three doses are received | Percentage of health facilities routinely providing preventative anti-malarial medication | Percentage of health facilities routinely providing preventative anti-malarial medication to ANC clients[10] | Number of health facilities routinely providing preventative anti-malarial medication to ANC clients | Total number of facilities surveyed |

For peer review only

| Domain | WHO ANC Recommendation | Measure | Definition(s) | Numerator(s) | Denominator(s) |
|---|---|---|---|---|---|
| Content of Care | C.7 - Oral pre-exposure prophylaxis (PrEP) containing tenofovir disoproxil fumarate (TDF) should be offered as an additional prevention choice for pregnant women at substantial risk of HIV infection as part of combination prevention approaches | PrEP coverage | % using PrEP in priority PrEP populations[25] | Number of members of the selected PrEP priority groups using PrEP within the last 12 months | Number of people in the selected PrEP priority groups |
| Health Systems | E.1 - It is recommended that each pregnant woman carries her own case notes during pregnancy to improve continuity, quality of care and her pregnancy experience | Percent of mothers of children 0-23 months with a maternal health card | Percent of mothers of children 0-23 months with a maternal health card (interviewer-confirmed)[21] | # of mothers with maternal health cards | Total # of mothers interviewed |
| Health Systems | E.6 - Policy-makers should consider educational, regulatory, financial, and personal and professional support interventions to recruit and retain qualified health workers in rural and remote areas | Health worker density and distribution | Number of health workers per 1000 population[24] | Number of health workers by cadre | Total population |
| Health Systems | E.7 - Antenatal care models with a minimum of eight contacts are recommended to reduce perinatal mortality and improve women's experience of care | Antenatal care (eight or more visits) (%) | Percentage of women aged 15–49 years with a live birth in a given time period who received antenatal care, eight times or more[1] | Number of women aged 15–49 years with a live birth in a given time period who received antenatal care eight or more times | Total number of women aged 15–49 years with a live birth in the same period<br><br>All female respondents in the household who have reached he age of menstruation (12 years and older) and were/are pregnant in the last 12 months[34] |
| Content of Care | Counselling about family planning | Percentage of health facilities routinely counselling ANC clients about family planning | Percentage of health facilities routinely counselling ANC clients about family planning[10] | Number of health facilities routinely counselling ANC clients about family planning | Total number of facilities surveyed |
| Content of Care | Counselling about family planning | Counselling on family planning | Percentage of pregnant women counselled on family planning[2] | Number of pregnant women counselled on family planning | Number of pregnant women in health registers or monthly summary forms |
| Content of Care | Blood pressure monitored | Blood pressure: At least six measures in routine antenatal care programme | National guidelines recommending at least six measures of blood pressure in routine antenatal care[35] | Number of national guidelines recommending at least six measures of blood pressure in routine antenatal care | Number of national guidelines reviewed |

For peer review only

| Domain | WHO ANC Recommendation | Measure | Definition(s) | Numerator(s) | Denominator(s) |
|---|---|---|---|---|---|
| Content of Care | Blood pressure monitored | Blood pressure: At least # measure(s) during pregnancy | Woman-reported content of antenatal care received at least once (or "x" times) during pregnancy: blood pressure measured | Number of women with at least one antenatal contact with any provider during pregnancy who reported that their blood pressure was measured at least once during pregnancy[36] | (1) all women with a live birth in the last 12 months prior to the survey; or (2) women who had at least one antenatal contact with any provider during that pregnancy[36] |
| | | | | Blood pressure checked at least five times during antenatal care[37] | All mothers in the sample who underwent prenatal care and delivery in the public health service whose children were born alive[37] |
| Content of Care | Blood pressure monitored | ANC visits with blood pressure reading documented | Number of ANC visits at facility per month with blood pressure reading documented[9] | Number of ANC visits per month with a documented blood pressure reading | Total number of ANC visits at facility per month (= new visits + revisits) |
| Content of Care | Blood pressure monitored | Receipt of basic ANC services | Receipt of all three basic services during antenatal care: blood pressure measurement, blood sample taken, urine sample taken[38] | Number of women surveyed who received all three basic services during antenatal care: blood pressure measurement, blood sample taken, urine sample taken | Total number of women surveyed |
| Content of Care | Blood pressure monitored | Blood pressure measured at last exam | Percentage of women who had their blood pressure measured at their most recent ANC visit | Number of survey/interview respondents reporting that their blood pressure was measured at their most recent ANC visit | Total number of pregnant women using ANC during the data collection period who consented and were selected by systematic random sampling at the health facilities[39] |
| | | | | Number of pregnant women with blood pressure recorded[2] | Total number of client exit interviews[40] |
| | | | | | Number of pregnant women in health registers or monthly summary forms[2] |

For peer review only

| Domain | WHO ANC Recommendation | Measure | Definition(s) | Numerator(s) | Denominator(s) |
|---|---|---|---|---|---|
| Health Systems | First antenatal care visit in the first trimester | Percentage of pregnant women who began ANC during the first trimester of gestation | Proportion of pregnant women who initiate ANC in the first trimester[41]<br><br>Proportion of women with estimated gestational age at first antenatal visit < 12 weeks[42]<br><br>Proportion of women who receive antenatal assessments by 13 weeks of pregnancy[43]<br><br>Antenatal care initiated in the first trimester (<14 weeks) and minimum of 4 visits[44]<br><br>Percentage of women among whom the first medical visit occurred in the first trimester of pregnancy (or timeliness)[45]<br><br>Mothers recorded in the antenatal records with ANC in first trimester (<13 weeks)[46]<br><br>Women presenting in first trimester for first antenatal visit[14] | Number of pregnant women initiating ANC in first trimester[41]<br><br>Number of women attending ANC who began ANC during the first trimester[14]<br><br>Number of women whose first antenatal care visit was <12 weeks of gestational age[47]<br><br>Number of women whose first prenatal visit was ≤12 weeks[48]<br><br>Number of women with estimated gestational age at first antenatal visit < 12 weeks[42]<br><br>Number of pregnant women seen for ANC booking by 13 weeks 0 days[43]<br><br>Antenatal care initiated in the first trimester (<14 weeks) and minimum of 4 visits[44]<br><br>Number of women aged 15-49 surveyed who reported an obstetric episode from 2004-2009 and whose first medical visit occurred in the first trimester of pregnancy[45]<br><br>Mothers recorded in the antenatal records with ANC in first trimester (<13 weeks)[46] | Total number of women attending ANC[49]<br><br>All female respondents in the household who have reached he age of menstruation (12 years and older) and were/are pregnant in the last 12 months[34]<br><br>Total number of pregnant women using ANC during the data collection period who consented and were selected by systematic random sampling at the health facilities[39]<br><br>Total number of survey respondents; also total number of respondent electronic records[47]<br><br>Total number of medical charts reviewed[14,42,46,48]<br><br>Number of pregnant women[43]<br><br>Number of women sampled[44]<br><br>Total number of women aged 15-49 surveyed who reported an obstetric episode from 2004-2009[45] |
| Health Systems | First antenatal care visit in the first trimester | Timing of first antenatal visit (%) | Percentage of pregnant women aged 15–49 years who had their first antenatal visit in the first trimester[1] | Number of pregnant women aged 15-49 who had their first antenatal visit in the first trimester | Total number of women aged 15-49 years with at least one antenatal care visit in the same period |

For peer review only

| Domain | WHO ANC Recommendation | Measure | Definition(s) | Numerator(s) | Denominator(s) |
|---|---|---|---|---|---|
| Content of Care | Counselling about birth preparedness | Receiving ANC counselling about birth preparedness | Percentage of pregnant women who received at least one counselling session on birth preparedness | Number of women who had at least one antenatal contact with any provider during that pregnancy and report they were counselled about birth preparedness at least once during pregnancy[36]<br><br>Number of pregnant women counselled on emergency preparedness[2] | (1) all women with a live birth in the last 12 months prior to the survey; or (2) women who had at least one antenatal contact with any provider during that pregnancy[36]<br><br>Number of pregnant women in health registers or monthly summary forms[2] |
| Content of Care | Counselling about birth preparedness | Exposure to birth preparedness messages | (a) formal exposure, i.e. a respondent who received a key chain and/or was counselled using a birth preparedness flip-chart; and (b) exposure to specific birth preparedness messages, i.e. a respondent who reported having heard a specific message[50] | Formal exposure: total number of respondents who received a key chain and/or were counselled using a birth preparedness flip-chart<br><br>Message exposure: Total number of respondents exposed to specific birth preparedness messages: (a) "A pregnant women should make four antenatal care visits with a trained health worker"; (b) "A newborn should be breastfed for the first time immediately after birth"; and, (c) "A mother and newborn should have their health checked by a trained health worker within days after birth" | Total number of mothers of live infants aged less than one year at the time of the survey |

*Note: Eight of the 58 resources (references 51-58) generated by the scoping review do not appear in the truncated table above. These eight resources are tools specific to the content of ANC and may contribute to the collection of multiple measures, providing important information about indicator methodology (e.g. method of measurement, measurement frequency, data sources) and current methodological work in their linked tabulation plans and manuals.[51-58]

For peer review only

**References identified by the scoping review**


1. MoNITOR. Maternal and Newborn Indicator Mapping. Geneva: World Health Organization, 2017.

2. Dwivedi V, Drake M, Rawlins B, et al. A Review of the Maternal and Newborn Health Content of National Health Management Information Systems in 13 Countries in Sub-Saharan Africa and South Asia. Washington, D.C.: Maternal and Child Health Integrated Program, 2014.

3. MEASURE Evaluation. Summary List of Indicators Chapel Hill, North Carolina, USA: MEASURE Evaluation; 2017 [Available from: https://www.measureevaluation.org/prh/rh_indicators/indicator-summary accessed 27 Aug 2017.

4. Flenady V, Wojcieszek AM, Fjeldheim I, et al. eRegistries: indicators for the WHO Essential Interventions for reproductive, maternal, newborn and child health. *Bmc Pregnancy and Childbirth* 2016;16 doi: 10.1186/s12884-016-1049-y

5. Hodgins S, McPherson R, Suvedi BK, et al. Testing a scalable community-based approach to improve maternal and neonatal health in rural Nepal. *Journal of perinatology : official journal of the California Perinatal Association* 2010;30(6):388-95. doi: 10.1038/jp.2009.181 [published Online First: 2009/11/13]

6. Marin GH, Silberman M, Uriarte A, et al. Healthcare model based on personalised attention: impact on maternal mortality and health system quality. *Quality in primary care* 2011;19(5):311-6. [published Online First: 2011/12/22]

7. Baqui AH, Rosecrans AM, Williams EK, et al. NGO facilitation of a government community-based maternal and neonatal health programme in rural India: improvements in equity. *Health Policy and Planning* 2008;23(4):234-43. doi: 10.1093/heapol/czn012

8. Kavitha N. Are Young Mothers in India Deprived of Maternal Health Care Services? A Comparative Study of Urban and Rural Areas. *Journal of Health Management* 2015;17(2):204-20. doi: http://dx.doi.org/10.1177/0972063415575809

9. Mwaniki MK, Vaid S, Chome IM, et al. Improving service uptake and quality of care of integrated maternal health services: the Kenya Kwale District improvement collaborative. *BMC health services research* 2014;14:416. doi: 10.1186/1472-6963-14-416 [published Online First: 2014/09/23]

10. Keating J. Nigeria Baseline Health Facility Survey, 2005: [Chapel Hill, North Carolina], University of North Carolina at Chapel Hill, Carolina Population Center [CPC], MEASURE Evaluation, 2006 Oct., 2006:[78] p.

11. Ogundipe O, Hoyo C, Ostbye T, et al. Factors associated with prenatal folic acid and iron supplementation among 21,889 pregnant women in Northern Tanzania: a cross-sectional hospital-based study. *BMC public health* 2012;12:481. doi: 10.1186/1471-2458-12-481 [published Online First: 2012/06/28]

12. Bloom S. Violence against Women and Girls: A Compendium of Monitoring and Evaluation Indicators. Chapel Hill, North Carolina, USA: MEASURE Evaluation, 2008.

13. Bunn MY, Higa NA, Parker WJ, et al. Domestic violence screening in pregnancy. *Hawaii medical journal* 2009;68(10):240-2. [published Online First: 2009/12/17]


For peer review only

14. Reeve C, Banfield S, Thomas A, et al. Community outreach midwifery-led model improves antenatal access in a disadvantaged population. *The Australian journal of rural health* 2016;24(3):200-6. doi: 10.1111/ajr.12249 [published Online First: 2015/09/24]

15. Chabikuli ON, Gwarzo U, Olufunso A, et al. Closing the prevention of mother-to-child transmission gap in Nigeria: an evaluation of service improvement intervention in Nigeria. *South African Family Practice* 2013;55(1):96-102.

16. Dako-Gyeke P, Dornoo B, Ayisi Addo S, et al. Towards elimination of mother-to-child transmission of HIV in Ghana: an analysis of national programme data. *International journal for equity in health* 2016;15:5. doi: 10.1186/s12939-016-0300-5 [published Online First: 2016/01/14]

17. Draper B, Abdullah F. A review of the prevention of mother-to-child transmission programme of the Western Cape provincial government, 2003 - 2004. *South African Medical Journal* 2008;98(6):431-4.

18. Herce ME, Mtande T, Chimbwandira F, et al. Supporting Option B+ scale up and strengthening the prevention of mother-to-child transmission cascade in central Malawi: results from a serial cross-sectional study. *BMC infectious diseases* 2015;15:328. doi: 10.1186/s12879-015-1065-y [published Online First: 2015/08/13]

19. Mukherjee S, Ghosh S, Goswami DN, et al. Performance evaluation of PPTCT (Prevention of parent to child transmission of HIV) programme: an experience from West Bengal. *The Indian journal of medical research* 2012;136(6):1011-9. [published Online First: 2013/02/09]

20. Delvaux T, Samreth S, Barr-DiChiara M, et al. Linked response for prevention, care, and treatment of HIV/AIDS, STIs, and reproductive health issues: results after 18 months of implementation in five operational districts in Cambodia. *Journal of acquired immune deficiency syndromes (1999)* 2011;57(3):e47-55. doi: 10.1097/QAI.0b013e318216d5cf [published Online First: 2011/03/17]

21. Bali K, Wheeler R, Davoodnia R, et al. Partnership for Maternal and Neonatal Health - West Pokot District Child Survival and Health Program. New York: Doctors of the World, 2007.

22. Global AIDS Response. Global AIDS Response Progress Reporting 2016. Geneva: UNAIDS, 2016.

23. Baker U, Okuga M, Waiswa P, et al. Bottlenecks in the implementation of essential screening tests in antenatal care: Syphilis, HIV, and anemia testing in rural Tanzania and Uganda. *International journal of gynaecology and obstetrics: the official organ of the International Federation of Gynaecology and Obstetrics* 2015;130 Suppl 1:S43-50. doi: 10.1016/j.ijgo.2015.04.017 [published Online First: 2015/06/10]

24. WHO. Global Reference List of 100 Core Health Indicators. Geneva: World Health Organization, 2015.

25. WHO. Consolidated Strategic Information Guidelines for HIV in the Health Sector. Geneva: World Health Organization, 2015a.

26. Hossain M, Broutet N, Hawkes S. The elimination of congenital syphilis: A comparison of the proposed world health organization action plan for the elimination of congenital syphilis with existing national maternal and congenital syphilis policies. *Sexually Transmitted Diseases* 2007;34(7):S22-S30. doi: 10.1097/olq.0000261049.84824.40

For peer review only

27. Olagbuji BN, Igbarumah S, Akintayo AA, et al. Maternal understanding of fetal movement in third trimester: a means for fetal monitoring and reducing stillbirth. *Nigerian journal of clinical practice* 2014;17(4):489-94. doi: 10.4103/1119-3077.134049 [published Online First: 2014/06/10]

28. Peat AM, Stacey T, Cronin R, et al. Maternal knowledge of fetal movements in late pregnancy. *The Australian & New Zealand journal of obstetrics & gynaecology* 2012;52(5):445-9. doi: 10.1111/j.1479-828X.2012.01462.x [published Online First: 2012/07/04]

29. Bookallil M, Chalmers E, Andrew B. Challenges in preventing pyelonephritis in pregnant women in Indigenous communities. *Rural and remote health* 2005;5(3):395. [published Online First: 2005/08/09]

30. Countdown to 2015. Building a Future for Women and Children: The 2012 Report. Washington, D.C.: World Health Organization and UNICEF, 2012.

31. Teferi E, Alemu H, Bulto T, et al. A descriptive study of the changes in coverage of preventive and promotive interventions before and after the introduction of integrated community case management (ICCM) in Ethiopia. *Ethiopian medical journal* 2014;52 Suppl 3:151-5. [published Online First: 2015/04/08]

32. MEASURE Evaluation, MEASURE DHS, President's Malaria Initiative, et al. Household Survey Indicators for Malaria Control. Chapel Hill, North Carolina, USA: MEASURE Evaluation, 2013.

33. WHO. Malaria in pregnancy: Guidelines for measuring key monitoring and evaluation indicators. Geneva: WHO, 2007:[38] p.

34. Economic Growth Center (EGC) at Yale University, Institute of Statistical SERIaUoG, . Ghana Socio-economic Panel Survey: Household Instrument Wave One (2009) Part B. Living Standards Measurement Study Questionnaire. Legon, Ghana: University of Ghana, 2009.

35. Beeckman K, Frith L, Gottfreosdottir H, et al. Measuring antenatal care use in Europe: is the content and timing of care in pregnancy tool applicable? *International Journal of Public Health* 2017;62(5):583-90. doi: 10.1007/s00038-017-0959-4

36. Marchant T, Tilley-Gyado RD, Tessema T, et al. Adding Content to Contacts: Measurement of High Quality Contacts for Maternal and Newborn Health in Ethiopia, North East Nigeria, and Uttar Pradesh, India. *PloS one* 2015;10(5) doi: 10.1371/journal.pone.0126840

37. Melo EC, de Oliveira RR, Mathias TAD. Factors associated with the quality of prenatal care: an approach to premature birth. *Revista Da Escola De Enfermagem Da Usp* 2015;49(4):540-48. doi: 10.1590/s0080-623420150000400002

38. Wilunda C, Tanaka S, Putoto G, et al. Evaluation of a maternal health care project in South West Shoa Zone, Ethiopia: before-and-after comparison. *Reproductive health* 2016;13(1):95. doi: 10.1186/s12978-016-0213-1 [published Online First: 2016/08/21]

39. Ejigu T, Woldie M, Kifle Y. Quality of antenatal care services at public health facilities of Bahir-Dar special zone, Northwest Ethiopia. *BMC health services research* 2013;13:443. doi: 10.1186/1472-6963-13-443 [published Online First: 2013/10/29]

For peer review only

40. McNabb M, Chukwu E, Ojo O, et al. Assessment of the quality of antenatal care services provided by health workers using a mobile phone decision support application in northern Nigeria: a pre/post-intervention study. *PloS one* 2015;10(5):e0123940. doi: 10.1371/journal.pone.0123940 [published Online First: 2015/05/06]

41. Mbuagbaw L, Medley N, Darzi AJ, et al. Health system and community level interventions for improving antenatal care coverage and health outcomes. *The Cochrane database of systematic reviews* 2015(12):Cd010994. doi: 10.1002/14651858.CD010994.pub2 [published Online First: 2015/12/02]

42. Rumbold AR, Bailie RS, Si D, et al. Delivery of maternal health care in Indigenous primary care services: baseline data for an ongoing quality improvement initiative. *BMC pregnancy and childbirth* 2011;11:16. doi: 10.1186/1471-2393-11-16 [published Online First: 2011/03/10]

43. National Institute for Health and Care Excellence. NICE menu indicator guidance: Indicator CCG30. London: NICE, 2016.

44. Yucel C, Taskin L, Low LK. Validity and reliability of the Turkish version of the Optimality Index-US (OI-US) to assess maternity care outcomes. *Midwifery* 2015;31(12):1135-42. doi: 10.1016/j.midw.2015.08.003 [published Online First: 2015/08/25]

45. Servan-Mori E, Contreras-Loya D, Gomez-Dantes O, et al. Use of performance metrics for the measurement of universal coverage for maternal care in Mexico. *Health Policy and Planning* 2017;32(5):625-33. doi: 10.1093/heapol/czw161

46. Kildea S, Gao Y, Rolfe M, et al. Remote links: Redesigning maternity care for Aboriginal women from remote communities in Northern Australia - A comparative cohort study. *Midwifery* 2016;34:47-57. doi: 10.1016/j.midw.2016.01.009 [published Online First: 2016/03/15]

47. Liu L, Li M, Yang L, et al. Measuring coverage in MNCH: a validation study linking population survey derived coverage to maternal, newborn, and child health care records in rural China. *PloS one* 2013;8(5):e60762. doi: 10.1371/journal.pone.0060762 [published Online First: 2013/05/15]

48. Lu J, Shen JJ, Chen G, et al. Regional disparities in prenatal care services in rural China. *Asia-Pacific journal of public health* 2011;23(5):682-9. doi: 10.1177/1010539511418356 [published Online First: 2011/08/20]

49. Doubova SV, Perez-Cuevas R, Ortiz-Panozo E, et al. Evaluation of the quality of antenatal care using electronic health record information in family medicine clinics of Mexico City. *BMC pregnancy and childbirth* 2014;14:168. doi: 10.1186/1471-2393-14-168 [published Online First: 2014/06/03]

50. McPherson RA, Khadka N, Moore JM, et al. Are birth-preparedness programmes effective? Results from a field trial in Siraha district, Nepal. *Journal of health, population, and nutrition* 2006;24(4):479-88. [published Online First: 2007/06/27]

51. MICS. Questionnaire for Individual Women. MICS6 Questionnaires. New York: UNICEF, 2017.

52. The DHS Program. Measure DHS service provision assessment survey: Inventory questionnaire. SPA Questionnaires. Rockville, Maryland, USA: ICF International, 2012.

For peer review only

53. The DHS Program. Measure DHS service provision assessment survey: Observation of ANC consultation. SPA Questionnaires. Rockville, Maryland, USA: ICF International, 2012.

54. The DHS Program. Measure DHS service provision assessment survey: Health worker interview. SPA Questionnaires. Rockville, Maryland, USA: ICF International, 2012.

55. The DHS Program. Measuring service availability and readiness: A health facility assessment methodology for monitoring health system strengthening. Service Readiness Indicators. Geneva: WHO and USAID, 2012.

56. The DHS Program. Malaria Indicator Survey Model Woman's Questionnaire. Malaria Surveys. Rockville, Maryland, USA: The DHS Program, 2016.

57. The DHS Program. Measure DHS service provision assessment survey: ANC client exit interview. SPA Questionnaires. Rockville, Maryland, USA: ICF International, 2012.

58. The DHS Program. Model Woman's Questionnaire. DHS Questionnaires and Manuals - Phase 7. Rockville, Maryland, USA: ICF International, 2017.

For peer review only

**Table A2: ANC recommendations and established good clinical practices without existing measures**

| Domain | WHO ANC Recommendation or Established Good Clinical Practice |
|---|---|
| Content of Care | A.1.1 - Counselling about healthy eating and keeping physically active during pregnancy is recommended for pregnant women to stay healthy and to prevent excessive weight gain during pregnancy |
| Content of Care | A.1.2 - In undernourished populations, nutrition education on increasing daily energy and protein intake is recommended for pregnant women to reduce the risk of low-birth-weight neonates |
| Content of Care | A.1.3 - In undernourished populations, balanced energy and protein dietary supplementation is recommended for pregnant women to reduce the risk of stillbirths and small-for-gestational-age neonates |
| Content of Care | A.3 - In populations with low dietary calcium intake, daily calcium supplementation (1.5–2.0 g oral elemental calcium) is recommended for pregnant women to reduce the risk of pre-eclampsia |
| Content of Care | A.4 - Vitamin A supplementation is only recommended for pregnant women in areas where vitamin A deficiency is a severe public health problem to prevent night blindness |
| Content of Care | A.5 - Zinc supplementation for pregnant women is only recommended in the context of rigorous research |
| Content of Care | A.10 - For pregnant women with high daily caffeine intake (more than 300 mg per day), lowering daily caffeine intake during pregnancy is recommended to reduce the risk of pregnancy loss and low-birth-weight neonates |
| Content of Care | B.1.1 - Full blood count testing is the recommended method for diagnosing anaemia in pregnancy. In settings where full blood count testing is not available, on-site haemoglobin testing with a haemoglobinometer is recommended over the use of the haemoglobin colour scale as the method for diagnosing anaemia in pregnancy |
| Content of Care | B.1.2 - Midstream urine culture is the recommended method for diagnosing asymptomatic bacteriuria (ASB) in pregnancy. In settings where urine culture is not available, on-site midstream urine Gramstaining is recommended over the use of dipstick tests as the method for diagnosing ASB in pregnancy |
| Content of Care | B.1.4 - Hyperglycaemia first detected at any time during pregnancy should be classified as either gestational diabetes mellitus (GDM) or diabetes mellitus in pregnancy, according to WHO criteria |
| Content of Care | B.1.6 - Health-care providers should ask all pregnant women about their use of alcohol and other substances (past and present) as early as possible in the pregnancy and at every antenatal care visit |
| Content of Care | B.1.8 - In settings where the tuberculosis (TB) prevalence in the general population is 100/100 000 population or higher, systematic screening for active TB should be considered for pregnant women as part of antenatal care |
| Content of Care | B.2.4 - One ultrasound scan before 24 weeks of gestation (early ultrasound) is recommended for pregnant women to estimate gestational age, improve detection of fetal anomalies and multiple pregnancies, reduce induction of labour for post-term pregnancy, and improve a woman's pregnancy experience |
| Content of Care | C.2 - Antibiotic prophylaxis is only recommended to prevent recurrent urinary tract infections in pregnant women in the context of rigorous research |

For peer review only

| | |
|---|---|
| Content of Care | C.3 - Antenatal prophylaxis with anti-D immunoglobulin in non-sensitized Rh-negative pregnant women at 28 and 34 weeks of gestation to prevent RhD alloimmunization is only recommended in the context of rigorous research |
| Experience of Care | D.1 - Ginger, chamomile, vitamin B6 and/or acupuncture are recommended for the relief of nausea in early pregnancy, based on a woman's preferences and available options |
| Experience of Care | D.2 - Advice on diet and lifestyle is recommended to prevent and relieve heartburn in pregnancy. Antacid preparations can be offered to women with troublesome symptoms that are not relieved by lifestyle modification |
| Experience of Care | D.3 - Magnesium, calcium or non-pharmacological treatment options can be used for the relief of leg cramps in pregnancy, based on a woman's preferences and available options |
| Experience of Care | D.4 - Regular exercise throughout pregnancy is recommended to prevent low back and pelvic pain. There are a number of different treatment options that can be used, such as physiotherapy, support belts and acupuncture, based on a woman's preferences and available options |
| Experience of Care | D.5 - Wheat bran or other fibre supplements can be used to relieve constipation in pregnancy if the condition fails to respond to dietary modification, based on a woman's preferences and available options |
| Experience of Care | D.6 - Non-pharmacological options, such as compression stockings, leg elevation and water immersion, can be used for the management of varicose veins and oedema in pregnancy, based on a woman's preferences and available options |
| Health Systems | E.2 - Midwife-led continuity-of-care models, in which a known midwife or small group of known midwives supports a woman throughout the antenatal, intrapartum and postnatal continuum, are recommended for pregnant women in settings with well functioning midwifery programmes |
| Health Systems | E.3 - Group antenatal care provided by qualified health-care professionals may be offered as an alternative to individual antenatal care for pregnant women in the context of rigorous research, depending on a woman's preferences and provided that the infrastructure and resources for delivery of group antenatal care are available |
| Health Systems | E.4.1 - The implementation of community mobilization through facilitated participatory learning and action (PLA) cycles with women's groups is recommended to improve maternal and newborn health, particularly in rural settings with low access to health services. Participatory women's groups represent an opportunity for women to discuss their needs during pregnancy, including barriers to reaching care, and to increase support to pregnant women |
| Health Systems | E.4.2 - Packages of interventions that include household and community mobilization and antenatal home visits are recommended to improve antenatal care utilization and perinatal health outcomes, particularly in rural settings with low access to health services |
| Health Systems | E.5.1 - Task shifting the promotion of health-related behaviours for maternal and newborn health to a broad range of cadres, including lay health workers, auxiliary nurses, nurses, midwives and doctors is recommended |
| Health Systems | E.5.2 - Task shifting the distribution of recommended nutritional supplements and intermittent preventive treatment in pregnancy (IPTp) for malaria prevention to a broad range of cadres, including auxiliary nurses, nurses, midwives and doctors is recommended |
| Content of Care | Fetal heart rate monitored † |

† Established good clinical practice within ANC

For peer review only

## PRISMA-ScR Checklist

| Section | Item | PRISMA-ScR Checklist Item | Reported on page # |
|---|---|---|---|
| **Title** | | | |
| Title | 1 | Identify the report as a scoping review. | 1 |
| **Abstract** | | | |
| Structured summary | 2 | Provide a structured summary that includes (as applicable) background, objectives, eligibility criteria, sources of evidence, charting methods, results, and conclusions that relate to the review questions and objectives. | 2 |
| **Introduction** | | | |
| Rationale | 3 | Describe the rationale for the review in the context of what is already known. Explain why the review questions/objectives lend themselves to a scoping review approach. | 4 |
| Objectives | 4 | Provide an explicit statement of the questions and objectives being addressed with reference to their key elements (e.g., population or participants, concepts, and context) or other relevant key elements used to conceptualize the review questions and/or objectives. | 4 |
| **Methods** | | | |
| Protocol and registration | 5 | Indicate if a review protocol exists; state if and where it can be accessed (e.g., a Web address); and if available, provide registration information, including the registration number. | 14 |
| Eligibility criteria | 6 | Specify characteristics of the sources of evidence (e.g., years considered, language, publication status) used as criteria for eligibility, and provide a rationale. | 5-7 |
| Information sources * | 7 | Describe all information sources in the search (e.g., databases with dates of coverage and contact with authors to identify additional sources), as well as the date the most recent search was executed. | 5, 7 |
| Search | 8 | Present full electronic search strategy for at least one database, including any limits used, such that it could be repeated. | 5 |

For peer review only

| Section | Item | PRISMA-ScR Checklist Item | Reported on page # |
|---|---|---|---|
| Selection of sources of evidence § | 9 | State the process for selecting sources of evidence (i.e., screening and eligibility) included in the scoping review. | 5-7 |
| Data charting process | 10 | Describe the methods of charting data from the included sources of evidence (e.g., calibrated forms or forms that have been tested by the team before their use, and whether data charting was done independently or in duplicate) and any processes for obtaining and confirming data from investigators. | 5-6 |
| Data items | 11 | List and define all variables for which data were sought and any assumptions and simplifications made. | 6 |
| Critical appraisal of individual sources of evidence | 12 | If done, provide a rationale for conducting a critical appraisal of included sources of evidence; describe the methods used and how this information was used in any data synthesis (if appropriate). | Not done |
| Summary measures | 13 | Not applicable (n/a) for scoping reviews. | n/a |
| Synthesis of results | 14 | Describe the methods of handling and summarizing the data that were charted. | 6-7 |
| Risk of bias across studies | 15 | Not applicable for scoping reviews. | n/a |
| Additional analyses | 16 | Not applicable for scoping reviews. | n/a |
| **Results** | | | |
| Selection of sources of evidence | 17 | Give numbers of sources of evidence screened, assessed for eligibility, and included in the review, with reasons for exclusions at each stage, ideally using a flow diagram. | 8 |
| Characteristics of sources of evidence | 18 | For each source of evidence, present characteristics for which data were charted and provide the citations. | Supplementary Table A1 |
| Critical appraisal within sources of evidence | 19 | If done, present data on critical appraisal of included sources of evidence (see item 12). | Not done |
| Results of individual sources of evidence | 20 | For each included source of evidence, present the relevant data that were charted that relate to the review questions and objectives. | Supplementary Table A1 |
| Synthesis of results | 21 | Summarize and/or present the charting results as they relate to the review questions and objectives. | 8-11 |

For peer review only

| Section | Item | PRISMA-ScR Checklist Item | Reported on page # |
|---|---|---|---|
| Risk of bias across studies | 22 | Not applicable for scoping reviews. | n/a |
| Additional analyses | 23 | Not applicable for scoping reviews. | n/a |
| **Discussion** | | | |
| Summary of evidence | 24 | Summarize the main results (including an overview of concepts, themes, and types of evidence available), link to the review questions and objectives, and consider the relevance to key groups. | 12 |
| Limitations | 25 | Discuss the limitations of the scoping review process. | 3, 13 |
| Conclusions | 26 | Provide a general interpretation of the results with respect to the review questions and objectives, as well as potential implications and/or next steps. | 12-13 |
| **Funding** | | | |
| Funding | 27 | Describe sources of funding for the included sources of evidence, as well as sources of funding for the scoping review. Describe the role of the funders of the scoping review. | 14 |

* Where *sources of evidence* (see second footnote) are compiled from, such as bibliographic databases, social media platforms, and Web sites.

§ A more inclusive/heterogeneous term used to account for the different types of evidence or data sources (e.g., quantitative and/or qualitative research, expert opinion, and policy documents) that may be eligible in a scoping review as opposed to only studies. This is not to be confused with information sources (see first footnote).

For peer review only
